# Effects of an Offshore Wind Farm (OWF) on the Common Shore Crab *Carcinus maenas*: Tagging Pilot Experiments in the Lillgrund Offshore Wind Farm (Sweden)

**DOI:** 10.1371/journal.pone.0165096

**Published:** 2016-10-25

**Authors:** Olivia Langhamer, Håkon Holand, Gunilla Rosenqvist

**Affiliations:** Department of Biology, Norwegian University of Science and Technology, Trondheim, Norway; Academia Sinica, TAIWAN

## Abstract

Worldwide growth of offshore renewable energy production will provide marine organisms with new hard substrate for colonization in terms of artificial reefs. The artificial reef effect is important when planning offshore installations since it can create habitat enhancement. Wind power is the most advanced technology within offshore renewable energy sources and there is an urgent need to study its impacts on the marine environment. To test the hypothesis that offshore wind power increases the abundance of reef species relative to a reference area, we conduct an experiment on the model species common shore crab (*Carcinus maenas*).Overall, 3962 crabs were captured, observed, marked and released in 2011 and 1995 crabs in 2012. Additionally, carapace size, sex distribution, color morphs and body condition was recorded from captured crabs. We observed very low recapture rates at all sites during both years which made evaluating differences in population sizes very difficult. However, we were able to estimate population densities from the capture record for all three sites. There was no obvious artificial reef effect in the Lillgrund wind farm, but a spill-over effect to nearby habitats cannot be excluded. We could not find any effect of the wind farm on either, morphs, sex distribution or condition of the common shore crab. Our study found no evidence that Lillgrund wind farm has a negative effect on populations of the common shore crab. This study provides the first quantitative and experimental data on the common shore crab in relation to offshore wind farms.

## Introduction

As the world is trying to make a transition to a lower carbon economy, offshore wind power capacity is expected to grow significantly [1]. Offshore wind energy has a great resource potential as the wind there is higher and steadier than onshore. By the end of 2015 there were 7748 MW installed and 3198 under construction, compromising 76 wind farms spread over ten European countries [2]. Although the advantages of renewable energy on a global scale are not in doubt, the effects on the local environment must also be carefully considered [3].

Offshore wind turbines will affect local hydrodynamics, and thus change larval and nutrient transports [4, 5]. Furthermore, analytical models suggest that large farms may act as small mountain-chains creating up- or down-welling velocities influencing the atmosphere [6]. On the other hand, offshore wind turbines may act as artificial reefs [7] due to their foundations and potential scour protections. This may increase the amount of habitat available for local fish and invertebrate assemblages, and enhance the local biomass of sessile and mobile species [3, 8–10]. Artificial reefs have become an important and popular resource enhancement technique, as they are thought to improve fish stocks and attract fish [11–14]. The effect of artificial reefs on recruitment rates may be species specific due to differences in limitation by refuge, food, territory and/or behavioral requirements [3]. Artificial reefs that enhance growth, reproduction and survival of individual species can thus have an important effect on population structure and dynamics [15, 16]. Furthermore, the specific design of marine renewable energy installations can affect their impact on the environment, for example by attracting reef aggregated species [17–21]. As commercial fishing is restricted or prohibited within offshore wind farms, establishment of marine renewable energy installations may enhance fish populations, fish size and species richness in a degree comparable to marine protected areas [22]. When a marine area becomes protected by no-fishing zones around marine renewable energy installations it may lighten the pressure from fisheries on commercial and/or overexploited species [8]. However, the installation of marine renewable energy devices may also include a loss or degradation of the local habitat. The degree of impact will vary depending on the type and size of the installation [3], and although offshore marine renewable energy installations in general are considered unlikely to generate significant habitat loss, it is thought that inappropriate siting has the potential to cause deleterious effects for some taxa [23].

In connection to an environmental monitoring program of fish and mobile benthic species in Lillgrund offshore wind farm (see Methods section 2.1 for a description of the farm), the Swedish Agency for Marine and Water Management observed a tendency of an increasing quantity of shore crabs in Lillgrund after the wind farm was constructed [24]). These observations in the offshore wind farm indicated that artificial hard substrates can have an impact on natural shore crab population`s distribution. A great variability in behavior, morphology and physiology is commonly found in *Carcinus maenas* in relation to size, carapace coloration and sex [25, 26, 27]. This variability reflects the phenotypic adaptive responses of individual crabs in relation to differences in the environment [28–30], and are expressed when organisms experience different ecological and environmental conditions [31]. As shore crabs inhabit a variety of habitats, it has been suggested as a useful model organism to examine how habitat quality affects individual and population level parameters [32].

The overall aim of this study was to investigate the effect of offshore wind farms on local populations of shore crabs. Specifically, we wanted to test the notion that shore crab populations would be more abundant in the Lillgrund wind farm compared to adjacent control sites due to added new habitat. In addition, we investigated possible differences in morphological traits (e.g. carapace size) and sex ratio between the site containing wind turbines and control sites.

## Material and Methods

### Study site

Lillgrund offshore wind farm (OWF) is situated in the Öresund strait, between Sweden and Denmark, about 7 km off coast of Sweden and 9 km off the coast of Denmark (55° N, 12° W). It consists of 48 offshore turbines that have been operating since the beginning of 2008. The total height of the wind turbines is 115 m, and the turbines have a rotor diameter of 90 m. The seabed in this area consists of limestone, stones and sand, and the depth varies from 4–9 meters [33]. Because of differences in salinity between the water masses coming from the brackish Baltic Sea in the south and the salt Atlantic Ocean in the north, the salinity in Öresund is related to the water currents [24], but ranges normally from 8–15 ‰ down to 10–15 m depth [34].

**Fig 1:**
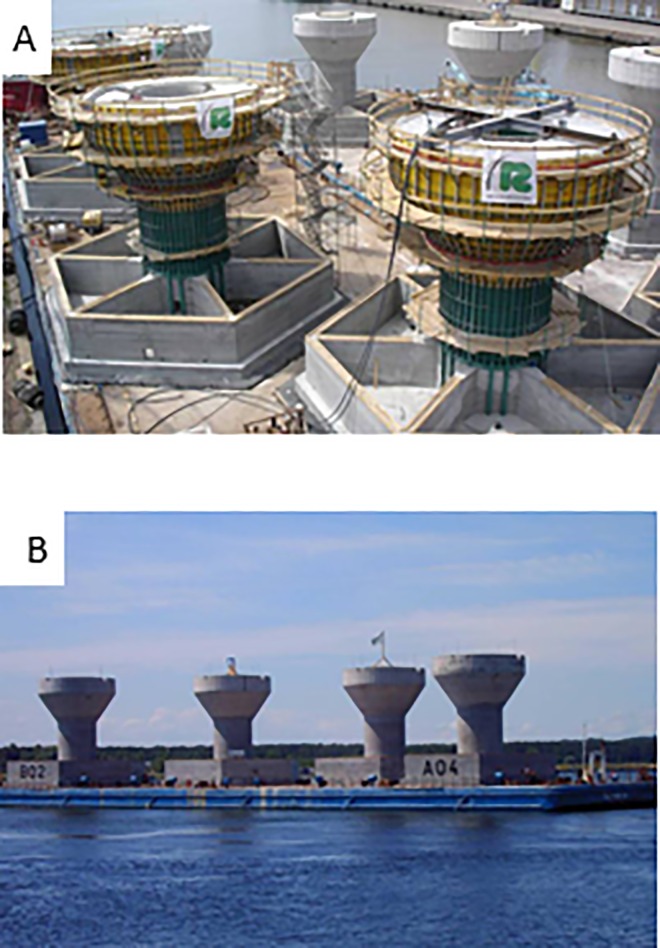
Photographic illustration of the gravity based foundations in the Lillgrund offshore wind farm: A. Six-sided bottom slab of the foundations; the six pockets and circular section in the shaft are filled with ballast. B. Transportation of the gravity based foundations for deployment in Lillgrund. With kind support from RSB Formwork Technology.

The foundations of the wind turbines in Lillgrund are gravity based. As the seabed in the area is not all flat, five different foundations with different heights were used, but all had a footage diameter of 19 m^2^ [35]. That means that 15% of Lillgrund`s area is covered by artificial hard substrate. The bottom slab of the foundations is six-sided, where each side is approximately 8 m (Fig 1A and 1B). The whole area of the seabed around each foundation is covered with a rock-filled scour protection to prevent the ocean currents from undermining the stability of the foundations by moving seabed material [36]. This rock-filled scour protection may provide an ideal habitat for reef aggregated species, such as shore crabs.

### Study species

The shore crab *Carcinus maenas* is among the best known intertidal animals. The fact that it is common, relatively large, easily found; measured; sex-determined and marked, makes it highly suitable for field studies [37]. The shore crab is a widely distributed epibenthic species of European coast and estuaries, and inhabits both soft and hard habitat [38]. The species has also been found in the north-western Atlantic, and has recently colonized some areas in Australia, southern Africa and the Pacific coast of North America [39]. It is considered to be one of the worst invasive species in the world [40].

The life cycle of the shore crab is complex with four pelagic zoea stages and a megalopal (postlarval) stage that settle and metamorphose into the first benthic crab stage [41]. *C*. *maenas* has been shown to consume an enormous variety of prey items, especially organisms belonging to the phyla Crustacea, Annelida and Mollusca [39]. It lives in both salt and brackish waters and on hard and soft substrate [42]. Although the shore crab is most common on the coast and estuarine habitat, it has been found down to depths of 200 meters [42]. The coloration of shore crab varies from a green to red carapace with darker marmoration and a yellow abdomen, and can reach a carapace width of 80 mm [42]. The different carapace color is an indicator of intermoult duration, with red crabs having been in intermoult longer than the green ones [31].

### Sampling and Experimental design

**Fig 2:**
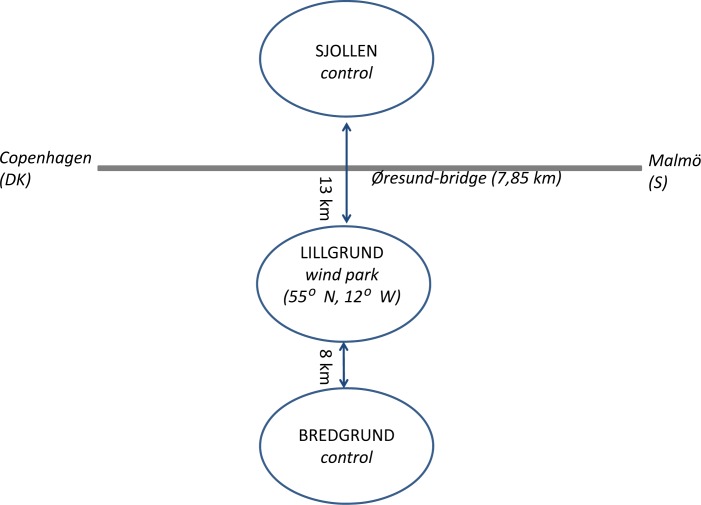
Location of Lillgrund OWF and the two control sites, Sjollen in the North and Bredgrund in the South.

The project was carried out in Lillgrund OWF and two control sites, Sjollen situated 13 km north, and Bredgrund situated 8 km south of Lillgrund (Fig 2). Both control sites are similar to Lillgrund in physical characteristics, with seabed consisting of limestone, stones and sand, and depth varying from 4–9 meters. Each of the three areas covers about 6000 m^2^. The two control areas are influenced by the same strong salinity gradient that is occurs throughout the sound [43]. The Sjollen site is situated closer to shipping lanes than either of the two other sites.

Shore crabs were captured during two summers (25th July–9th August 2011 and 27th June–20th July 2012), with two linked double fyke nets at 10 randomized locations in the Lillgrund OWF and two control sites, i.e. at 30 stations altogether). The same locations and fishing-methods were used by Swedish Agency for Marine and Water Management in studies of the effect of the wind farm in Lillgrund on fish and fisheries [44]. The fyke nets had a height of 55 cm; three entrances and were five meter long. At each station the nets were set out during the morning and collected and emptied the following morning. On days with strong wind or strong current, the nets were left for two days or in one case, until later in the evening. In Lillgrund the fyke nets were placed out on the soft bottom between the foundations. Bait was only added in 2012 after all stations in the areas had been visited twice and was used throughout the rest of the project to increase the catch yield.

To estimate population size and density at each site, a mark- recapture method was applied with two different marking methods in 2011 and 2012 due to equipment availability. Both marking techniques are expected to give the same results and have been used in earlier crab experiments [45] The crabs caught in 2011 were marked individually with cable tie placed around the merus segment of the first periopod (claw) in a way that didn't hinder the crabs natural behavior. Crabs caught in 2012 were marked with t-bar anchor floy tags [46] on the posterior margin of the epimural suture. In addition all captured crabs were sex determined, location and presence of eggs were noted, crabs were measured (mm) and released at the same location as they were caught [47]. The size of the crabs was measured as the greatest width of their carapace (mm). The majority of the captured individuals smaller than 42 mm were not marked since they were too small, but counted. Any damage or loss of legs or claws on the caught crabs was recorded. Crabs possessing any of these characteristics were classified as being in “reduced body condition”, while those with no damage or reduced number of legs or claws were classified as being in “good body condition”. The coloration of the crabs was classified as “green” or “red”, as recommended by McGaw and Nailor [28]. All handling was done directly on the boat. Mean individual handling time was ca. 1 minute, and all individuals were released within 1 hour in the same location they were caught. Any recapture of previously caught and marked individuals was recorded. At each location in the three areas, four marking and five capture occasions took place in 2011, and in 2012 five marking and six capture occasions took place. Catch per unit effort was standardized as the mean number of crabs caught in each station of the areas per day per double fyke net. The catch per unit effort index has earlier been shown to represent useful information concerning relative abundances of a population [48]. Ethical permissions for fishing crabs with fyke nets were given by the Swedish Board of Fisheries (Ref. nr. 23-2231-11), and crabs were treated and tagged with care and good handling procedures in order to prevent harm and damage. Vattenfall AB gave the permission to conduct the field studies during both 2011 and 2012 at Lillgrund wind farm. *C*. *maenas* is not an endangered or protected species, and thus no specific permissions were required for the public control sites Bredgrund and Sjollen.

### Data analyses

Statistical analyses were performed with R-software version 3.0.2. Data fitted the assumption of normality and homoscedasticity for parametric testing.

Two-way analysis of variance (ANOVA) with Tukey`s HSD as post hoc test was used to analyze the effect of area on the catch per unit effort indexes in the three different sites, Lillgrund, Sjollen and Bredgrund in the years 2011 and 2012. Population estimations were conducted with the program MARK 6.2 where marked individuals only should be used for the analysis [49]. The dataset was treated as a closed captures in the analysis, as the collection of data was done in a short time span each year. The density (individuals/m^2^) was calculated from the estimated population size.

Multifactorial ANOVA with Tukey`s HSD as *post hoc test* was used to analyze mean size of shore crabs between the sites, years, males and females and color morphs with their interactions. Logistic regression was used to investigate relationship between categorical data (function = glm, family = binomial, link = logit). Data were analyzed in three different models. Model 1: Coloration (red or green) was treated as response variable with sites and years as explanatory variable. Model 2: Body condition (good or reduced) was treated as response variable with sites and years as explanatory variable. Model 3: Sex distribution (male or female) was treated as response variable with sites and years as explanatory variable.

## Results

### Population abundance

**Fig 3:**
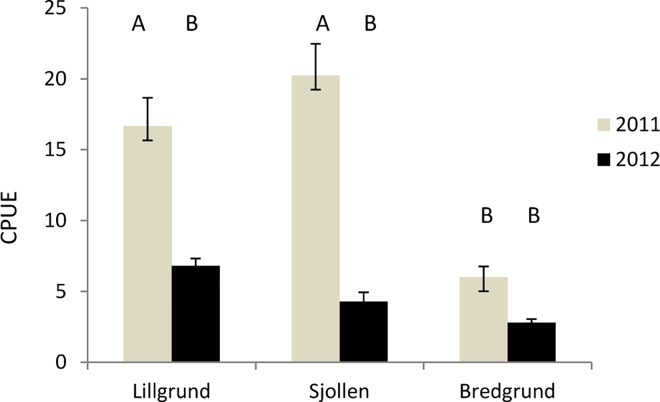
Calculated mean± SE in catch per unit effort (CPUE) of the common shore crab, C*arcinus maenas* in Lillgrund OWF and the two control sites Bredgrund and Sjollen in 2011 and 2012. Letters above bars indicate significantly different means based on Tukey`s post-hoc comparisons.

Mean catch per unit effort ranged between 2,8 ± 0,75 and 20,2 ± 2,2 individuals per fyke net per day (Fig 3). Table 1 shows that there was a significant interaction between site and year. Catch per unit effort was signifantly higher in year 2011 compared to 2012 and differed significantly among sites where Lillgrund offshore wind farm (OWF) and Sjollen had a higher CPUE in 2011 than Bredgrund (Fig 3, Table 2).

**Table 1:** Multifactorial analyses of variance (ANOVA) of mean carapace width of female and male common shore crabs with different color morphs (green, red), in different body conditions (good, reduced) at three different sites (Lillgrund OWF, Bredgrund, Sjollen) in two different years (2011, 2012).

Factor	df	MS	F	p
**site**	2	3382	91,9	***<0*,*001***
**year**	1	3098	84,2	***<0*,*001***
**color**	1	6513	177,0	***<0*,*001***
**sex**	1	23268	632,5	***<0*,*001***
**condition**	1	6	0,16	0,69
**site:yea**	2	857	23,3	***<0*,*001***
**site:color**	2	494	13,4	***<0*,*001***
**year:color**	1	305	8,2	***0*,*004***
**site:sex**	2	19	0,5	0,60
**year:sex**	1	202	5,5	***0*,*02***
**color:sex**	1	95	2,6	0,11
**site:condition**	2	51	1,4	0,10
**year:condition**	1	46	1,3	0,26
**sex:condition**	1	36	1,0	0,32
**color:condition**	1	38	1,0	0,31
**site:year:color**	2	39	1,1	0,34
**site:year:sex**	2	13	0,4	0,70
**site:color:sex**	2	64	1,7	0,18
**site:sex:condition**	2	203	5,5	***0*,*004***
**year:color:sex**	1	85	2,3	0,13
**site:year:condition**	2	8	0,2	0,8
**year:sex:condition**	1	122	3,3	0,07
**color:sex:condition**	1	39	3,8	0,05
**site:color:condition**	2	108	2,9	0,05
**year:color:condition**	1	0	0,01	0,93
**site:year:color:sex**	1	37	1,0	0,32
**site:year:color:sex:condition**	2	7	0,2	0,83
**Residuals**	4149	37		

**Table 2:** Comparison of Catch per Unit Effort (CPUE) using univariate analyses of variance (ANOVA) of the common shore crab in three different sites (Lillgrund OWF, Bredgrund, Sjollen) and in different years (two levels; 2011, 2012).

	df	MS	F	p
**Site**	2	360.2	21.90	<0.001
**Year**	1	1347.3	81.92	<0.001
Site*Year	2	197.8	12.03	<0.001
**Residuals**	53	16.4		

### Population size estimation and density

Overall, 3962 shore crabs were captured in 2011 and 1995 crabs in 2012 (Table 3). Recapture rates were highest in Lillgrund OWF and capture probability (p-hat) was higher in 2011 compared to 2012 (Table 4). Local population size reached a minimum of 18 085 crabs in Lillgrund in 2011 and a maximum of 60 152 crabs in Lillgrund in 2012 (Table 4). Estimated population densities ranged between 3,01 and 10 individuals per m^2^ in Lillgrund and the control sites (Table 4).

**Table 3:** Mark and recapture data for shore crab populations in Lillgrund OWF, Sjollen and Bredgrund in 2011 and 2012.

Site, year	Captured (n)	Marked (n)	Recaptured (n)
**Lillgrund 2011**	1939	944	19
**Sjollen 2011**	1517	901	7
**Bredgrund 2011**	506	332	1
**Lillgrund 2012**	1004	696	8
**Sjollen 2012**	627	441	0
**Bredgrund 2012**	364	204	1

**Table 4:** Common shore crab: population size estimations, capture probability and density estimations in Lillgrund OWF, Sjollen and Bredgrund in 2011 and 2012.

	Recaptures (%)	Marked (n)	N	p-hat	Density (ind / m^2^)
**Lillgrund 2011**	2,01	944	18085 ± 4051	0,013	3,01
**Sjollen 2011**	0,78	901	43 836 ± 16 389	0,005	7,31
**Bredgrund 2011**	0,3	332	41 440 ±-41 285	0,002	6,91
**Lillgrund 2012**	1,15	696	60 152 ± 22 560	0,003	10,0
**Bredgrund 2012**	0,49	204	55 808 ± 56 562	0,001	9,3

### Morphological traits

**Fig 4:**
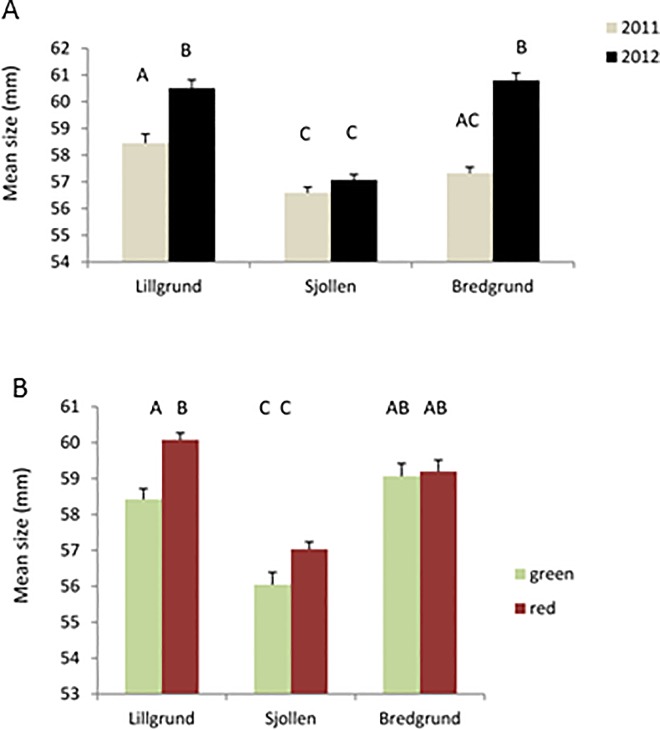
Calculated mean± SE in carapace size of the common shore crab, *Carcinus maenas* in A. Lillgrund OWF and the two control sites Bredgrund and Sjollen in 2011 and 2012, and B. in the two morphs, red and green in Lillgrund OWF and the two control sites Bredgrund and Sjollen. Letters above bars indicate significantly different means based on Tukey`s post-hoc comparisons.

**Fig 5:**
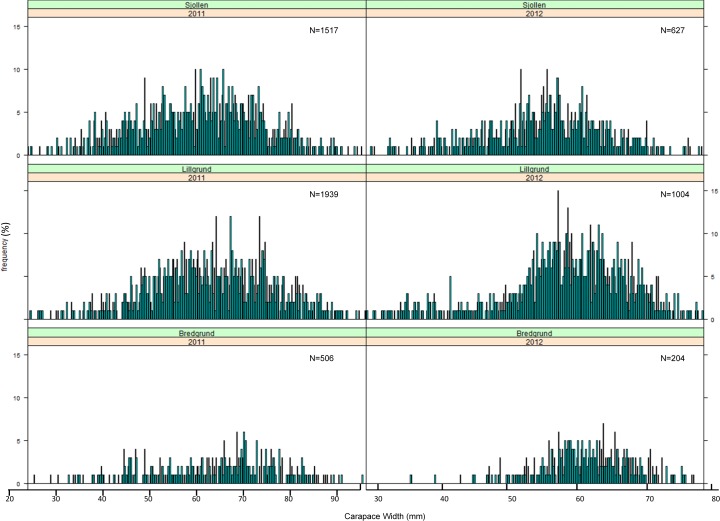
Size frequency distribution of CW in each site (Lillgrund OWF, Bredgrund and Sjollen) sampled in summer 2011 and 2012. N, number of measured individuals.

Mean carapace size varied in all populations with the widest mean carapace size in Bredgrund and the smallest in Sjollen (Table 1, Fig 4A). There was a higher variation in mean carapace size between both years with smaller crabs caught in 2011 than in 2012 (Table 1, Fig 4A and Fig 5). Testing the effect of color on mean size showed a significant difference between color morphs and areas in carapace width with the largest red crabs in Lillgrund OWF and the smallest green crabs in Sjollen (Table 1, Fig 4B). Furthermore, in year 2011 both green and red crabs were smaller than in 2012 (Fig 4B). In general, green crabs were significantly smaller than red ones (57,8 mm ±0,18 mm and 58,7 mm ±0,13 mm respectively, Table 1).

**Fig 6:**
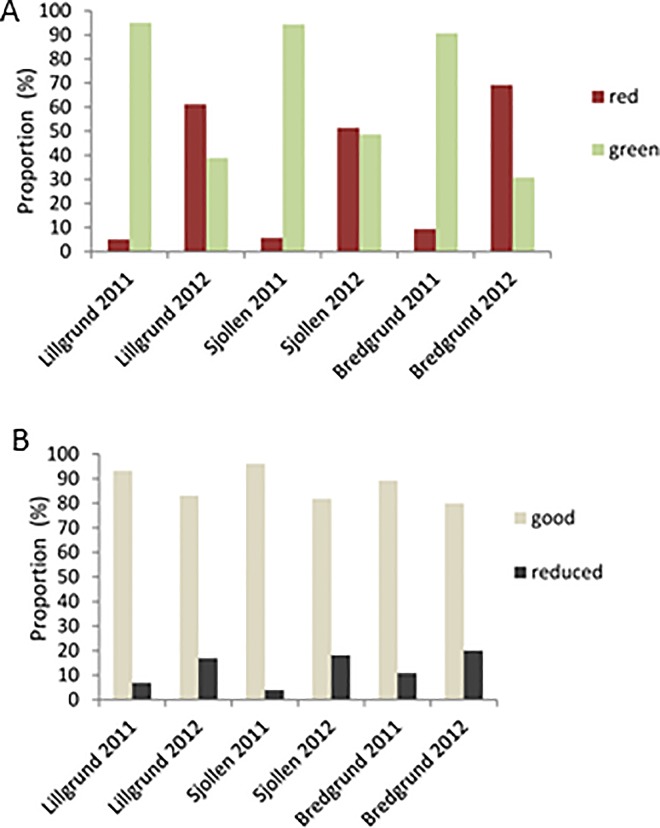
Proportion estimates from A. model 1. Coloration (red and green) of shore crabs in Lillgrund OWF and the two control sites Sjollen and Bredgrund in the years 2011 and 2012 and, B. from model 2. Body condition (good or reduced) of shore crabs in Lillgrund OWF and the two control sites Sjollen and Bredgrund in the years 2011 and 2012.

The results of model 1 showed that the proportion of green crabs varied between sites (lowest in Bredgrund, highest in Sjollen), and between years (lower in the 2012) (Fig 6A).

Model 2 tested the body condition (good/reduced) of the caught crabs described by any observed damage or loss of legs or claws. Body condition varied from 88,1% of all shore crabs being in good condition in Lillgrund to 88,9% in Sjollen, and both differed significantly from Bredgrund (84,5%) (Fig 6B). There was a significant difference among years with 92,8% in good body condition in 2011 and 81,6% in good body condition in 2012 (Fig 4B). There was an interactive effect in mean carapace size comparing body condition in male and female crabs in the three sites (Table 1).

The results of model 3 showed that sex ratio in crabs varied between 95,5% male crabs in Bredgrund, 95,6% in Lillgrund and 96,4% in Sjollen (Table 1), and there was no significant difference in mean carapace size comparing male and female crabs in the three sites (Table 1). Testing the effect sex ratio on mean size showed a significant difference between sex ratio and years in mean carapace width with the largest males in 2012 (60,3 mm ±0,13 mm) and the smallest female in 2012 (46,5 mm ±0,47 mm (Table 1).

## Discussion

In this study we did not observe any significant effect, negative or positive, on the common shore crab from the Lillgrund offshore wind farm (OWF). We could not find any clear artificial reef effect on shore crab densities at the Lillgrund wave farm. This study also shows the difficulties to estimate population size on a species with a large population size.

The higher catch per unit efforts of shore crabs in 2011 in Lillgrund and in the adjacent control site Sjollen compared to Bredgrund can be due to the fact that both Lillgrund and Sjollen have higher salinities than Bredgrund, and thus very similar conditions for mobile species. Fyke nets may be a good food resource by the catch of fish such as cod, wrasse, gobies etc. attracting shore crabs, so that an aggregation at the nets may take place in both Lillgrund and Sjollen (personal observations), however, no records of bycatch were done. If we compare the CPUE data from a longer term perspective of 10 years, there is no difference in abundance of shore crabs in Lillgrund compared to the control areas [24]. In terms of stock assessment, it is the inter-annual variation of abundance that is of most interest [50]. All in all the yearly variation in CPUE is high, and very likely depending on changed conditions, such as wind, current, turbulence, temperature and salinity. Shore crabs seem to avoid both too high and too low temperatures and regulate this by migration to locations of preferred temperature. This migration can influence the catch per unit effort, and the strong association between temperature and catch per unit effort may mask the underlying changes in abundance [50].

**Fig 7:**
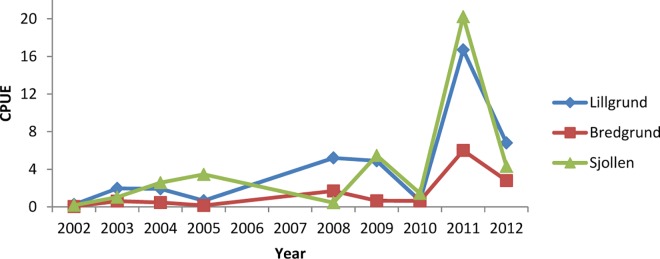
Mean catch per unit effort in Lillgrund OWF, Sjollen and Bredgrund during the years of construction and during operation of the wind farm. Catch per unit effort for all years from 2002 and 2010 are calculated from data from Swedish Agency for Marine and Water Management [24], data from 2011 and 2012 are added from our results.

After the wind farm was established in 2007 the abundance of shore crabs in all three study sites has increased (Fig 7). This was also noted by Bergström et al. [24], and was suggested to be a signal of a change of greater scale in the ecosystem. An increase in number of caught crabs during fishing has also been observed by Sheehan et al. [51] in a survey on the effects of a crab-fishery method “crab-tiling” (i.e. artificial shelters providing stable structures) on the abundance of shore crab on intertidal sand- and mudflats in estuaries. They found an increase in the abundance of shore crab in the areas where this kind of fishery occurred, which is thought counter-intuitively from what would normally be expected in a population that is fished [51]. This increase in abundance was associated with an increase in available habitat, created by the “crab-tiling”.

Similar observations have been done by Göransson [52], as there has been found changes in species compositions in Öresund from early 1900s to surveys performed in 1989–2000. Eutrophication, parallel with increased nutrient load to coastal waters [52] might have a stronger effect on the abundance of shore crabs than lack of space. In the long run higher abundance of shore crabs may reduce the abundance of preferred prey species to a very low level while gradually shifting to alternative prey [53]. Predating shore crab has been shown to have an effect on recruitment success of cockles, *Cerastoderma edule* [54], blue mussels, barnacles and diatoms [53]. Shore crabs have been shown to have a negative impact on recruitment of periwinkles *[55]*. On the other hand, shore crabs are prey items for other species, such as cod, an increase in the population may be positive for species in higher trophic levels [56].

Our study shows that it is not possible to make an evaluation of differences in population size between Lillgrund OWF and the two control sites based on the population size estimation done in this study; the reason is the very low recapture rate. Earlier capture-recapture studies on *C*. *maenas* in both Europe and the US showed far higher recapture rates than we received with similar marking methods [57, 58]. An earlier study by McPherson [59] on survival of Blue swimmer crab marked with t-bar anchor floytags on the posterior suture of the carapace, found no change in behavior of tagged crabs that could be related to the tagging. The Blue swimmer crab is also an intertidal species with similar life cycle and feeding behavior as our model species, *C*. *maenas*, as well as easily adaptable to changing environmental conditions *[60]*. This mêns that we can with good confidence exclude the possibility that low recapture rates are caused by change in behavior or mortality of tagged individuals. Neither was the survival of the tagged crabs severely reduced: only one out of 24 crabs tagged with a standard t-bar anchor tag died a short time after tagging [59]. Our density results for shore crabs in Lillgrund and both control sites lie within the range of other earlier reported adult shore crab (30–70 mm) in Europe, and varies between 0.1 to 20 individuals per square meter in suitable habitats during summer [47, 61].

We did find a size differences both between the sites, but not between the wind farm and the other sites. In Sjollen, crab size has been smaller than those in Lillgrund and Bredgrund and crabs were larger in 2012 than in 2011. Smaller mean size of crabs in Sjollen may be explained by stress induced by noise pollution from passing ships [62]. The ship traffic in the Baltic Sea is considerable, and has increased significantly the last ten years [63]. Sjollen is more exposed to ship noise than Lillgrund and Bredgrund as it is situated close to the shipping lane through the Öresund strait. Wale et al. [62] showed size-dependent physiological responses of shore crabs to single and repeated playback of ship noise. Crabs exposed to ship-noise playback had higher oxygen consumption than those experiencing playback of ambient harbor noise. The response of shore crabs to a single ship-noise was size dependent, with larger individuals showing a stronger response (i.e. consuming proportionately more oxygen) than the smaller ones.

Observed differences in crab size dependent on color morphs can be explained by difference in behavior and food preferences. In general, red individuals prefer larger bivalves and are stronger and more aggressive compared to green individuals [64]. In an earlier study it has been documented a high amount of bivalves, especially of blue mussels, *Mytilus edulis*, in the Lillgrund wind park [65].

We could not observe any effect of the offshore wind farm on body condition of shore crabs measured as any observed damage or loss of legs or claws. Carapace width in crabs being in reduced body condition was generally larger than the carapace width in crabs in good body condition. A thicker and larger exoskeleton is an advantage in male-male interactions, but it may reduce the crab`s ability to tolerate salinity changes [31, 66]. This can be of importance, since the salinity in Öresund varies due to the water currents coming either from the brackish Baltic Sea in the south or the salt Atlantic Ocean in the north. The poor representation of female in the samples (about 6% females of all crabs caught) can likely be explained by the fact that adult female crabs behave different from males in the summer where females prefer deeper waters and differ in feeding activities [47, 67]. Such behavioral patterns can reduce the catch of female crabs by traps compared to male crabs during this time of year. As no difference in proportion of males was found between Lillgrund OWF and the control sites we suggest that the presence of wind turbines does not have an effect on the sex ratio of shore crabs.

This study provides the first quantitative and experimental data on the common shore crab in relation to offshore wind farms. However, further (long term) studies are needed to shed light on the long term effects that wind turbines may have on population dynamics of marine organisms other than shore crabs. Follow-up studies and targeted monitoring may also further reveal the effects of offshore wind production on the marine ecosystem as a whole.

## Supporting Information

S1 Table Dataset of captured *Carcinus maenas*, including ID, Year, Site, Station, CarapaceWidth, Sex, Color and Body Condition. (PDF)

## References

[1] Greening Blue Energy: Identifying and managing the biodiversity risks and opportunities of offshore renewable energy, (2010).

[2] Rodrigues S, Restrepo C, Kontos E, Teixeira Pinto R, Bauer P. Trends of offshore wind projects. Renewable and Sustainable Energy Reviews. 2015;49:1114–35.

[3] Inger R, Attrill MJ, Bearhop S, Broderick AC, Grecian WJ, Hodgson DJ, et al Marine renewable energy: potential benefits to biodiversity? An urgent call for research. Journal of Applied Ecology. 2009 12;46(6):1145–53.

[4] Breitburg DL, Palmer MA, Loher T. Larval Distributions and the Spatial Patterns of Settlement of an Oyster Reef Fish—Responses to Flow and Structure. Marine Ecology-Progress Series. [Article]. 1995 9;125(1–3):45–60.

[5] Snelgrove PVR, Butman CA. ANIMAL SEDIMENT RELATIONSHIPS REVISITED—CAUSE VERSUS EFFECT. In: Ansell AD, Gibson RN, Barnes M, editors. Oceanography and Marine Biology, Vol 32: An Annual Review1994. p. 111–77.

[6] Brostrom G. On the influence of large wind farms on the upper ocean circulation. Journal of Marine Systems. 2008 11;74(1–2):585–91.

[7] Petersen JK, Malm T. Offshore windmill farms: Threats to or possibilities for the marine environment. Ambio. 2006 3;35(2):75–80. 16722252 10.1579/0044-7447(2006)35[75:owftto]2.0.co;2

[8] Wilhelmsson D, Langhamer O. The Influence of Fisheries Exclusion and Addition of Hard Substrata on Fish and Crustaceans In: Shields MA, Payne AIL, editors. Marine Renewable Energy Technology and Environmental Interactions Humanity and the Sea. Dordrecht: Springer; 2014 p. 49–60.

[9] Stenberg C, Støttrup JG, Van Deurs M, Berg CW, Dinesen GE, Mosegaard H, et al Long-term effects of an offshore wind farm in the North Sea on fish communities. Marine Ecology Progress Series. [Article]. 2015;528:257–65.

[10] Reubens JT, Degraer S, Vincx M. The ecology of benthopelagic fishes at offshore wind farms: A synthesis of 4 years of research. Hydrobiologia. [Article]. 2014;727(1):121–36.

[11] Bohnsack JA, Sutherland DL. Artificial Reef Research—a Review with Recommendations for Future Priorities. Bulletin of Marine Science. 1985;37(1):11–39.

[12] Pickering H, Whitmarsh D. Artificial reefs and fisheries exploitation: A review of the 'attraction versus production' debate, the influence of design and its significance for policy. Fisheries Research. 1997 7;31(1–2):39–59.

[13] Dafforn KA, Glasby TM, Airoldi L, Rivero NK, Mayer-Pinto M, Johnston EL. Marine urbanization: an ecological framework for designing multifunctional artificial structures. Frontiers in Ecology and the Environment. 2015 3;13(2):82–90.

[14] Lowry MB, Glasby TM, Boys CA, Folpp H, Suthers I, Gregson M. Response of fish communities to the deployment of estuarine artificial reefs for fisheries enhancement. Fisheries Management and Ecology. 2014;21(1):42–56.

[15] Gibson RN. IMPACT OF HABITAT QUALITY AND QUANTITY ON THE RECRUITMENT OF JUVENILE FLATFISHES. Netherlands Journal of Sea Research. 1994 7;32(2):191–206.

[16] Beck MW, Heck KL, Able KW, Childers DL, Eggleston DB, Gillanders BM, et al The identification, conservation, and management of estuarine and marine nurseries for fish and invertebrates. Bioscience. 2001 8;51(8):633–41.

[17] Langhamer O. Artificial Reef Effect in relation to Offshore Renewable Energy Conversion: State of the Art. Scientific World Journal. 2012 2012. 10.1100/2012/386713 PMC3541568 23326215

[18] Wilhelmsson D, Malm T, Ohman MC. The influence of offshore windpower on demersal fish. Ices Journal of Marine Science. 2006 6;63(5):775–84.

[19] Wilhelmsson D, Malm T. Fouling assemblages on offshore wind power plants and adjacent substrata Estuarine Coastal and Shelf Science. 2008;79:459–66.

[20] Langhamer O, Wilhelmsson D. Colonisation of fish and crabs of wave energy foundations and the effects of manufactured holes—a field experiment. Mar Environ Res. 2009;68:151–7. doi: 10.1016/j.marenvres.2009.06.003 19560811 10.1016/j.marenvres.2009.06.003

[21] Langhamer O, Wilhelmsson D, Engström J. Artificial reef effect and fouling impacts on offshore wave power foundations and buoys—a pilot study. Estuarine, Coastal and Shelf Science. 2009;82(3):426–32.

[22] Halpern BS. The impact of marine reserves: Do reserves work and does reserve size matter? Ecol Appl. 2003 2;13(1):S117–S37.

[23] Bergstrom L, Kautsky L, Malm T, Rosenberg R, Wahlberg M, Capetillo NA, et al Effects of offshore wind farms on marine wildlife-a generalized impact assessment. Environmental Research Letters. 2014 3;9(3).

[24] Bergström L, Lagenfelt I, Sundqvist F, Andersson I, Andersson MH, Sigray P. Fiskundersökningar vid Lillgrund vindkraftpark. Slutredovisning av kontrollprogram för fisk och fiske 2002–2010. På uppdrag av Vattenfall Vindkraft AB. Havs och Vattenmyndigheten2013. Report No.: 2013:18.

[25] Reid DG, Abello P, Warman CG, Naylor E. SIZE-RELATED MATING SUCCESS IN THE SHORE CRAB CARCINUS-MAENAS (CRUSTACEA, BRACHYURA). Journal of Zoology. 1994 3;232:397–407.

[26] McGaw IJ, Naylor E. DISTRIBUTION AND RHYTHMIC LOCOMOTOR PATTERNS OF ESTUARINE AND OPEN-SHORE POPULATIONS OF CARCINUS-MAENAS. Journal of the Marine Biological Association of the United Kingdom. 1992 8;72(3):599–609.

[27] Ledesma FM, Van der Molen S, Baron PJ. Sex identification of Carcinus maenas by analysis of carapace geometrical morphometry. Journal of Sea Research. 2010 Apr-May;63(3–4):213–6.

[28] McGaw IJ, Naylor E. SALINITY PREFERENCE OF THE SHORE CRAB CARCINUS-MAENAS IN RELATION TO COLORATION DURING INTERMOLT AND TO PRIOR ACCLIMATION. Journal of Experimental Marine Biology and Ecology. 1992 1992;155(2):145–59.

[29] Warman CG, Reid DG, Naylor E. VARIATION IN THE TIDAL MIGRATORY BEHAVIOR AND RHYTHMIC LIGHT-RESPONSIVENESS IN THE SHORE CRAB, CARCINUS-MAENAS. Journal of the Marine Biological Association of the United Kingdom. 1993 5;73(2):355–64.

[30] Abello P, Aagaard A, Warman CG, Depledge MH. Spatial variability in the population structure of the shore crab Carcinus maenas (Crustacea: Brachyura) in a shallow-water, weakly tidal fjord. Marine Ecology Progress Series. 1997 2;147(1–3):97–103.

[31] Reid DG, Abello P, Kaiser MJ, Warman CG. Carapace colour, inter-moult duration and the behavioural and physiological ecology of the shore crab Carcinus maenas. Estuarine Coastal and Shelf Science. 1997 2;44(2):203–11.

[32] Amaral V, Cabral HN, Jenkins S, Hawkins S, Paula J. Comparing quality of estuarine and nearshore intertidal habitats for Carcinus maenas. Estuarine Coastal and Shelf Science. 2009 6 20;83(2):219–26.

[33] Hammar L, Wikström A, Börjesson P, Rosenberg R. Studier på småfisk vid Lillgrund vindpark—Effektstudier under konstruktionsarbeten och anläggning av gravitasjonsfundament Sweden: Naturvårdsverket 2008.

[34] Lindkvist T, Loindrow H, Hellström S. Årsrapport Hydrologi Öresunds Kustvattenkrontroll. Öresunds vattenvårdsförbund.2008.

[35] Jeppsson J, Larsen PE, Larsson Å. Technical Description Lillgrund Wind Power Plant, Vattenfall Vindkraft AB2008.

[36] Flodérus A. Experiences from the Construction and Installation of Lillgrund Wind Farm Lillgrund Pilot Project. The Swedish Energy Agency 2008.

[37] Crothers JB. Biology of the shore crab Carcinus maneas (L.). The life of the adult crab. Field Studies. 1968;2(5):579–614.

[38] Baeta A, Cabral HN, Neto JM, Marques JC, Pardal MA. Biology, population dynamics and secondary production of the green crab Carcinus maenas (L.) in a temperate estuary. Estuarine Coastal and Shelf Science. 2005 10;65(1–2):43–52.

[39] Cohen AN, Carlton JT, Fountain MC. INTRODUCTION, DISPERSAL AND POTENTIAL IMPACTS OF THE GREEN CRAB CARCINUS-MAENAS IN SAN-FRANCISCO BAY, CALIFORNIA. Marine Biology. 1995 4;122(2):225–37.

[40] Lowe S, Browne M, Boudjelas S, Poorter MD. 100 of the World’s Worst Invasive Alien Species A selection from the Global Invasive Species Database: Published by The Invasive Species Specialist Group (ISSG), a specialist group of the Species Survival Commission (SSC) of the World Conservation Union (IUCN); 2000.

[41] Moksnes P-O. The relative importance of habitat-specific settlement, predation and juvenile dispersal for distribution and abundance of young juvenile shore crabs Carcinus maenas L. J Exp Mar Biol Ecol. 2002;271(1):41–73.

[42] Moen FE, Svensen E. Marine fish & invertebrates of Northern Europe Kristiansund: KOM; 2004.

[43] Bergström L, Sundqvist F, Bergström U. Effekter av en havsbaserad vindkraftpark på fördelningen av bottennära fisk. Rapport 6485. Naturvårdsverket. Stockholm2012.

[44] Bergström L, Sundqvist F, Bergström U. Effects of an offshore wind farm on temporal and spatial patterns in the demersal fish community. Marine Ecology Progress Series. 2013;485:199–210.

[45] Ungfors A, Hallback H, Nilsson PG. Movement of adult edible crab (Cancer pagurus L.) at the Swedish West Coast by mark-recapture and acoustic tracking. Fisheries Research. 2007 5;84(3):345–57.

[46] FLOYTAG. http://www.floytag.com/uploads/floycatalog.pdf [cited 2016 18.03.2016].

[47] Munch-Petersen S, Sparre P, Hoffman E. Abundance of the shore crab, Carcinus maenas (L.), estimated from mark-recapture experiments. Dana. 1982:97–121.

[48] Haggarty DR, King JR. CPUE as an index of relative abundance for nearshore reef fishes. Fisheries Research. 2006;81(1):89–93.

[49] White GC, Burnham KP. Program MARK: survival estimation from populations of marked animals. Bird Study. 1999 1999;46:120–39.

[50] Murray LG, Seed R. Determining whether catch per unit effort is a suitable proxy for relative crab abundance. Marine Ecology Progress Series. 2010 2010;401:173–82.

[51] Sheehan EV, Thompson RC, Coleman RA, Attrill MJ. Positive feedback fishery: Population consequences of ‘crab-tiling’ on the green crab Carcinus maenas. Journal of Sea Research. 2008;60(4):303–9.

[52] Goransson P. Petersen's benthic macrofauna stations revisited in the Oresund area (southern Sweden) and species composition in the 1990s—signs of decreased biological variation. Sarsia. 2002 11 7;87(4):263–80.

[53] Enderlein P, Wahl M. Dominance of blue mussels versus consumer-mediated enhancement of benthic diversity. Journal of Sea Research. 2004;51(2):145–55.

[54] Flach EC. The separate and combined effects of epibenthic predation and presence of macro-infauna on the recruitment success of bivalves in shallow soft-bottom areas on the Swedish west coast. Journal of Sea Research. 2003;49(1):59–67.

[55] Kemppainen P, Nes S, Ceder C, Johannesson K. Refuge function of marine algae complicates selection in an intertidal snail. Oecologia. 2005 2005/04/01;143(3):402–11. doi: 10.1007/s00442-004-1819-5 15711994 10.1007/s00442-004-1819-5

[56] Pihl L, Rosenberg R. Production, Abundance, and Biomass of Mobile Epibenthic Marine Fauna in Shallow Waters, Western Sweden. J Exp Mar Biol Ecol. [Article]. 1982;57(2–3):273–301.

[57] Gomes V. First results of tagging experiments on crab Carcinus maenas (L.) in the Ria de Aveiro Lagoon Portugal. Ciencia Biologica Ecology and Systematics. 1991;11(1–2):21–30.

[58] Yamada SB, Dumbauld BR, Kalin A, Hunt CE, Figlar-Barnes R, Randall A. Growth and persistence of a recent invader Carcinus maenas in estuaries of the northeastern Pacific. Biol Invasions. 2005 3;7(2):309–21.

[59] McPherson R. Assessment of T bar anchor tags for marking the Blue Swimmer Crab Portunus pelagicus (L.). Fisheries Research. 2002;54(2):209–16.

[60] Kangas MI. Synopsis of the biology and exploitation of the blue swimmer crab, Portunus pelagicus Linnaeus, in Western Australia. Fish. Res. Rep. Fish. West Aust., 121: 1–22.2000.

[61] Janke K. Biological interactions and their role in community structure in the rocky intertidal of Helgoland (German Bight, North Sea). Helgolander Meeresunters. 1990 1990/06/01;44(2):219–63.

[62] Wale MA, Simpson SD, Radford AN. Size-dependent physiological responses of shore crabs to single and repeated playback of ship noise. Biology Letters. 2013 4 23;9(2). 10.1098/rsbl.2012.1194 PMC3639773 23445945

[63] Havsmiljöinstitutet. Sjöfarten kring Sverige och dess påverkan på havsmiljön. Göteborg2014.

[64] Reid D, Abello P, Warman C, Naylor E. Size related mating success in the shore crab Carcinus maenas (Crustacea: Brachyura). Journal of Zoology. 2009;232:397–407.

[65] Hansen KS. Small scale distribution of fish in offshore wind farms. Copenhagen: DTU AQUA 2012.

[66] Bamber SD, Naylor E. Sites of release of putative sex pheromone and sexual behaviour in female Carcinus maenas (Crustacea: Decapoda). Estuarine Coastal and Shelf Science. 1997 2;44(2):195–202.

[67] Ropes JW. FEEDING HABITS OF GREEN CRAB, CARCINUS-MAENAS (L). United States Fish and Wildlife Service Fishery Bulletin. 1969 1969;67(2):183-&.

