# Supplementary material for: Effects of an Offshore Wind Farm (OWF) on the Common Shore Crab Carcinus maenas: Tagging Pilot Experiments in the Lillgrund Offshore Wind Farm (Sweden)
Source: PLoS One. 2016 Oct 25;11(10):e0165096. doi: 10.1371/journal.pone.0165096 (PMC5079560; doi:10.1371/journal.pone.0165096)
Supplement: S1 Table — (PDF) [file pone.0165096.s001.pdf]

S1 Table. Dataset of captured *Carcinus maenas* , including ID, Year, Site, Station, Carapace Width, Sex, Color and Body Condition

ID	Year	Site	Station	CW (mm)	Sex	Color	Condition
1	2011	Sjollen	S9	63,71	male	green	good
2	2011	Sjollen	S9	53,78	male	green	reduced
3	2011	Sjollen	S9	61,67	male	green	good
4	2011	Sjollen	S9	54,27	male	green	good
5	2011	Sjollen	S9	62,40	male	green	good
6	2011	Sjollen	S9	49,05	male	green	good
7	2011	Sjollen	S9	47,59	male	green	good
8	2011	Sjollen	S9	44,73	male	green	good
9	2011	Sjollen	S9	49,64	male	green	good
10	2011	Sjollen	S9	48,45	male	green	good
11	2011	Sjollen	S9	48,60	male	green	good
12	2011	Sjollen	S9	48,13	male	green	good
13	2011	Sjollen	S7	66,80	male	green	good
14	2011	Sjollen	S7	61,85	male	green	good
15	2011	Sjollen	S7	48,46	male	green	good
16	2011	Sjollen	S7	47,92	male	green	good
17	2011	Sjollen	S7	60,92	male	green	reduced
18	2011	Sjollen	S7	53,15	male	green	good
19	2011	Sjollen	S7	45,02	male	green	good
20	2011	Sjollen	S7	48,25	male	green	good
21	2011	Sjollen	S7	54,63	male	green	good
22	2011	Sjollen	S7	57,06	male	green	good
23	2011	Sjollen	S7	50,94	male	green	good
24	2011	Sjollen	S7	52,87	male	green	good
25	2011	Sjollen	S7	48,65	male	green	good
26	2011	Sjollen	S4	61,62	male	green	good
27	2011	Sjollen	S4	49,72	male	green	good
28	2011	Sjollen	S4	71,50	male	green	good
29	2011	Sjollen	S4	52,56	male	green	good
30	2011	Sjollen	S4	58,90	female	green	good
31	2011	Sjollen	S4	61,06	male	green	good
32	2011	Sjollen	S4	55,14	male	green	good
33	2011	Sjollen	S4	58,60	male	green	good
34	2011	Sjollen	S4	51,82	male	green	good
35	2011	Sjollen	S4	47,18	male	green	reduced
36	2011	Sjollen	S4	60,59	male	green	good
37	2011	Sjollen	S4	48,11	male	green	good
38	2011	Sjollen	S4	31,95	male	green	good
39	2011	Sjollen	S4	58,30	male	green	good
40	2011	Sjollen	S2	57,66	male	green	good
41	2011	Sjollen	S2	52,93	male	red	good
42	2011	Sjollen	S2	59,43	male	green	good
43	2011	Sjollen	S2	49,96	male	green	good
44	2011	Sjollen	S2	59,44	male	green	good
45	2011	Sjollen	S2	61,04	male	green	good
46	2011	Sjollen	S2	63,38	male	green	good
47	2011	Sjollen	S2	62,54	male	green	good
48	2011	Sjollen	S2	56,35	male	green	good
49	2011	Sjollen	S2	69,46	male	green	good
50	2011	Sjollen	S2	53,68	male	green	reduced
51	2011	Sjollen	S2	59,13	male	green	good
52	2011	Sjollen	S2	56,97	male	green	good
53	2011	Sjollen	S2	45,13	female	green	good
54	2011	Sjollen	S2	57,62	male	green	reduced
55	2011	Sjollen	S2	66,54	male	green	good

56	2011	Sjollen	S2	60,54	male	green	good
57	2011	Sjollen	S2	48,76	male	green	good
58	2011	Sjollen	S2	48,59	male	green	good
59	2011	Sjollen	S2	40,14	male	green	good
60	2011	Sjollen	S6	61,93	male	red	good
61	2011	Sjollen	S6	56,49	male	red	reduced
62	2011	Sjollen	S6	71,81	male	green	reduced
63	2011	Sjollen	S6	61,58	male	green	good
64	2011	Sjollen	S6	60,93	male	green	good
65	2011	Sjollen	S6	66,25	male	green	good
66	2011	Sjollen	S6	57,21	male	green	good
67	2011	Sjollen	S6	64,02	male	green	good
68	2011	Sjollen	S6	62,41	male	green	good
69	2011	Sjollen	S6	60,88	male	green	good
70	2011	Sjollen	S6	58,62	male	green	good
71	2011	Sjollen	S6	63,53	male	green	good
72	2011	Sjollen	S6	61,03	male	green	good
73	2011	Sjollen	S6	63,88	male	green	reduced
74	2011	Sjollen	S6	52,20	male	green	good
75	2011	Sjollen	S6	62,32	male	green	good
76	2011	Sjollen	S6	56,14	male	green	good
77	2011	Sjollen	S6	61,17	male	green	good
78	2011	Sjollen	S6	57,94	male	green	reduced
79	2011	Sjollen	S6	57,97	male	green	good
80	2011	Sjollen	S6	62,32	male	green	good
81	2011	Sjollen	S6	53,31	male	green	good
82	2011	Sjollen	S6	55,78	male	green	good
83	2011	Sjollen	S6	50,80	male	green	good
84	2011	Sjollen	S6	53,86	male	green	good
85	2011	Sjollen	S6	51,92	male	green	good
86	2011	Sjollen	S6	57,87	male	green	good
87	2011	Sjollen	S6	54,65	male	green	good
88	2011	Sjollen	S6	55,26	male	green	good
89	2011	Sjollen	S6	49,83	male	green	good
90	2011	Sjollen	S6	46,85	female	red	good
91	2011	Sjollen	S6	44,98	male	green	good
92	2011	Sjollen	S6	46,83	male	green	good
93	2011	Sjollen	S6	45,63	male	green	good
94	2011	Sjollen	S6	46,11	female	red	good
95	2011	Sjollen	S6	44,66	female	red	good
96	2011	Sjollen	S6	46,83	male	green	good
97	2011	Sjollen	S6	45,41	male	green	good
98	2011	Sjollen	S6	42,73	female	red	good
99	2011	Sjollen	S5	67,45	male	green	good
100	2011	Sjollen	S5	62,87	male	green	good
101	2011	Sjollen	S5	61,54	male	green	good
102	2011	Sjollen	S5	62,26	male	green	good
103	2011	Sjollen	S5	67,48	male	green	good
104	2011	Sjollen	S5	66,94	male	green	good
105	2011	Sjollen	S5	65,95	male	green	good
106	2011	Sjollen	S5	60,13	male	green	good
107	2011	Sjollen	S5	58,95	male	red	good
108	2011	Sjollen	S5	61,58	male	green	good
109	2011	Sjollen	S5	65,45	male	green	good
110	2011	Sjollen	S5	57,98	male	green	good
111	2011	Sjollen	S5	57,45	male	green	good
112	2011	Sjollen	S5	55,74	male	green	good
113	2011	Sjollen	S5	57,48	male	green	good
114	2011	Sjollen	S5	54,97	male	green	good

115	2011	Sjollen	S5	59,51	male	green	good
116	2011	Sjollen	S5	57,21	male	green	good
117	2011	Sjollen	S5	52,43	male	green	good
118	2011	Sjollen	S5	55,29	male	green	good
119	2011	Sjollen	S5	23,46	male	green	good
120	2011	Sjollen	S5	51,00	male	green	good
121	2011	Sjollen	S5	43,93	male	green	good
122	2011	Sjollen	S5	46,76	male	green	good
123	2011	Sjollen	S5	49,20	male	green	good
124	2011	Sjollen	S3	70,07	male	green	good
125	2011	Sjollen	S3	71,14	male	green	good
126	2011	Sjollen	S3	72,63	male	green	good
127	2011	Sjollen	S3	63,22	male	green	good
128	2011	Sjollen	S3	63,56	male	green	good
129	2011	Sjollen	S3	62,71	male	green	good
130	2011	Sjollen	S3	56,35	male	green	good
131	2011	Sjollen	S3	57,35	male	green	good
132	2011	Sjollen	S3	61,18	male	green	good
133	2011	Sjollen	S3	63,23	male	green	good
134	2011	Sjollen	S3	65,65	male	green	good
135	2011	Sjollen	S3	56,00	male	green	good
136	2011	Sjollen	S3	60,76	male	green	good
137	2011	Sjollen	S3	60,00	male	green	good
138	2011	Sjollen	S3	61,06	male	green	good
139	2011	Sjollen	S3	49,77	male	green	good
140	2011	Sjollen	S3	57,02	male	green	good
141	2011	Sjollen	S3	54,79	male	green	good
142	2011	Sjollen	S3	57,09	male	green	good
143	2011	Sjollen	S3	48,33	male	green	good
144	2011	Sjollen	S3	47,21	female	red	good
145	2011	Sjollen	S3	49,42	male	green	good
146	2011	Sjollen	S3	49,42	male	green	good
147	2011	Sjollen	S3	43,90	male	green	good
148	2011	Sjollen	S3	42,28	male	red	good
149	2011	Sjollen	S10	63,57	male	green	good
150	2011	Sjollen	S10	65,27	male	green	good
151	2011	Sjollen	S10	67,61	male	green	good
152	2011	Sjollen	S10	66,23	male	green	good
153	2011	Sjollen	S10	60,09	male	green	good
154	2011	Sjollen	S10	62,39	male	green	good
155	2011	Sjollen	S10	62,39	male	green	good
156	2011	Sjollen	S10	60,56	male	green	good
157	2011	Sjollen	S10	60,97	male	green	good
158	2011	Sjollen	S10	60,07	male	green	good
159	2011	Sjollen	S10	53,40	male	green	good
160	2011	Sjollen	S10	55,14	male	green	good
161	2011	Sjollen	S10	62,08	male	green	good
162	2011	Sjollen	S10	63,13	male	green	good
163	2011	Sjollen	S10	66,31	male	green	good
164	2011	Sjollen	S10	50,93	male	green	good
165	2011	Sjollen	S10	50,32	male	green	good
166	2011	Sjollen	S10	61,65	male	green	good
167	2011	Sjollen	S10	62,62	male	green	good
168	2011	Sjollen	S10	44,97	male	green	good
169	2011	Sjollen	S10	59,97	male	green	good
170	2011	Sjollen	S10	56,81	male	red	good
171	2011	Sjollen	S10	52,06	male	green	good
172	2011	Sjollen	S10	45,63	male	green	good
173	2011	Sjollen	S10	47,63	male	green	good

174	2011	Sjollen	S10	46,43	male	red	good
175	2011	Sjollen	S10	47,76	male	green	good
176	2011	Sjollen	S10	40,84	female	green	good
177	2011	Sjollen	S10	43,87	male	green	good
178	2011	Sjollen	S10	37,16	female	green	good
179	2011	Sjollen	S8	54,78	male	red	good
180	2011	Sjollen	S8	61,78	male	green	good
181	2011	Sjollen	S8	59,95	male	green	good
182	2011	Sjollen	S8	67,50	male	green	good
183	2011	Sjollen	S8	64,19	male	green	good
184	2011	Sjollen	S8	61,36	male	green	good
185	2011	Sjollen	S8	57,22	male	green	good
186	2011	Sjollen	S8	66,49	male	green	good
187	2011	Sjollen	S8	53,92	male	green	good
188	2011	Sjollen	S8	63,69	male	green	good
189	2011	Sjollen	S8	63,69	male	green	good
190	2011	Sjollen	S8	66,73	male	green	good
191	2011	Sjollen	S8	61,36	male	green	good
192	2011	Sjollen	S8	49,83	male	green	good
193	2011	Sjollen	S8	55,50	male	green	good
194	2011	Sjollen	S8	50,40	male	green	good
195	2011	Sjollen	S8	52,47	male	green	good
196	2011	Sjollen	S8	52,50	female	red	good
197	2011	Sjollen	S8	45,50	female	red	good
198	2011	Sjollen	S8	48,60	male	red	good
199	2011	Sjollen	S8	52,80	male	green	good
200	2011	Sjollen	S8	51,60	male	green	good
201	2011	Sjollen	S1	61,20	male	green	good
202	2011	Sjollen	S1	62,17	male	green	good
203	2011	Sjollen	S1	62,12	male	green	good
204	2011	Sjollen	S1	60,80	male	green	good
205	2011	Sjollen	S1	50,00	male	green	good
206	2011	Sjollen	S1	58,40	male	green	good
207	2011	Sjollen	S1	56,70	male	green	good
208	2011	Sjollen	S1	58,20	male	green	good
209	2011	Sjollen	S1	60,20	male	green	good
210	2011	Sjollen	S1	68,00	male	green	good
211	2011	Sjollen	S1	66,50	male	green	good
212	2011	Sjollen	S1	57,00	male	green	good
213	2011	Sjollen	S1	60,40	male	green	good
214	2011	Sjollen	S1	54,10	male	green	good
215	2011	Sjollen	S1	57,30	male	green	good
216	2011	Sjollen	S1	48,50	male	green	good
217	2011	Sjollen	S1	51,90	male	green	good
218	2011	Sjollen	S1	54,40	male	green	good
219	2011	Sjollen	S1	50,30	male	green	good
220	2011	Sjollen	S1	52,40	male	green	good
221	2011	Sjollen	S1	57,30	male	green	good
222	2011	Sjollen	S1	58,10	male	green	good
223	2011	Sjollen	S1	52,80	male	green	good
224	2011	Sjollen	S1	52,90	male	green	good
225	2011	Sjollen	S1	50,60	male	green	good
226	2011	Sjollen	S1	55,50	male	green	good
227	2011	Sjollen	S1	52,30	male	green	good
228	2011	Sjollen	S1	47,10	male	green	good
229	2011	Sjollen	S1	50,00	male	green	good
230	2011	Sjollen	S1	52,10	male	green	good
231	2011	Sjollen	S1	51,10	male	green	good
232	2011	Sjollen	S1	50,30	male	green	good

233	2011	Sjollen	S1	51,50	male	green	good
234	2011	Sjollen	S1	49,30	male	green	good
235	2011	Sjollen	S1	46,30	male	green	good
236	2011	Sjollen	S1	40,80	male	green	good
237	2011	Sjollen	S1	47,10	male	green	good
238	2011	Sjollen	S1	47,10	male	green	good
239	2011	Sjollen	S9	66,90	male	green	good
240	2011	Sjollen	S9	57,30	male	green	good
241	2011	Sjollen	S9	68,66	male	green	good
242	2011	Sjollen	S9	59,20	male	green	good
243	2011	Sjollen	S9	51,70	male	green	good
244	2011	Sjollen	S9	57,00	male	green	good
245	2011	Sjollen	S9	58,80	male	green	good
246	2011	Sjollen	S9	58,80	male	green	good
247	2011	Sjollen	S9	62,50	male	green	good
248	2011	Sjollen	S9	59,40	male	green	good
249	2011	Sjollen	S9	62,70	male	green	good
250	2011	Sjollen	S9	49,10	male	green	good
251	2011	Sjollen	S9	48,40	male	green	good
252	2011	Sjollen	S9	56,80	male	green	good
253	2011	Sjollen	S9	63,80	male	green	good
254	2011	Sjollen	S9	59,00	male	green	good
255	2011	Sjollen	S9	48,70	male	green	good
256	2011	Sjollen	S9	57,50	male	green	good
257	2011	Sjollen	S9	60,80	male	green	good
258	2011	Sjollen	S9	54,10	male	green	good
259	2011	Sjollen	S9	56,20	male	red	good
260	2011	Sjollen	S9	63,61	male	green	good
261	2011	Sjollen	S9	61,47	male	green	good
262	2011	Sjollen	S9	55,90	male	green	good
263	2011	Sjollen	S9	56,20	male	green	good
264	2011	Sjollen	S9	55,70	male	green	good
265	2011	Sjollen	S9	50,00	male	green	good
266	2011	Sjollen	S9	62,21	female	green	good
267	2011	Sjollen	S9	44,10	male	green	good
268	2011	Sjollen	S1	64,30	male	green	good
269	2011	Sjollen	S1	50,90	male	green	good
270	2011	Sjollen	S1	66,40	male	green	good
271	2011	Sjollen	S1	64,40	male	red	good
272	2011	Sjollen	S1	59,00	male	green	good
273	2011	Sjollen	S1	60,20	male	green	good
274	2011	Sjollen	S1	60,70	male	green	good
275	2011	Sjollen	S1	59,90	male	green	good
276	2011	Sjollen	S1	58,80	male	green	good
277	2011	Sjollen	S1	59,40	male	green	good
278	2011	Sjollen	S1	53,60	male	green	good
279	2011	Sjollen	S1	63,00	male	green	good
280	2011	Sjollen	S1	52,80	male	green	good
281	2011	Sjollen	S1	63,40	male	green	good
282	2011	Sjollen	S1	52,70	male	green	good
283	2011	Sjollen	S1	56,40	male	green	good
284	2011	Sjollen	S1	51,40	male	green	good
285	2011	Sjollen	S1	58,70	male	green	good
286	2011	Sjollen	S1	58,70	male	green	good
287	2011	Sjollen	S1	63,80	male	green	good
288	2011	Sjollen	S1	56,40	male	green	good
289	2011	Sjollen	S1	56,30	male	green	good
290	2011	Sjollen	S1	56,50	male	green	good
291	2011	Sjollen	S1	60,40	male	green	good

292	2011	Sjollen	S1	67,40	male	green	good
293	2011	Sjollen	S1	68,90	male	green	good
294	2011	Sjollen	S1	49,00	male	green	good
295	2011	Sjollen	S1	60,50	male	green	good
296	2011	Sjollen	S1	57,10	male	green	good
297	2011	Sjollen	S1	72,70	male	green	good
298	2011	Sjollen	S1	53,70	male	green	good
299	2011	Sjollen	S1	55,50	male	green	good
300	2011	Sjollen	S1	52,70	male	green	good
301	2011	Sjollen	S1	54,80	male	green	good
302	2011	Sjollen	S1	63,20	male	green	good
303	2011	Sjollen	S1	54,50	male	green	good
304	2011	Sjollen	S1	63,30	male	green	good
305	2011	Sjollen	S1	48,20	male	green	good
306	2011	Sjollen	S1	49,70	male	green	good
307	2011	Sjollen	S1	46,10	male	green	good
308	2011	Sjollen	S1	59,30	male	green	good
309	2011	Sjollen	S1	55,20	male	green	good
310	2011	Sjollen	S1	52,70	male	green	good
311	2011	Sjollen	S1	46,30	male	green	good
312	2011	Sjollen	S1	55,80	male	green	good
313	2011	Sjollen	S1	50,50	male	green	good
314	2011	Sjollen	S1	55,50	male	green	good
315	2011	Sjollen	S1	50,90	male	green	good
316	2011	Sjollen	S1	57,40	male	green	good
317	2011	Sjollen	S1	64,10	male	green	good
318	2011	Sjollen	S1	49,20	male	green	good
319	2011	Sjollen	S1	47,70	male	green	good
320	2011	Sjollen	S1	64,40	male	green	good
321	2011	Sjollen	S1	49,60	male	green	good
322	2011	Sjollen	S1	42,70	male	green	good
323	2011	Sjollen	S1	48,00	male	green	good
324	2011	Sjollen	S1	48,40	male	green	good
325	2011	Sjollen	S1	48,90	female	red	good
326	2011	Sjollen	S1	52,00	male	green	good
327	2011	Sjollen	S1	33,10	female	green	good
328	2011	Sjollen	S1	48,40	male	green	good
329	2011	Sjollen	S1	52,20	male	green	good
330	2011	Sjollen	S1	43,10	female	green	good
331	2011	Sjollen	S1	41,10	male	green	good
332	2011	Sjollen	S8	58,60	male	green	good
333	2011	Sjollen	S8	65,20	male	green	good
334	2011	Sjollen	S8	65,30	male	green	good
335	2011	Sjollen	S8	57,00	male	green	good
336	2011	Sjollen	S8	52,10	male	green	good
337	2011	Sjollen	S8	67,40	male	green	good
338	2011	Sjollen	S8	63,70	male	green	good
339	2011	Sjollen	S8	63,40	male	green	good
340	2011	Sjollen	S8	57,80	male	green	good
341	2011	Sjollen	S8	57,80	male	green	good
342	2011	Sjollen	S8	56,52	male	green	good
343	2011	Sjollen	S8	63,50	male	green	good
344	2011	Sjollen	S8	56,70	male	green	good
345	2011	Sjollen	S8	68,20	male	green	good
346	2011	Sjollen	S8	59,80	male	green	good
347	2011	Sjollen	S8	55,00	male	green	good
348	2011	Sjollen	S8	59,70	male	green	good
349	2011	Sjollen	S8	56,10	male	green	good
350	2011	Sjollen	S8	58,00	male	green	good

351	2011	Sjollen	S8	60,60	male	green	good
352	2011	Sjollen	S8	64,80	male	green	good
353	2011	Sjollen	S8	65,70	male	green	good
354	2011	Sjollen	S8	62,00	male	green	good
355	2011	Sjollen	S8	58,10	male	green	good
356	2011	Sjollen	S8	55,70	male	green	good
357	2011	Sjollen	S8	55,60	male	green	good
358	2011	Sjollen	S8	57,80	male	green	good
359	2011	Sjollen	S8	58,20	male	green	good
360	2011	Sjollen	S8	65,00	male	green	good
361	2011	Sjollen	S8	53,40	male	green	good
362	2011	Sjollen	S8	49,80	male	green	good
363	2011	Sjollen	S8	53,60	male	green	good
364	2011	Sjollen	S8	52,50	male	green	good
365	2011	Sjollen	S8	45,60	male	green	good
366	2011	Sjollen	S8	49,50	male	green	good
367	2011	Sjollen	S8	47,80	male	green	good
368	2011	Sjollen	S8	46,80	male	green	good
369	2011	Sjollen	S8	45,70	male	green	good
370	2011	Sjollen	S8	47,20	male	green	good
371	2011	Sjollen	S8	43,70	male	green	good
372	2011	Sjollen	S8	41,50	male	green	good
373	2011	Sjollen	S10	64,90	male	green	good
374	2011	Sjollen	S10	60,70	male	red	good
375	2011	Sjollen	S10	61,30	male	green	good
376	2011	Sjollen	S10	59,50	male	green	good
377	2011	Sjollen	S10	57,70	male	green	good
378	2011	Sjollen	S10	60,40	male	green	good
379	2011	Sjollen	S10	63,20	male	green	good
380	2011	Sjollen	S10	62,80	male	green	good
381	2011	Sjollen	S10	59,80	male	green	good
382	2011	Sjollen	S10	61,80	male	green	good
383	2011	Sjollen	S10	59,40	male	green	good
384	2011	Sjollen	S10	59,40	male	green	good
385	2011	Sjollen	S10	65,80	male	green	good
386	2011	Sjollen	S10	60,40	male	green	good
387	2011	Sjollen	S10	58,00	male	green	good
388	2011	Sjollen	S10	66,20	male	green	good
389	2011	Sjollen	S10	66,20	male	green	good
390	2011	Sjollen	S10	53,80	male	green	good
391	2011	Sjollen	S10	68,90	male	green	good
392	2011	Sjollen	S10	62,40	male	green	good
393	2011	Sjollen	S10	59,70	male	green	good
394	2011	Sjollen	S10	53,70	male	green	good
395	2011	Sjollen	S10	59,90	male	green	good
396	2011	Sjollen	S10	64,10	male	green	good
397	2011	Sjollen	S10	57,00	male	green	good
398	2011	Sjollen	S10	52,50	male	red	good
399	2011	Sjollen	S10	55,40	male	green	good
400	2011	Sjollen	S10	57,30	male	green	good
401	2011	Sjollen	S10	53,80	male	green	good
402	2011	Sjollen	S10	53,70	male	green	good
403	2011	Sjollen	S10	56,90	male	red	good
404	2011	Sjollen	S10	62,12	male	green	good
405	2011	Sjollen	S10	56,10	male	green	good
406	2011	Sjollen	S10	52,50	male	green	good
407	2011	Sjollen	S10	59,10	male	green	good
408	2011	Sjollen	S10	57,70	male	green	good
409	2011	Sjollen	S10	56,40	male	green	good

410	2011	Sjollen	S10	56,40	male	green	good
411	2011	Sjollen	S10	51,60	male	green	good
412	2011	Sjollen	S10	62,10	male	green	good
413	2011	Sjollen	S10	57,50	male	green	good
414	2011	Sjollen	S10	60,70	male	green	good
415	2011	Sjollen	S10	58,40	male	green	good
416	2011	Sjollen	S10	56,60	male	green	good
417	2011	Sjollen	S10	49,10	male	green	good
418	2011	Sjollen	S10	58,30	male	green	good
419	2011	Sjollen	S10	51,30	male	green	good
420	2011	Sjollen	S10	61,40	male	green	good
421	2011	Sjollen	S10	56,90	male	green	good
422	2011	Sjollen	S5	60,70	male	green	good
423	2011	Sjollen	S5	64,10	male	green	good
424	2011	Sjollen	S5	60,90	male	green	good
425	2011	Sjollen	S5	59,80	male	green	good
426	2011	Sjollen	S5	52,80	male	green	good
427	2011	Sjollen	S5	60,20	male	green	good
428	2011	Sjollen	S5	69,20	male	green	good
429	2011	Sjollen	S5	56,00	male	green	good
430	2011	Sjollen	S5	60,40	male	green	good
431	2011	Sjollen	S5	58,70	male	green	good
432	2011	Sjollen	S5	49,10	male	green	good
433	2011	Sjollen	S5	66,50	male	green	good
434	2011	Sjollen	S5	61,35	male	green	good
435	2011	Sjollen	S5	65,30	male	green	good
436	2011	Sjollen	S5	59,70	male	green	good
437	2011	Sjollen	S5	62,00	male	green	good
438	2011	Sjollen	S5	67,70	male	green	good
439	2011	Sjollen	S5	64,70	male	green	good
440	2011	Sjollen	S5	60,50	male	green	good
441	2011	Sjollen	S5	61,70	male	green	good
442	2011	Sjollen	S5	59,40	male	green	good
443	2011	Sjollen	S5	61,70	male	red	good
444	2011	Sjollen	S5	57,60	male	green	good
445	2011	Sjollen	S5	66,20	male	green	good
446	2011	Sjollen	S5	57,60	male	green	good
447	2011	Sjollen	S5	55,60	male	green	good
448	2011	Sjollen	S5	62,60	male	green	good
449	2011	Sjollen	S5	56,60	male	green	good
450	2011	Sjollen	S5	59,20	male	green	good
451	2011	Sjollen	S5	50,80	male	green	good
452	2011	Sjollen	S5	49,40	male	green	good
453	2011	Sjollen	S5	55,30	male	green	good
454	2011	Sjollen	S5	58,50	male	green	good
455	2011	Sjollen	S5	51,60	male	green	good
456	2011	Sjollen	S5	52,80	male	green	good
457	2011	Sjollen	S5	51,80	male	green	good
458	2011	Sjollen	S5	51,60	male	green	good
459	2011	Sjollen	S5	49,40	male	green	good
460	2011	Sjollen	S5	39,60	male	green	good
461	2011	Sjollen	S3	58,15	male	green	good
462	2011	Sjollen	S3	63,40	male	green	good
463	2011	Sjollen	S3	65,50	male	green	good
464	2011	Sjollen	S3	66,50	male	green	good
465	2011	Sjollen	S3	63,80	male	green	good
466	2011	Sjollen	S3	51,70	male	green	good
467	2011	Sjollen	S3	46,70	male	green	good
468	2011	Sjollen	S3	49,20	male	green	good

469	2011	Sjollen	S3	55,30	male	green	good
470	2011	Sjollen	S3	58,00	male	green	good
471	2011	Sjollen	S3	53,60	female	red	good
472	2011	Sjollen	S3	68,60	male	green	good
473	2011	Sjollen	S3	65,40	male	green	good
474	2011	Sjollen	S3	66,90	male	green	good
475	2011	Sjollen	S3	70,10	male	green	good
476	2011	Sjollen	S3	62,70	male	green	good
477	2011	Sjollen	S3	59,70	male	green	good
478	2011	Sjollen	S3	55,30	male	green	good
479	2011	Sjollen	S3	63,20	male	green	good
480	2011	Sjollen	S3	53,20	male	green	good
481	2011	Sjollen	S3	57,80	male	green	good
482	2011	Sjollen	S3	62,60	male	green	good
483	2011	Sjollen	S3	51,60	male	green	good
484	2011	Sjollen	S3	63,50	male	green	good
485	2011	Sjollen	S3	62,10	male	green	good
486	2011	Sjollen	S3	54,50	male	green	good
487	2011	Sjollen	S3	54,20	male	green	good
488	2011	Sjollen	S3	43,40	female	red	good
489	2011	Sjollen	S3	46,60	male	green	good
490	2011	Sjollen	S6	58,50	male	green	good
491	2011	Sjollen	S6	63,90	male	green	good
492	2011	Sjollen	S6	68,00	male	green	good
493	2011	Sjollen	S6	63,90	male	green	good
494	2011	Sjollen	S6	58,60	male	green	good
495	2011	Sjollen	S6	63,80	male	green	good
496	2011	Sjollen	S6	61,60	male	green	good
497	2011	Sjollen	S6	60,80	male	green	good
498	2011	Sjollen	S6	55,90	male	green	good
499	2011	Sjollen	S6	53,40	male	green	good
500	2011	Sjollen	S6	67,60	male	green	good
501	2011	Sjollen	S6	59,80	male	green	good
502	2011	Sjollen	S6	58,80	male	green	good
503	2011	Sjollen	S6	60,50	male	green	good
504	2011	Sjollen	S6	58,80	male	green	good
505	2011	Sjollen	S6	56,00	male	green	good
506	2011	Sjollen	S6	59,70	male	red	good
507	2011	Sjollen	S6	58,10	male	green	good
508	2011	Sjollen	S6	58,40	male	green	good
509	2011	Sjollen	S6	65,40	male	red	good
510	2011	Sjollen	S6	65,40	male	green	good
511	2011	Sjollen	S6	59,80	male	green	good
512	2011	Sjollen	S6	66,10	male	green	good
513	2011	Sjollen	S6	55,30	male	green	good
514	2011	Sjollen	S6	53,90	male	red	good
515	2011	Sjollen	S6	62,90	male	green	good
516	2011	Sjollen	S6	61,90	male	green	good
517	2011	Sjollen	S6	63,50	male	red	good
518	2011	Sjollen	S6	56,40	male	green	good
519	2011	Sjollen	S6	65,80	male	green	good
520	2011	Sjollen	S6	59,30	male	green	good
521	2011	Sjollen	S6	59,30	male	green	good
522	2011	Sjollen	S6	59,30	male	green	good
523	2011	Sjollen	S6	60,70	male	green	good
524	2011	Sjollen	S6	71,50	male	red	good
525	2011	Sjollen	S6	48,20	female	green	good
526	2011	Sjollen	S6	51,30	male	green	good
527	2011	Sjollen	S6	60,70	male	green	good

528	2011	Sjollen	S6	56,40	male	green	good
529	2011	Sjollen	S6	61,50	male	green	good
530	2011	Sjollen	S6	56,40	male	green	good
531	2011	Sjollen	S6	57,20	male	green	good
532	2011	Sjollen	S6	52,70	male	green	good
533	2011	Sjollen	S6	60,20	male	green	good
534	2011	Sjollen	S6	63,70	male	green	good
535	2011	Sjollen	S6	54,50	male	green	good
536	2011	Sjollen	S6	57,40	male	green	good
537	2011	Sjollen	S6	53,10	male	green	good
538	2011	Sjollen	S6	48,00	male	green	good
539	2011	Sjollen	S4	55,60	male	green	good
540	2011	Sjollen	S4	52,30	male	green	good
541	2011	Sjollen	S4	59,90	male	green	good
542	2011	Sjollen	S4	62,60	male	green	good
543	2011	Sjollen	S4	58,40	male	green	good
544	2011	Sjollen	S4	53,50	male	green	good
545	2011	Sjollen	S4	69,40	male	green	good
546	2011	Sjollen	S4	52,00	male	green	good
547	2011	Sjollen	S4	60,00	male	green	good
548	2011	Sjollen	S4	59,50	male	green	good
549	2011	Sjollen	S4	70,30	male	green	good
550	2011	Sjollen	S4	61,70	male	green	good
551	2011	Sjollen	S4	62,10	male	green	good
552	2011	Sjollen	S4	58,20	male	green	good
553	2011	Sjollen	S4	61,90	male	green	good
554	2011	Sjollen	S4	54,10	male	green	good
555	2011	Sjollen	S4	61,30	male	green	good
556	2011	Sjollen	S4	63,70	male	red	good
557	2011	Sjollen	S4	59,70	male	green	good
558	2011	Sjollen	S4	59,80	male	green	good
559	2011	Sjollen	S4	66,80	male	green	good
560	2011	Sjollen	S4	58,40	male	green	good
561	2011	Sjollen	S4	57,80	male	green	good
562	2011	Sjollen	S4	48,80	male	green	good
563	2011	Sjollen	S4	47,30	male	green	good
564	2011	Sjollen	S4	60,30	male	green	good
565	2011	Sjollen	S4	50,50	male	green	good
566	2011	Sjollen	S4	57,80	male	green	good
567	2011	Sjollen	S4	54,10	male	green	good
568	2011	Sjollen	S4	53,70	male	green	good
569	2011	Sjollen	S4	50,00	male	green	good
570	2011	Sjollen	S4	50,60	male	green	good
571	2011	Sjollen	S4	55,60	male	green	good
572	2011	Sjollen	S4	56,00	male	green	good
573	2011	Sjollen	S4	47,50	male	green	good
574	2011	Sjollen	S4	53,70	male	green	good
575	2011	Sjollen	S4	56,60	female	red	good
576	2011	Sjollen	S4	44,20	male	green	good
577	2011	Sjollen	S4	46,20	male	green	good
578	2011	Sjollen	S4	47,40	male	green	good
579	2011	Sjollen	S4	46,50	male	green	good
580	2011	Sjollen	S4	57,40	male	green	good
581	2011	Sjollen	S4	49,00	male	green	good
582	2011	Sjollen	S4	53,00	male	green	good
583	2011	Sjollen	S4	50,90	male	green	good
584	2011	Sjollen	S4	49,50	male	green	good
585	2011	Sjollen	S4	46,90	male	green	good
586	2011	Sjollen	S4	39,70	male	green	good

587	2011	Sjollen	S2	54,30	female	green	good
588	2011	Sjollen	S2	53,80	male	green	good
589	2011	Sjollen	S2	75,50	male	green	good
590	2011	Sjollen	S2	64,00	male	green	good
591	2011	Sjollen	S2	60,80	male	green	good
592	2011	Sjollen	S2	53,20	male	green	good
593	2011	Sjollen	S2	62,00	male	green	good
594	2011	Sjollen	S2	57,30	male	green	good
595	2011	Sjollen	S2	61,40	male	green	good
596	2011	Sjollen	S2	58,80	male	green	good
597	2011	Sjollen	S2	66,90	male	green	good
598	2011	Sjollen	S2	53,00	male	green	good
599	2011	Sjollen	S2	64,80	male	green	good
600	2011	Sjollen	S2	49,20	male	green	good
601	2011	Sjollen	S2	58,40	male	green	good
602	2011	Sjollen	S2	55,10	male	green	good
603	2011	Sjollen	S2	55,10	male	green	good
604	2011	Sjollen	S2	67,20	male	green	good
605	2011	Sjollen	S2	66,90	male	green	good
606	2011	Sjollen	S2	60,00	male	green	good
607	2011	Sjollen	S2	59,50	male	green	good
608	2011	Sjollen	S2	47,80	male	green	good
609	2011	Sjollen	S2	52,10	male	green	good
610	2011	Sjollen	S2	57,40	male	green	good
611	2011	Sjollen	S2	57,40	male	green	good
612	2011	Sjollen	S2	57,90	male	green	good
613	2011	Sjollen	S2	64,70	male	green	good
614	2011	Sjollen	S2	67,90	male	green	good
615	2011	Sjollen	S2	57,80	male	green	good
616	2011	Sjollen	S2	49,20	male	green	good
617	2011	Sjollen	S2	59,20	male	green	good
618	2011	Sjollen	S2	51,00	male	green	good
619	2011	Sjollen	S2	52,60	male	green	good
620	2011	Sjollen	S2	55,20	male	green	good
621	2011	Sjollen	S2	53,50	male	green	good
622	2011	Sjollen	S2	50,90	male	green	good
623	2011	Sjollen	S2	48,70	male	red	good
624	2011	Sjollen	S2	45,90	male	green	good
625	2011	Sjollen	S2	46,30	male	green	good
626	2011	Sjollen	S2	58,40	male	green	good
627	2011	Sjollen	S2	51,10	male	green	good
628	2011	Sjollen	S2	54,20	male	green	good
629	2011	Sjollen	S2	59,30	male	green	good
630	2011	Sjollen	S2	57,90	male	green	good
631	2011	Sjollen	S2	61,50	male	green	good
632	2011	Sjollen	S2	60,40	male	green	good
633	2011	Sjollen	S2	58,10	male	green	good
634	2011	Sjollen	S2	56,80	male	green	good
635	2011	Sjollen	S2	48,80	male	green	good
636	2011	Sjollen	S2	50,80	male	green	good
637	2011	Sjollen	S7	55,40	male	green	good
638	2011	Sjollen	S7	67,09	male	green	good
639	2011	Sjollen	S7	66,14	male	green	good
640	2011	Sjollen	S7	60,78	male	green	good
641	2011	Sjollen	S7	62,85	male	green	good
642	2011	Sjollen	S7	59,27	male	green	good
643	2011	Sjollen	S7	63,30	male	green	good
644	2011	Sjollen	S7	61,61	male	green	good
645	2011	Sjollen	S7	58,60	male	green	good

646	2011	Sjollen	S7	55,38	male	green	good
647	2011	Sjollen	S7	48,45	male	green	good
648	2011	Sjollen	S7	67,64	male	green	good
649	2011	Sjollen	S7	52,33	male	green	good
650	2011	Sjollen	S7	57,54	male	green	good
651	2011	Sjollen	S7	51,42	male	green	good
652	2011	Sjollen	S7	46,88	male	green	good
653	2011	Sjollen	S7	61,27	male	green	good
654	2011	Sjollen	S7	59,36	male	green	good
655	2011	Sjollen	S7	52,07	male	green	good
656	2011	Sjollen	S7	56,20	male	green	good
657	2011	Sjollen	S7	51,89	male	green	good
658	2011	Sjollen	S7	55,10	male	green	good
659	2011	Sjollen	S7	52,76	male	green	good
660	2011	Sjollen	S7	58,76	male	green	good
661	2011	Sjollen	S7	48,57	male	green	good
662	2011	Sjollen	S7	43,16	male	green	good
663	2011	Sjollen	S7	51,87	male	green	good
664	2011	Sjollen	S7	45,46	male	green	good
665	2011	Sjollen	S7	50,35	male	green	good
666	2011	Sjollen	S7	44,85	male	green	good
667	2011	Sjollen	S7	48,74	male	green	good
668	2011	Sjollen	S7	51,73	male	green	good
669	2011	Sjollen	S7	47,40	male	green	good
670	2011	Sjollen	S7	44,96	male	green	good
671	2011	Sjollen	S7	43,17	male	green	good
672	2011	Lillgrund	L6	68,06	male	green	good
673	2011	Lillgrund	L6	64,24	male	green	good
674	2011	Lillgrund	L6	61,81	male	green	good
675	2011	Lillgrund	L6	71,55	male	green	good
676	2011	Lillgrund	L6	56,35	male	green	good
677	2011	Lillgrund	L6	56,73	male	green	good
678	2011	Lillgrund	L6	73,18	male	green	good
679	2011	Lillgrund	L6	75,81	male	green	good
680	2011	Lillgrund	L6	30,70	male	green	good
681	2011	Lillgrund	L6	52,38	male	green	good
682	2011	Lillgrund	L6	59,32	male	green	good
683	2011	Lillgrund	L6	62,86	male	green	good
684	2011	Lillgrund	L6	63,74	male	green	good
685	2011	Lillgrund	L6	61,80	male	green	good
686	2011	Lillgrund	L6	59,15	male	green	good
687	2011	Lillgrund	L6	64,09	male	green	good
688	2011	Lillgrund	L6	60,76	male	green	good
689	2011	Lillgrund	L6	57,47	male	green	good
690	2011	Lillgrund	L6	50,98	male	green	good
691	2011	Lillgrund	L6	46,77	female	green	good
692	2011	Lillgrund	L7	63,31	male	green	good
693	2011	Lillgrund	L7	60,08	male	green	good
694	2011	Lillgrund	L7	63,25	male	green	good
695	2011	Lillgrund	L7	62,30	male	green	good
696	2011	Lillgrund	L7	63,77	male	green	good
697	2011	Lillgrund	L7	61,81	male	green	good
698	2011	Lillgrund	L7	63,03	male	green	good
699	2011	Lillgrund	L7	59,58	male	red	good
700	2011	Lillgrund	L7	56,57	male	red	good
701	2011	Lillgrund	L7	63,12	male	green	good
702	2011	Lillgrund	L7	56,63	male	green	good
703	2011	Lillgrund	L7	59,60	male	green	good
704	2011	Lillgrund	L7	57,65	male	green	good

705	2011	Lillgrund	L7	66,82	male	green	good
706	2011	Lillgrund	L7	67,44	male	green	good
707	2011	Lillgrund	L7	61,43	male	green	good
708	2011	Lillgrund	L7	58,81	male	green	good
709	2011	Lillgrund	L7	57,81	male	green	good
710	2011	Lillgrund	L7	57,81	male	green	good
711	2011	Lillgrund	L7	54,92	male	green	good
712	2011	Lillgrund	L7	54,92	male	green	good
713	2011	Lillgrund	L7	36,40	male	green	good
714	2011	Lillgrund	L7	53,13	male	green	good
715	2011	Lillgrund	L7	56,73	male	green	good
716	2011	Lillgrund	L7	58,33	male	green	good
717	2011	Lillgrund	L7	54,93	male	green	good
718	2011	Lillgrund	L7	52,15	male	green	good
719	2011	Lillgrund	L7	54,00	male	green	good
720	2011	Lillgrund	L7	48,36	male	green	good
721	2011	Lillgrund	L8	66,42	male	green	good
722	2011	Lillgrund	L8	67,28	male	green	good
723	2011	Lillgrund	L8	57,54	male	red	good
724	2011	Lillgrund	L8	58,98	male	green	good
725	2011	Lillgrund	L8	58,71	male	green	good
726	2011	Lillgrund	L8	54,37	male	green	good
727	2011	Lillgrund	L8	55,66	male	green	good
728	2011	Lillgrund	L8	55,10	male	green	good
729	2011	Lillgrund	L8	62,48	male	green	good
730	2011	Lillgrund	L8	60,42	male	green	good
731	2011	Lillgrund	L8	69,33	male	green	good
732	2011	Lillgrund	L8	57,25	male	green	good
733	2011	Lillgrund	L8	63,18	male	green	good
734	2011	Lillgrund	L8	63,59	male	green	good
735	2011	Lillgrund	L8	55,43	male	red	good
736	2011	Lillgrund	L8	57,95	male	green	good
737	2011	Lillgrund	L8	60,18	male	green	good
738	2011	Lillgrund	L8	56,99	male	green	good
739	2011	Lillgrund	L8	55,04	male	green	good
740	2011	Lillgrund	L8	50,86	male	green	good
741	2011	Lillgrund	L8	58,83	male	green	good
742	2011	Lillgrund	L8	55,26	male	green	good
743	2011	Lillgrund	L8	59,92	male	green	good
744	2011	Lillgrund	L8	52,96	male	green	good
745	2011	Lillgrund	L8	50,69	male	green	good
746	2011	Lillgrund	L8	52,95	male	green	good
747	2011	Lillgrund	L10	69,25	male	green	good
748	2011	Lillgrund	L10	62,42	male	red	good
749	2011	Lillgrund	L10	67,69	male	green	good
750	2011	Lillgrund	L10	68,18	male	green	good
751	2011	Lillgrund	L10	63,59	male	green	good
752	2011	Lillgrund	L10	56,54	male	red	good
753	2011	Lillgrund	L10	66,72	male	green	good
754	2011	Lillgrund	L10	59,99	male	green	good
755	2011	Lillgrund	L10	37,96	male	green	good
756	2011	Lillgrund	L10	58,19	male	green	good
757	2011	Lillgrund	L10	54,08	male	green	good
758	2011	Lillgrund	L10	60,18	male	green	good
759	2011	Lillgrund	L10	61,05	male	green	good
760	2011	Lillgrund	L10	57,54	male	green	good
761	2011	Lillgrund	L10	52,93	male	green	good
762	2011	Lillgrund	L10	55,05	male	green	good
763	2011	Lillgrund	L10	56,33	male	green	good

764	2011	Lillgrund	L10	50,52	female	red	good
765	2011	Lillgrund	L10	60,35	male	green	good
766	2011	Lillgrund	L10	45,32	male	green	good
767	2011	Lillgrund	L10	48,73	female	red	good
768	2011	Lillgrund	L9	42,26	female	red	good
769	2011	Lillgrund	L9	62,45	male	green	good
770	2011	Lillgrund	L9	63,33	male	green	good
771	2011	Lillgrund	L9	62,50	male	red	good
772	2011	Lillgrund	L9	59,47	male	green	good
773	2011	Lillgrund	L9	58,68	male	green	good
774	2011	Lillgrund	L9	53,44	male	green	good
775	2011	Lillgrund	L9	58,28	male	red	good
776	2011	Lillgrund	L9	56,41	male	green	good
777	2011	Lillgrund	L9	58,13	male	green	good
778	2011	Lillgrund	L9	63,33	male	green	good
779	2011	Lillgrund	L9	64,66	male	green	good
780	2011	Lillgrund	L9	60,12	male	green	good
781	2011	Lillgrund	L9	58,25	male	green	good
782	2011	Lillgrund	L9	65,79	male	green	good
783	2011	Lillgrund	L9	55,16	male	green	good
784	2011	Lillgrund	L9	53,78	male	red	good
785	2011	Lillgrund	L9	53,90	male	green	good
786	2011	Lillgrund	L9	54,00	male	green	good
787	2011	Lillgrund	L9	44,22	male	green	good
788	2011	Lillgrund	L3	62,99	male	green	good
789	2011	Lillgrund	L3	57,99	male	red	good
790	2011	Lillgrund	L3	65,41	male	green	good
791	2011	Lillgrund	L3	69,20	male	green	good
792	2011	Lillgrund	L3	70,10	male	green	good
793	2011	Lillgrund	L3	55,40	male	green	good
794	2011	Lillgrund	L3	60,21	male	green	good
795	2011	Lillgrund	L3	54,35	male	green	good
796	2011	Lillgrund	L3	61,41	male	green	good
797	2011	Lillgrund	L3	57,41	male	green	good
798	2011	Lillgrund	L3	54,67	male	green	good
799	2011	Lillgrund	L3	52,95	male	green	good
800	2011	Lillgrund	L3	54,26	male	green	good
801	2011	Lillgrund	L3	51,94	male	green	good
802	2011	Lillgrund	L3	57,55	male	green	good
803	2011	Lillgrund	L2	66,04	male	green	good
804	2011	Lillgrund	L2	60,96	male	green	good
805	2011	Lillgrund	L2	60,47	male	green	good
806	2011	Lillgrund	L2	61,14	male	green	good
807	2011	Lillgrund	L2	58,53	male	green	good
808	2011	Lillgrund	L2	60,20	male	green	good
809	2011	Lillgrund	L2	55,83	male	green	good
810	2011	Lillgrund	L2	65,00	male	green	good
811	2011	Lillgrund	L2	56,68	male	green	good
812	2011	Lillgrund	L2	55,20	male	green	good
813	2011	Lillgrund	L2	51,94	male	green	good
814	2011	Lillgrund	L2	64,07	male	green	good
815	2011	Lillgrund	L2	51,45	male	green	good
816	2011	Lillgrund	L2	65,84	male	green	good
817	2011	Lillgrund	L2	46,41	male	green	good
818	2011	Lillgrund	L2	63,14	male	green	good
819	2011	Lillgrund	L2	59,05	male	green	good
820	2011	Lillgrund	L2	58,41	male	green	good
821	2011	Lillgrund	L2	55,02	male	green	good
822	2011	Lillgrund	L2	54,85	male	green	good

823	2011	Lillgrund	L2	58,69	male	green	good
824	2011	Lillgrund	L2	52,39	male	green	good
825	2011	Lillgrund	L2	60,17	male	green	good
826	2011	Lillgrund	L2	56,96	male	green	good
827	2011	Lillgrund	L2	65,33	male	green	good
828	2011	Lillgrund	L2	57,37	male	green	good
829	2011	Lillgrund	L2	51,86	male	green	good
830	2011	Lillgrund	L2	63,73	male	green	good
831	2011	Lillgrund	L2	45,00	female	green	good
832	2011	Lillgrund	L2	51,14	male	green	good
833	2011	Lillgrund	L2	40,85	female	green	good
834	2011	Lillgrund	L2	42,33	female	green	good
835	2011	Lillgrund	L2	52,48	male	green	good
836	2011	Lillgrund	L2	49,40	male	green	good
837	2011	Lillgrund	L2	57,50	male	green	good
838	2011	Lillgrund	L2	49,80	male	green	good
839	2011	Lillgrund	L1	58,20	male	green	good
840	2011	Lillgrund	L1	63,43	male	green	good
841	2011	Lillgrund	L1	56,20	male	red	good
842	2011	Lillgrund	L1	59,11	male	green	good
843	2011	Lillgrund	L1	48,95	male	green	good
844	2011	Lillgrund	L1	63,86	male	green	good
845	2011	Lillgrund	L1	56,12	male	red	good
846	2011	Lillgrund	L1	55,13	male	green	good
847	2011	Lillgrund	L1	52,00	male	green	good
848	2011	Lillgrund	L1	54,20	male	green	good
849	2011	Lillgrund	L1	55,43	male	green	good
850	2011	Lillgrund	L1	59,40	male	green	good
851	2011	Lillgrund	L1	61,30	male	green	good
852	2011	Lillgrund	L1	52,40	male	green	good
853	2011	Lillgrund	L1	57,60	male	green	good
854	2011	Lillgrund	L1	53,70	male	green	good
855	2011	Lillgrund	L1	50,30	male	green	good
856	2011	Lillgrund	L1	52,50	male	green	good
857	2011	Lillgrund	L1	56,20	male	green	good
858	2011	Lillgrund	L1	56,70	male	green	good
859	2011	Lillgrund	L1	55,40	male	green	good
860	2011	Lillgrund	L1	49,90	male	green	good
861	2011	Lillgrund	L1	50,80	male	green	good
862	2011	Lillgrund	L1	57,70	male	green	good
863	2011	Lillgrund	L1	59,20	male	green	good
864	2011	Lillgrund	L1	46,90	male	green	good
865	2011	Lillgrund	L1	50,90	male	green	good
866	2011	Lillgrund	L1	67,40	male	green	good
867	2011	Lillgrund	L1	50,50	male	green	good
868	2011	Lillgrund	L1	59,70	male	red	good
869	2011	Lillgrund	L1	54,00	male	green	good
870	2011	Lillgrund	L1	50,50	male	green	good
871	2011	Lillgrund	L1	52,00	male	green	good
872	2011	Lillgrund	L1	50,50	male	green	good
873	2011	Lillgrund	L1	49,60	male	green	good
874	2011	Lillgrund	L1	40,00	male	green	good
875	2011	Lillgrund	L1	52,20	male	green	good
876	2011	Lillgrund	L1	44,20	male	green	good
877	2011	Lillgrund	L1	51,00	male	green	good
878	2011	Lillgrund	L1	70,41	male	green	good
879	2011	Lillgrund	L5	57,42	male	green	good
880	2011	Lillgrund	L5	59,66	male	green	good
881	2011	Lillgrund	L5	67,28	male	green	good

882	2011	Lillgrund	L5	54,69	male	red	good
883	2011	Lillgrund	L5	66,01	male	green	good
884	2011	Lillgrund	L5	53,71	male	red	good
885	2011	Lillgrund	L5	61,36	male	green	good
886	2011	Lillgrund	L5	50,40	male	green	good
887	2011	Lillgrund	L5	64,40	male	green	good
888	2011	Lillgrund	L5	51,20	male	green	good
889	2011	Lillgrund	L5	54,90	male	green	good
890	2011	Lillgrund	L5	61,50	male	green	good
891	2011	Lillgrund	L5	58,30	male	green	good
892	2011	Lillgrund	L5	62,30	male	green	good
893	2011	Lillgrund	L5	62,20	male	green	good
894	2011	Lillgrund	L5	57,90	male	green	good
895	2011	Lillgrund	L5	50,60	male	green	good
896	2011	Lillgrund	L5	64,90	male	green	good
897	2011	Lillgrund	L4	67,30	male	green	good
898	2011	Lillgrund	L4	56,90	male	green	good
899	2011	Lillgrund	L4	60,90	male	green	good
900	2011	Lillgrund	L4	53,60	male	green	good
901	2011	Lillgrund	L4	59,70	male	red	good
902	2011	Lillgrund	L4	57,00	male	green	good
903	2011	Lillgrund	L4	61,00	male	green	good
904	2011	Lillgrund	L4	65,90	male	green	good
905	2011	Lillgrund	L4	59,90	male	green	good
906	2011	Lillgrund	L4	46,20	male	green	good
907	2011	Lillgrund	L4	55,20	male	green	good
908	2011	Lillgrund	L4	52,44	male	green	good
909	2011	Lillgrund	L4	46,60	male	green	good
910	2011	Lillgrund	L2	68,40	male	green	good
911	2011	Lillgrund	L2	63,40	male	green	good
912	2011	Lillgrund	L2	67,70	male	green	good
913	2011	Lillgrund	L2	62,20	male	green	good
914	2011	Lillgrund	L2	68,60	male	green	good
915	2011	Lillgrund	L2	63,30	male	green	good
916	2011	Lillgrund	L2	63,80	male	green	good
917	2011	Lillgrund	L2	62,10	male	green	good
918	2011	Lillgrund	L2	60,90	male	green	good
919	2011	Lillgrund	L2	54,74	male	green	good
920	2011	Lillgrund	L2	62,60	male	green	good
921	2011	Lillgrund	L2	53,70	male	green	good
922	2011	Lillgrund	L2	58,50	male	green	good
923	2011	Lillgrund	L2	56,40	male	green	good
924	2011	Lillgrund	L2	58,20	male	green	good
925	2011	Lillgrund	L2	70,00	male	green	good
926	2011	Lillgrund	L2	59,50	male	green	good
927	2011	Lillgrund	L2	56,23	male	green	good
928	2011	Lillgrund	L2	66,30	male	green	good
929	2011	Lillgrund	L2	58,90	male	green	good
930	2011	Lillgrund	L2	66,60	male	green	good
931	2011	Lillgrund	L2	62,60	male	green	good
932	2011	Lillgrund	L2	60,50	male	green	good
933	2011	Lillgrund	L2	61,00	male	green	good
934	2011	Lillgrund	L2	56,40	male	green	good
935	2011	Lillgrund	L2	53,20	male	red	good
936	2011	Lillgrund	L2	55,35	male	red	good
937	2011	Lillgrund	L2	63,90	male	red	good
938	2011	Lillgrund	L2	59,80	male	green	good
939	2011	Lillgrund	L2	62,10	male	green	good
940	2011	Lillgrund	L2	68,80	male	green	good

941	2011	Lillgrund	L2	95,10	male	green	good
942	2011	Lillgrund	L2	60,40	male	green	good
943	2011	Lillgrund	L2	57,80	male	green	good
944	2011	Lillgrund	L2	56,50	male	green	good
945	2011	Lillgrund	L2	64,32	male	green	good
946	2011	Lillgrund	L2	61,20	male	green	good
947	2011	Lillgrund	L2	66,90	male	green	good
948	2011	Lillgrund	L2	54,10	male	green	good
949	2011	Lillgrund	L2	68,50	male	green	good
950	2011	Lillgrund	L2	52,00	male	green	good
951	2011	Lillgrund	L2	56,10	male	green	good
952	2011	Lillgrund	L2	63,30	male	green	good
953	2011	Lillgrund	L2	72,80	male	green	good
954	2011	Lillgrund	L2	52,90	male	green	good
955	2011	Lillgrund	L2	62,70	male	green	good
956	2011	Lillgrund	L2	56,90	male	green	good
957	2011	Lillgrund	L2	56,90	male	green	good
958	2011	Lillgrund	L2	41,50	male	green	good
959	2011	Lillgrund	L2	47,40	male	green	good
960	2011	Lillgrund	L1	69,70	male	green	good
961	2011	Lillgrund	L1	56,60	male	green	good
962	2011	Lillgrund	L1	62,20	male	green	good
963	2011	Lillgrund	L1	51,90	male	red	good
964	2011	Lillgrund	L1	52,10	male	red	good
965	2011	Lillgrund	L1	72,70	male	green	good
966	2011	Lillgrund	L1	55,90	male	green	good
967	2011	Lillgrund	L1	61,30	male	green	good
968	2011	Lillgrund	L1	58,50	male	green	good
969	2011	Lillgrund	L1	52,90	male	green	good
970	2011	Lillgrund	L1	56,10	male	green	good
971	2011	Lillgrund	L1	50,60	male	green	good
972	2011	Lillgrund	L1	55,50	male	green	good
973	2011	Lillgrund	L1	46,90	male	green	good
974	2011	Lillgrund	L1	59,30	male	red	good
975	2011	Lillgrund	L1	50,83	male	green	good
976	2011	Lillgrund	L1	43,00	male	green	good
977	2011	Lillgrund	L1	62,70	male	green	good
978	2011	Lillgrund	L1	59,78	male	green	good
979	2011	Lillgrund	L1	57,14	male	green	good
980	2011	Lillgrund	L1	57,80	male	green	good
981	2011	Lillgrund	L1	58,70	male	green	good
982	2011	Lillgrund	L1	52,28	male	green	good
983	2011	Lillgrund	L1	61,40	male	green	good
984	2011	Lillgrund	L1	58,30	male	green	good
985	2011	Lillgrund	L1	57,90	male	green	good
986	2011	Lillgrund	L1	71,30	male	green	good
987	2011	Lillgrund	L1	60,30	male	green	good
988	2011	Lillgrund	L5	58,70	male	green	good
989	2011	Lillgrund	L5	46,50	male	green	good
990	2011	Lillgrund	L5	50,80	male	green	good
991	2011	Lillgrund	L5	60,30	male	green	reduced
992	2011	Lillgrund	L5	47,60	male	green	good
993	2011	Lillgrund	L5	61,10	male	green	good
994	2011	Lillgrund	L5	64,90	male	green	good
995	2011	Lillgrund	L5	62,90	male	green	good
996	2011	Lillgrund	L5	55,40	male	green	good
997	2011	Lillgrund	L5	59,40	male	green	good
998	2011	Lillgrund	L5	54,30	male	green	good
999	2011	Lillgrund	L5	66,90	male	green	good

1000	2011	Lillgrund	L5	59,80	male	green	good
1001	2011	Lillgrund	L5	64,60	male	green	good
1002	2011	Lillgrund	L5	63,40	male	green	good
1003	2011	Lillgrund	L5	52,60	male	green	good
1004	2011	Lillgrund	L5	56,70	male	green	good
1005	2011	Lillgrund	L5	69,70	male	green	good
1006	2011	Lillgrund	L5	45,10	male	green	good
1007	2011	Lillgrund	L5	59,10	male	green	good
1008	2011	Lillgrund	L5	51,00	male	green	good
1009	2011	Lillgrund	L5	52,30	male	green	good
1010	2011	Lillgrund	L5	72,50	male	green	good
1011	2011	Lillgrund	L5	58,80	male	green	good
1012	2011	Lillgrund	L5	57,40	male	green	good
1013	2011	Lillgrund	L5	60,90	male	green	good
1014	2011	Lillgrund	L5	54,80	male	green	good
1015	2011	Lillgrund	L5	46,30	female	green	good
1016	2011	Lillgrund	L5	53,40	male	green	good
1017	2011	Lillgrund	L5	52,10	male	green	good
1018	2011	Lillgrund	L5	49,40	male	green	good
1019	2011	Lillgrund	L5	53,80	male	green	good
1020	2011	Lillgrund	L5	49,30	male	green	good
1021	2011	Lillgrund	L5	45,10	male	green	good
1022	2011	Lillgrund	L5	54,70	male	green	good
1023	2011	Lillgrund	L5	55,70	male	green	good
1024	2011	Lillgrund	L5	54,20	male	green	good
1025	2011	Lillgrund	L5	49,40	male	green	good
1026	2011	Lillgrund	L5	51,90	male	green	good
1027	2011	Lillgrund	L5	55,80	male	green	good
1028	2011	Lillgrund	L4	65,40	male	green	good
1029	2011	Lillgrund	L4	55,80	male	green	good
1030	2011	Lillgrund	L4	61,30	male	green	good
1031	2011	Lillgrund	L4	63,40	male	green	good
1032	2011	Lillgrund	L4	64,10	male	green	good
1033	2011	Lillgrund	L4	58,70	male	green	good
1034	2011	Lillgrund	L4	53,30	male	green	reduced
1035	2011	Lillgrund	L4	63,30	male	green	good
1036	2011	Lillgrund	L4	53,40	male	green	reduced
1037	2011	Lillgrund	L4	61,80	male	green	good
1038	2011	Lillgrund	L4	53,60	male	green	good
1039	2011	Lillgrund	L4	60,10	male	green	good
1040	2011	Lillgrund	L4	52,60	male	green	good
1041	2011	Lillgrund	L4	52,70	male	green	good
1042	2011	Lillgrund	L4	69,40	male	green	good
1043	2011	Lillgrund	L4	60,80	male	green	good
1044	2011	Lillgrund	L4	55,20	male	green	good
1045	2011	Lillgrund	L4	49,50	male	green	good
1046	2011	Lillgrund	L4	59,20	male	green	good
1047	2011	Lillgrund	L4	58,20	male	green	good
1048	2011	Lillgrund	L4	65,90	male	green	good
1049	2011	Lillgrund	L4	50,90	male	green	good
1050	2011	Lillgrund	L4	68,20	male	green	good
1051	2011	Lillgrund	L4	52,30	male	green	good
1052	2011	Lillgrund	L4	53,50	male	green	good
1053	2011	Lillgrund	L4	56,90	male	green	good
1054	2011	Lillgrund	L4	60,80	male	green	reduced
1055	2011	Lillgrund	L4	56,20	male	green	good
1056	2011	Lillgrund	L4	56,80	male	green	good
1057	2011	Lillgrund	L4	65,00	male	green	good
1058	2011	Lillgrund	L4	57,40	male	green	good

1059	2011	Lillgrund	L4	60,20	male	green	good
1060	2011	Lillgrund	L4	54,00	male	green	reduced
1061	2011	Lillgrund	L4	50,90	male	green	good
1062	2011	Lillgrund	L4	56,10	male	green	good
1063	2011	Lillgrund	L4	57,70	male	green	good
1064	2011	Lillgrund	L4	47,10	male	green	good
1065	2011	Lillgrund	L4	46,50	male	green	reduced
1066	2011	Lillgrund	L4	52,20	male	green	good
1067	2011	Bredgrund	B10	74,80	male	green	good
1068	2011	Bredgrund	B10	63,50	male	green	good
1069	2011	Bredgrund	B10	62,40	male	green	good
1070	2011	Bredgrund	B10	57,40	male	green	reduced
1071	2011	Bredgrund	B10	62,90	male	green	good
1072	2011	Bredgrund	B10	78,20	male	green	good
1073	2011	Bredgrund	B10	54,40	male	green	good
1074	2011	Bredgrund	B10	51,50	male	green	good
1075	2011	Bredgrund	B10	53,00	male	green	good
1076	2011	Bredgrund	B10	54,00	male	green	good
1077	2011	Bredgrund	B10	45,90	male	green	good
1078	2011	Bredgrund	B10	51,80	male	green	good
1079	2011	Bredgrund	B10	62,60	male	green	good
1080	2011	Bredgrund	B10	53,40	male	green	good
1081	2011	Bredgrund	B10	48,70	male	green	good
1082	2011	Bredgrund	B10	52,00	male	green	reduced
1083	2011	Bredgrund	B10	56,10	male	green	good
1084	2011	Bredgrund	B10	53,90	male	green	good
1085	2011	Bredgrund	B10	53,60	male	green	good
1086	2011	Bredgrund	B10	59,00	male	green	good
1087	2011	Bredgrund	B10	53,50	male	green	good
1088	2011	Bredgrund	B9	57,00	male	red	reduced
1089	2011	Bredgrund	B9	63,30	male	green	good
1090	2011	Bredgrund	B9	60,90	male	green	good
1091	2011	Bredgrund	B9	54,50	male	green	good
1092	2011	Bredgrund	B9	55,90	male	green	good
1093	2011	Bredgrund	B9	49,90	male	green	good
1094	2011	Bredgrund	B9	61,30	male	green	good
1095	2011	Bredgrund	B9	52,70	male	green	good
1096	2011	Bredgrund	B9	54,00	male	red	good
1097	2011	Bredgrund	B9	57,00	male	green	good
1098	2011	Bredgrund	B9	69,30	male	green	good
1099	2011	Bredgrund	B9	60,00	male	green	good
1100	2011	Bredgrund	B9	52,60	male	green	reduced
1101	2011	Bredgrund	B9	58,30	male	green	good
1102	2011	Bredgrund	B9	55,70	male	green	good
1103	2011	Bredgrund	B9	45,60	male	green	good
1104	2011	Bredgrund	B9	49,30	male	green	good
1105	2011	Bredgrund	B9	54,90	male	green	good
1106	2011	Bredgrund	B8	60,30	male	green	good
1107	2011	Bredgrund	B8	62,30	male	green	good
1108	2011	Bredgrund	B8	58,10	male	green	good
1109	2011	Bredgrund	B8	43,00	male	green	good
1110	2011	Bredgrund	B8	48,90	male	green	good
1111	2011	Bredgrund	B8	58,80	male	green	good
1112	2011	Bredgrund	B8	44,20	male	green	good
1113	2011	Bredgrund	B8	56,80	male	green	good
1114	2011	Bredgrund	B8	55,80	male	green	good
1115	2011	Bredgrund	B8	58,40	male	green	good
1116	2011	Bredgrund	B8	60,40	male	green	good
1117	2011	Bredgrund	B8	54,20	male	green	good

1118	2011	Bredgrund	B8	58,70	male	green	reduced
1119	2011	Bredgrund	B8	58,10	male	red	good
1120	2011	Bredgrund	B7	65,50	male	green	good
1121	2011	Bredgrund	B7	56,20	male	green	good
1122	2011	Bredgrund	B7	58,30	male	green	good
1123	2011	Bredgrund	B7	51,20	male	green	good
1124	2011	Bredgrund	B7	54,80	male	red	good
1125	2011	Bredgrund	B7	59,90	male	green	good
1126	2011	Bredgrund	B7	61,50	male	green	good
1127	2011	Bredgrund	B7	62,40	male	green	good
1128	2011	Bredgrund	B7	57,20	male	green	good
1129	2011	Bredgrund	B7	48,40	male	green	good
1130	2011	Bredgrund	B6	66,00	male	green	good
1131	2011	Bredgrund	B6	49,50	male	green	good
1132	2011	Bredgrund	B6	65,00	male	green	good
1133	2011	Bredgrund	B6	53,00	male	red	good
1134	2011	Bredgrund	B6	58,40	male	green	good
1135	2011	Bredgrund	B6	62,60	male	green	good
1136	2011	Bredgrund	B6	53,60	male	red	good
1137	2011	Bredgrund	B6	59,00	male	green	good
1138	2011	Bredgrund	B6	54,60	male	green	good
1139	2011	Bredgrund	B6	58,30	male	green	good
1140	2011	Bredgrund	B6	45,50	male	red	good
1141	2011	Bredgrund	B6	57,00	male	green	good
1142	2011	Bredgrund	B6	51,90	male	green	good
1143	2011	Bredgrund	B6	57,20	male	green	good
1144	2011	Bredgrund	B6	54,90	male	green	good
1145	2011	Bredgrund	B6	59,20	male	green	good
1146	2011	Bredgrund	B6	65,20	male	green	good
1147	2011	Bredgrund	B5	56,40	male	green	good
1148	2011	Bredgrund	B5	67,10	male	green	good
1149	2011	Bredgrund	B5	58,90	male	red	good
1150	2011	Bredgrund	B5	52,10	male	red	reduced
1151	2011	Bredgrund	B5	67,20	male	green	reduced
1152	2011	Bredgrund	B5	62,00	male	green	good
1153	2011	Bredgrund	B5	57,20	male	green	good
1154	2011	Bredgrund	B5	42,30	female	green	good
1155	2011	Bredgrund	B5	62,10	male	green	good
1156	2011	Bredgrund	B5	46,80	male	red	good
1157	2011	Bredgrund	B5	50,90	male	green	good
1158	2011	Bredgrund	B5	48,80	male	green	good
1159	2011	Bredgrund	B4	63,20	male	green	good
1160	2011	Bredgrund	B4	61,30	male	green	good
1161	2011	Bredgrund	B4	60,40	male	green	good
1162	2011	Bredgrund	B4	56,80	male	green	good
1163	2011	Bredgrund	B4	46,00	male	green	good
1164	2011	Bredgrund	B4	59,10	male	green	good
1165	2011	Bredgrund	B4	58,10	male	green	good
1166	2011	Bredgrund	B4	55,70	male	green	good
1167	2011	Bredgrund	B4	58,10	male	green	good
1168	2011	Bredgrund	B4	51,00	male	green	good
1169	2011	Bredgrund	B3	71,90	male	green	good
1170	2011	Bredgrund	B3	46,40	male	green	good
1171	2011	Bredgrund	B3	63,40	male	green	good
1172	2011	Bredgrund	B2	53,60	male	green	good
1173	2011	Bredgrund	B2	54,70	male	green	good
1174	2011	Bredgrund	B1	55,90	male	red	good
1175	2011	Bredgrund	B1	59,30	male	green	good
1176	2011	Bredgrund	B1	58,50	male	green	good

1177	2011	Bredgrund	B1	50,90	female	red	good
1178	2011	Bredgrund	B10	57,10	male	red	good
1179	2011	Bredgrund	B10	54,90	male	green	reduced
1180	2011	Bredgrund	B10	67,90	male	green	good
1181	2011	Bredgrund	B10	58,10	female	red	good
1182	2011	Bredgrund	B10	51,90	male	red	good
1183	2011	Bredgrund	B9	50,40	male	green	good
1184	2011	Bredgrund	B9	49,00	male	green	good
1185	2011	Bredgrund	B9	53,50	male	green	good
1186	2011	Bredgrund	B9	66,70	male	green	good
1187	2011	Bredgrund	B9	53,90	male	green	good
1188	2011	Bredgrund	B9	48,10	male	green	good
1189	2011	Bredgrund	B9	52,80	male	green	good
1190	2011	Bredgrund	B9	60,00	male	green	good
1191	2011	Bredgrund	B9	62,70	male	green	good
1192	2011	Bredgrund	B8	51,60	male	green	good
1193	2011	Bredgrund	B8	60,10	male	green	reduced
1194	2011	Bredgrund	B8	49,60	male	green	good
1195	2011	Bredgrund	B8	52,40	male	green	good
1196	2011	Bredgrund	B8	61,00	male	green	good
1197	2011	Bredgrund	B8	46,40	male	green	good
1198	2011	Bredgrund	B8	62,90	male	green	good
1199	2011	Bredgrund	B8	66,30	male	green	good
1200	2011	Bredgrund	B8	53,40	male	green	reduced
1201	2011	Bredgrund	B7	53,60	male	green	reduced
1202	2011	Bredgrund	B7	50,60	male	green	good
1203	2011	Bredgrund	B7	50,30	male	green	good
1204	2011	Bredgrund	B6	59,80	male	green	good
1205	2011	Bredgrund	B6	54,30	male	green	good
1206	2011	Bredgrund	B6	58,50	male	green	good
1207	2011	Bredgrund	B6	50,70	male	green	good
1208	2011	Bredgrund	B6	46,30	female	green	reduced
1209	2011	Bredgrund	B6	56,70	male	red	good
1210	2011	Bredgrund	B6	54,40	male	green	good
1211	2011	Bredgrund	B6	53,70	male	green	good
1212	2011	Bredgrund	B6	50,90	male	green	good
1213	2011	Bredgrund	B6	57,70	male	green	good
1214	2011	Bredgrund	B6	45,30	male	green	good
1215	2011	Bredgrund	B5	52,70	male	green	good
1216	2011	Bredgrund	B5	61,50	male	green	good
1217	2011	Bredgrund	B5	58,80	male	green	good
1218	2011	Bredgrund	B3	58,70	male	green	good
1219	2011	Bredgrund	B1	65,90	male	green	reduced
1220	2011	Bredgrund	B1	54,20	male	green	good
1221	2011	Bredgrund	B1	56,30	male	green	good
1222	2011	Bredgrund	B4	67,80	male	green	good
1223	2011	Bredgrund	B4	50,70	male	green	good
1224	2011	Bredgrund	B4	52,80	male	green	good
1225	2011	Bredgrund	B4	57,20	male	red	reduced
1226	2011	Bredgrund	B4	47,60	male	green	good
1227	2011	Bredgrund	B4	66,50	male	green	good
1228	2011	Bredgrund	B4	64,90	male	green	good
1229	2011	Bredgrund	B4	63,40	male	green	good
1230	2011	Bredgrund	B4	60,70	male	green	good
1231	2011	Sjollen	S7	32,00	male	green	good
1232	2011	Sjollen	S7	36,70	female	red	good
1233	2011	Sjollen	S7	42,80	male	green	good
1234	2011	Sjollen	S7	44,10	male	green	reduced
1235	2011	Sjollen	S7	50,70	male	green	good

1236	2011	Sjollen	S7	63,30	male	green	good
1237	2011	Sjollen	S7	49,50	male	green	good
1238	2011	Sjollen	S7	50,00	male	green	good
1239	2011	Sjollen	S4	54,60	male	red	good
1240	2011	Sjollen	S4	48,80	male	green	good
1241	2011	Sjollen	S4	51,60	male	green	good
1242	2011	Sjollen	S4	60,30	male	green	good
1243	2011	Sjollen	S4	54,20	male	green	good
1244	2011	Sjollen	S4	50,40	male	green	good
1245	2011	Sjollen	S4	63,80	male	green	good
1246	2011	Sjollen	S4	50,00	male	green	good
1247	2011	Sjollen	S4	59,20	male	red	good
1248	2011	Sjollen	S4	65,00	male	green	good
1249	2011	Sjollen	S4	46,47	male	green	good
1250	2011	Sjollen	S4	54,30	male	green	good
1251	2011	Sjollen	S4	57,90	male	green	good
1252	2011	Sjollen	S4	58,50	male	green	good
1253	2011	Sjollen	S4	54,30	male	green	good
1254	2011	Sjollen	S4	51,60	male	green	good
1255	2011	Sjollen	S4	44,70	male	green	good
1256	2011	Sjollen	S1	52,40	male	green	good
1257	2011	Sjollen	S1	60,50	male	green	reduced
1258	2011	Sjollen	S1	59,00	male	green	good
1259	2011	Sjollen	S1	58,10	male	green	good
1260	2011	Sjollen	S1	63,10	male	green	good
1261	2011	Sjollen	S1	65,00	male	green	good
1262	2011	Sjollen	S1	53,90	male	green	good
1263	2011	Sjollen	S1	51,56	male	green	good
1264	2011	Sjollen	S1	61,40	male	green	good
1265	2011	Sjollen	S1	58,10	male	green	good
1266	2011	Sjollen	S1	65,20	male	green	good
1267	2011	Sjollen	S1	62,50	male	green	good
1268	2011	Sjollen	S1	49,80	male	green	good
1269	2011	Sjollen	S1	51,10	male	green	good
1270	2011	Sjollen	S1	47,80	male	green	good
1271	2011	Sjollen	S1	53,40	male	green	reduced
1272	2011	Sjollen	S1	62,00	male	green	good
1273	2011	Sjollen	S1	67,50	male	green	good
1274	2011	Sjollen	S1	54,90	male	green	good
1275	2011	Sjollen	S1	70,10	male	green	good
1276	2011	Sjollen	S1	53,00	male	green	good
1277	2011	Sjollen	S1	58,70	male	green	good
1278	2011	Sjollen	S1	42,90	male	green	good
1279	2011	Sjollen	S1	45,00	male	green	good
1280	2011	Sjollen	S2	56,40	male	green	good
1281	2011	Sjollen	S2	56,30	male	green	good
1282	2011	Sjollen	S2	69,00	male	green	reduced
1283	2011	Sjollen	S2	61,10	male	green	good
1284	2011	Sjollen	S2	57,20	male	green	good
1285	2011	Sjollen	S2	61,60	male	green	good
1286	2011	Sjollen	S2	64,40	male	green	good
1287	2011	Sjollen	S2	56,60	male	green	good
1288	2011	Sjollen	S2	61,50	male	green	good
1289	2011	Sjollen	S2	63,50	male	green	reduced
1290	2011	Sjollen	S2	61,60	male	green	good
1291	2011	Sjollen	S2	62,60	male	green	good
1292	2011	Sjollen	S2	58,30	male	green	good
1293	2011	Sjollen	S2	62,90	male	green	good
1294	2011	Sjollen	S2	51,20	male	green	good

1295	2011	Sjollen	S2	38,30	female	red	good
1296	2011	Sjollen	S2	56,30	male	green	good
1297	2011	Sjollen	S2	50,10	male	green	good
1298	2011	Sjollen	S2	55,00	male	red	good
1299	2011	Sjollen	S2	60,10	male	green	reduced
1300	2011	Sjollen	S2	61,00	male	green	good
1301	2011	Sjollen	S2	52,80	male	green	good
1302	2011	Sjollen	S3	59,50	male	green	good
1303	2011	Sjollen	S3	60,70	male	green	good
1304	2011	Sjollen	S3	65,70	male	green	good
1305	2011	Sjollen	S3	60,30	male	green	good
1306	2011	Sjollen	S3	63,80	male	green	good
1307	2011	Sjollen	S3	64,40	male	green	good
1308	2011	Sjollen	S3	62,70	male	green	reduced
1309	2011	Sjollen	S3	62,90	male	green	good
1310	2011	Sjollen	S3	52,50	male	green	good
1311	2011	Sjollen	S3	62,90	male	green	good
1312	2011	Sjollen	S3	51,40	male	green	good
1313	2011	Sjollen	S3	49,70	male	green	good
1314	2011	Sjollen	S3	50,50	male	green	good
1315	2011	Sjollen	S3	63,60	male	green	good
1316	2011	Sjollen	S3	60,60	male	green	reduced
1317	2011	Sjollen	S3	52,50	male	green	good
1318	2011	Sjollen	S3	57,20	male	green	good
1319	2011	Sjollen	S3	64,40	male	green	good
1320	2011	Sjollen	S3	55,20	male	green	good
1321	2011	Sjollen	S3	49,10	male	green	good
1322	2011	Sjollen	S3	55,80	male	green	good
1323	2011	Sjollen	S3	56,00	male	green	good
1324	2011	Sjollen	S3	57,60	male	red	good
1325	2011	Sjollen	S5	63,70	male	green	good
1326	2011	Sjollen	S5	57,40	male	green	reduced
1327	2011	Sjollen	S5	45,50	male	green	good
1328	2011	Sjollen	S5	55,80	male	green	good
1329	2011	Sjollen	S5	62,60	male	green	good
1330	2011	Sjollen	S5	75,30	male	green	good
1331	2011	Sjollen	S5	65,90	male	green	good
1332	2011	Sjollen	S5	57,20	male	green	good
1333	2011	Sjollen	S5	54,00	male	red	good
1334	2011	Sjollen	S5	71,20	male	green	good
1335	2011	Sjollen	S5	67,70	male	green	good
1336	2011	Sjollen	S5	57,40	male	green	good
1337	2011	Sjollen	S5	66,50	male	green	good
1338	2011	Sjollen	S5	48,60	male	green	good
1339	2011	Sjollen	S5	54,90	male	green	good
1340	2011	Sjollen	S5	48,90	male	green	good
1341	2011	Sjollen	S5	52,30	male	green	good
1342	2011	Sjollen	S5	55,10	male	green	good
1343	2011	Sjollen	S5	46,90	male	green	good
1344	2011	Sjollen	S5	60,60	male	green	good
1345	2011	Sjollen	S5	56,20	male	green	good
1346	2011	Sjollen	S5	55,90	male	green	reduced
1347	2011	Sjollen	S5	53,90	male	green	reduced
1348	2011	Sjollen	S5	55,00	male	green	good
1349	2011	Sjollen	S5	69,90	male	green	good
1350	2011	Sjollen	S5	48,70	male	red	good
1351	2011	Sjollen	S5	60,60	male	green	good
1352	2011	Sjollen	S5	58,70	male	green	reduced
1353	2011	Sjollen	S5	47,70	male	green	good

1354	2011	Sjollen	S5	58,20	male	red	good
1355	2011	Sjollen	S5	54,60	male	green	good
1356	2011	Sjollen	S5	45,70	female	green	good
1357	2011	Sjollen	S6	61,60	male	green	good
1358	2011	Sjollen	S6	62,20	male	green	good
1359	2011	Sjollen	S6	63,60	male	green	good
1360	2011	Sjollen	S6	63,30	male	green	good
1361	2011	Sjollen	S6	61,80	male	green	good
1362	2011	Sjollen	S6	62,50	male	green	good
1363	2011	Sjollen	S6	61,90	male	green	reduced
1364	2011	Sjollen	S6	62,40	male	green	good
1365	2011	Sjollen	S6	56,40	male	green	good
1366	2011	Sjollen	S6	63,80	male	green	good
1367	2011	Sjollen	S6	62,20	male	green	good
1368	2011	Sjollen	S6	65,30	male	green	reduced
1369	2011	Sjollen	S6	67,00	male	green	good
1370	2011	Sjollen	S6	60,40	male	green	good
1371	2011	Sjollen	S6	68,10	male	green	good
1372	2011	Sjollen	S6	63,70	male	green	good
1373	2011	Sjollen	S6	64,60	male	green	good
1374	2011	Sjollen	S6	65,00	male	red	good
1375	2011	Sjollen	S6	45,60	male	green	good
1376	2011	Sjollen	S6	65,30	male	green	reduced
1377	2011	Sjollen	S6	50,40	male	green	good
1378	2011	Sjollen	S6	47,00	male	green	good
1379	2011	Sjollen	S6	56,80	male	green	good
1380	2011	Sjollen	S6	61,40	male	green	good
1381	2011	Sjollen	S6	54,60	male	green	good
1382	2011	Sjollen	S6	63,80	male	green	reduced
1383	2011	Sjollen	S6	63,20	male	green	good
1384	2011	Sjollen	S6	65,40	male	green	good
1385	2011	Sjollen	S6	56,10	male	green	good
1386	2011	Sjollen	S6	48,90	male	green	good
1387	2011	Sjollen	S6	55,70	male	red	good
1388	2011	Sjollen	S6	67,30	male	green	good
1389	2011	Sjollen	S6	54,40	male	green	good
1390	2011	Sjollen	S6	48,40	female	green	good
1391	2011	Sjollen	S6	63,80	male	red	good
1392	2011	Sjollen	S10	63,90	male	green	good
1393	2011	Sjollen	S10	60,50	male	green	good
1394	2011	Sjollen	S10	54,30	male	green	good
1395	2011	Sjollen	S10	62,40	male	green	good
1396	2011	Sjollen	S10	69,90	male	green	good
1397	2011	Sjollen	S10	62,00	male	green	reduced
1398	2011	Sjollen	S10	59,20	male	green	good
1399	2011	Sjollen	S10	59,60	male	red	good
1400	2011	Sjollen	S10	54,00	male	green	good
1401	2011	Sjollen	S10	57,00	male	green	good
1402	2011	Sjollen	S10	56,50	male	green	reduced
1403	2011	Sjollen	S10	67,80	male	green	reduced
1404	2011	Sjollen	S10	59,30	male	green	good
1405	2011	Sjollen	S10	64,10	male	green	good
1406	2011	Sjollen	S10	46,80	male	green	good
1407	2011	Sjollen	S10	59,70	male	green	good
1408	2011	Sjollen	S10	58,30	male	green	good
1409	2011	Sjollen	S10	56,00	male	green	good
1410	2011	Sjollen	S10	54,50	male	green	good
1411	2011	Sjollen	S10	68,40	male	green	good
1412	2011	Sjollen	S10	56,30	male	red	reduced

1413	2011	Sjollen	S10	63,60	male	green	good
1414	2011	Sjollen	S10	50,80	male	green	good
1415	2011	Sjollen	S10	55,30	male	green	good
1416	2011	Sjollen	S10	50,90	male	green	good
1417	2011	Sjollen	S10	56,30	male	green	good
1418	2011	Sjollen	S10	43,70	male	green	good
1419	2011	Sjollen	S10	54,40	male	green	good
1420	2011	Sjollen	S10	48,80	male	green	reduced
1421	2011	Sjollen	S10	47,70	male	green	good
1422	2011	Sjollen	S10	47,10	male	green	good
1423	2011	Sjollen	S10	37,80	female	green	good
1424	2011	Sjollen	S10	42,80	male	green	reduced
1425	2011	Sjollen	S9	53,20	male	green	good
1426	2011	Sjollen	S9	56,80	male	green	good
1427	2011	Sjollen	S9	49,70	male	green	good
1428	2011	Sjollen	S9	57,60	male	green	reduced
1429	2011	Sjollen	S9	60,70	male	green	good
1430	2011	Sjollen	S9	60,70	male	green	good
1431	2011	Sjollen	S9	55,20	male	green	good
1432	2011	Sjollen	S9	46,00	male	green	good
1433	2011	Sjollen	S9	49,20	male	green	good
1434	2011	Sjollen	S9	50,00	male	green	reduced
1435	2011	Sjollen	S9	40,70	male	green	good
1436	2011	Sjollen	S8	65,60	male	green	reduced
1437	2011	Sjollen	S8	64,10	male	green	good
1438	2011	Sjollen	S8	61,90	male	green	good
1439	2011	Sjollen	S8	70,70	male	green	good
1440	2011	Sjollen	S8	63,00	male	red	good
1441	2011	Sjollen	S8	63,90	male	green	good
1442	2011	Sjollen	S8	63,90	male	green	good
1443	2011	Sjollen	S8	62,90	male	green	good
1444	2011	Sjollen	S8	63,40	male	green	good
1445	2011	Sjollen	S8	51,20	male	red	good
1446	2011	Sjollen	S8	53,80	male	green	good
1447	2011	Sjollen	S8	52,90	male	green	good
1448	2011	Sjollen	S8	50,70	male	green	good
1449	2011	Sjollen	S8	45,30	male	green	good
1450	2011	Sjollen	S8	49,50	male	green	reduced
1451	2011	Sjollen	S8	49,40	male	green	good
1452	2011	Sjollen	S8	60,70	male	green	good
1453	2011	Sjollen	S8	53,20	male	red	good
1454	2011	Sjollen	S8	49,10	male	green	good
1455	2011	Sjollen	S8	41,80	female	red	good
1456	2011	Sjollen	S8	52,30	male	green	good
1457	2011	Sjollen	S8	47,50	male	green	reduced
1458	2011	Sjollen	S8	53,90	male	green	good
1459	2011	Sjollen	S8	45,20	male	green	good
1460	2011	Sjollen	S8	46,70	male	green	good
1461	2011	Lillgrund	L7	56,10	male	green	good
1462	2011	Lillgrund	L7	67,20	male	green	good
1463	2011	Lillgrund	L7	68,40	male	green	good
1464	2011	Lillgrund	L7	65,20	male	green	good
1465	2011	Lillgrund	L7	58,90	male	green	good
1466	2011	Lillgrund	L7	58,20	male	green	good
1467	2011	Lillgrund	L7	61,20	male	green	good
1468	2011	Lillgrund	L7	60,80	male	green	good
1469	2011	Lillgrund	L7	63,90	male	green	good
1470	2011	Lillgrund	L7	60,60	male	green	good
1471	2011	Lillgrund	L7	51,30	male	green	good

1472	2011	Lillgrund	L7	53,40	male	green	reduced
1473	2011	Lillgrund	L7	62,50	male	green	good
1474	2011	Lillgrund	L7	63,30	male	green	good
1475	2011	Lillgrund	L8	62,20	male	green	good
1476	2011	Lillgrund	L8	72,20	male	green	reduced
1477	2011	Lillgrund	L8	58,90	male	green	good
1478	2011	Lillgrund	L8	52,00	male	green	reduced
1479	2011	Lillgrund	L8	64,50	male	green	good
1480	2011	Lillgrund	L8	57,90	male	green	good
1481	2011	Lillgrund	L8	57,30	male	green	good
1482	2011	Lillgrund	L9	69,70	male	green	good
1483	2011	Lillgrund	L9	70,80	male	green	good
1484	2011	Lillgrund	L9	68,40	male	green	good
1485	2011	Lillgrund	L9	72,70	male	green	good
1486	2011	Lillgrund	L9	57,00	male	red	good
1487	2011	Lillgrund	L9	64,30	male	green	reduced
1488	2011	Lillgrund	L9	60,70	male	green	reduced
1489	2011	Lillgrund	L9	67,00	male	green	good
1490	2011	Lillgrund	L9	67,20	male	green	good
1491	2011	Lillgrund	L9	61,70	male	green	reduced
1492	2011	Lillgrund	L9	63,50	male	green	good
1493	2011	Lillgrund	L9	59,30	male	green	good
1494	2011	Lillgrund	L9	64,90	male	green	good
1495	2011	Lillgrund	L9	56,40	male	red	good
1496	2011	Lillgrund	L9	54,30	male	red	good
1497	2011	Lillgrund	L9	60,20	male	green	good
1498	2011	Lillgrund	L9	67,60	male	green	good
1499	2011	Lillgrund	L9	59,80	male	green	good
1500	2011	Lillgrund	L9	56,10	male	green	reduced
1501	2011	Lillgrund	L9	65,50	male	green	good
1502	2011	Lillgrund	L9	60,40	male	green	good
1503	2011	Lillgrund	L9	55,90	male	green	reduced
1504	2011	Lillgrund	L9	64,80	male	green	good
1505	2011	Lillgrund	L9	59,80	male	green	reduced
1506	2011	Lillgrund	L9	64,90	male	green	good
1507	2011	Lillgrund	L9	53,90	male	green	good
1508	2011	Lillgrund	L9	64,90	male	green	good
1509	2011	Lillgrund	L9	60,80	male	green	reduced
1510	2011	Lillgrund	L9	54,00	male	green	good
1511	2011	Lillgrund	L10	61,40	male	green	good
1512	2011	Lillgrund	L10	55,40	male	green	good
1513	2011	Lillgrund	L10	68,00	male	green	good
1514	2011	Lillgrund	L10	61,20	male	green	good
1515	2011	Lillgrund	L10	59,20	male	green	good
1516	2011	Lillgrund	L10	51,00	male	green	good
1517	2011	Lillgrund	L10	53,60	male	green	good
1518	2011	Lillgrund	L10	60,50	male	red	reduced
1519	2011	Lillgrund	L10	52,90	male	green	good
1520	2011	Lillgrund	L10	46,20	male	green	good
1521	2011	Lillgrund	L10	49,20	male	green	good
1522	2011	Lillgrund	L10	54,70	male	green	good
1523	2011	Lillgrund	L10	61,50	male	green	reduced
1524	2011	Lillgrund	L10	51,60	male	green	good
1525	2011	Lillgrund	L6	64,00	male	green	good
1526	2011	Lillgrund	L6	51,50	male	green	good
1527	2011	Lillgrund	L6	65,00	male	green	good
1528	2011	Lillgrund	L6	59,50	male	green	reduced
1529	2011	Lillgrund	L6	59,20	male	green	good
1530	2011	Lillgrund	L6	53,40	male	red	good

1531	2011	Lillgrund	L6	61,90	male	green	good
1532	2011	Lillgrund	L6	60,50	male	green	good
1533	2011	Lillgrund	L6	45,40	male	green	good
1534	2011	Lillgrund	L6	63,50	male	green	good
1535	2011	Lillgrund	L6	54,10	male	green	good
1536	2011	Lillgrund	L6	56,60	male	green	good
1537	2011	Lillgrund	L6	58,00	male	green	good
1538	2011	Lillgrund	L6	57,20	male	green	good
1539	2011	Lillgrund	L6	64,60	male	green	good
1540	2011	Lillgrund	L6	57,70	male	green	reduced
1541	2011	Lillgrund	L6	64,60	male	green	good
1542	2011	Lillgrund	L6	49,30	male	green	good
1543	2011	Lillgrund	L6	51,70	male	green	good
1544	2011	Lillgrund	L6	57,10	male	green	good
1545	2011	Lillgrund	L6	49,90	male	green	good
1546	2011	Lillgrund	L6	53,60	male	green	good
1547	2011	Lillgrund	L6	53,40	male	green	good
1548	2011	Lillgrund	L6	48,40	male	green	good
1549	2011	Lillgrund	L6	59,30	male	red	good
1550	2011	Lillgrund	L6	54,90	male	green	good
1551	2011	Lillgrund	L6	58,90	male	green	good
1552	2011	Lillgrund	L6	58,00	male	red	good
1553	2011	Lillgrund	L6	56,10	male	green	good
1554	2011	Lillgrund	L6	42,50	female	red	good
1555	2011	Lillgrund	L6	40,90	female	green	good
1556	2011	Lillgrund	L5	59,50	male	green	good
1557	2011	Lillgrund	L5	58,60	male	green	good
1558	2011	Lillgrund	L5	60,80	male	red	good
1559	2011	Lillgrund	L5	62,00	male	green	good
1560	2011	Lillgrund	L5	56,80	male	red	reduced
1561	2011	Lillgrund	L5	62,40	male	green	good
1562	2011	Lillgrund	L5	58,30	male	green	good
1563	2011	Lillgrund	L5	60,10	male	green	good
1564	2011	Lillgrund	L5	60,10	male	green	good
1565	2011	Lillgrund	L5	64,80	male	green	good
1566	2011	Lillgrund	L5	61,90	male	green	good
1567	2011	Lillgrund	L5	52,10	male	green	good
1568	2011	Lillgrund	L5	61,90	male	green	good
1569	2011	Lillgrund	L5	62,90	male	green	good
1570	2011	Lillgrund	L5	56,20	male	red	reduced
1571	2011	Lillgrund	L5	62,30	male	green	reduced
1572	2011	Lillgrund	L5	49,90	male	green	good
1573	2011	Lillgrund	L5	62,20	male	green	good
1574	2011	Lillgrund	L5	68,90	male	green	good
1575	2011	Lillgrund	L5	63,80	male	green	good
1576	2011	Lillgrund	L5	66,30	male	green	good
1577	2011	Lillgrund	L5	63,30	male	green	good
1578	2011	Lillgrund	L5	51,90	male	green	reduced
1579	2011	Lillgrund	L5	69,40	male	green	reduced
1580	2011	Lillgrund	L5	55,70	male	green	good
1581	2011	Lillgrund	L5	54,80	male	green	good
1582	2011	Lillgrund	L5	45,50	female	green	good
1583	2011	Lillgrund	L5	69,30	male	green	good
1584	2011	Lillgrund	L5	52,40	male	green	good
1585	2011	Lillgrund	L5	64,70	male	green	good
1586	2011	Lillgrund	L5	68,20	male	green	good
1587	2011	Lillgrund	L5	56,70	male	green	good
1588	2011	Lillgrund	L5	62,10	male	green	good
1589	2011	Lillgrund	L5	61,20	male	green	good

1590	2011	Lillgrund	L5	62,20	male	green	good
1591	2011	Lillgrund	L5	61,50	male	green	reduced
1592	2011	Lillgrund	L5	63,00	male	green	good
1593	2011	Lillgrund	L5	53,50	male	green	good
1594	2011	Lillgrund	L5	55,90	male	green	good
1595	2011	Lillgrund	L5	53,20	male	green	good
1596	2011	Lillgrund	L5	67,60	male	green	reduced
1597	2011	Lillgrund	L5	56,20	male	green	good
1598	2011	Lillgrund	L5	55,30	male	green	good
1599	2011	Lillgrund	L5	56,60	male	green	good
1600	2011	Lillgrund	L5	52,70	male	green	good
1601	2011	Lillgrund	L3	66,60	male	green	reduced
1602	2011	Lillgrund	L3	60,40	male	green	good
1603	2011	Lillgrund	L3	66,00	male	green	reduced
1604	2011	Lillgrund	L3	66,00	male	green	good
1605	2011	Lillgrund	L3	62,30	male	green	good
1606	2011	Lillgrund	L3	64,00	male	green	good
1607	2011	Lillgrund	L3	67,80	male	green	good
1608	2011	Lillgrund	L3	58,40	male	green	good
1609	2011	Lillgrund	L3	57,80	male	green	reduced
1610	2011	Lillgrund	L3	70,00	male	green	good
1611	2011	Lillgrund	L3	66,00	male	green	good
1612	2011	Lillgrund	L3	63,20	male	green	good
1613	2011	Lillgrund	L3	55,40	male	green	good
1614	2011	Lillgrund	L3	65,40	male	green	good
1615	2011	Lillgrund	L3	66,90	male	green	reduced
1616	2011	Lillgrund	L3	70,30	male	green	good
1617	2011	Lillgrund	L3	60,40	male	green	good
1618	2011	Lillgrund	L3	64,70	male	green	reduced
1619	2011	Lillgrund	L3	67,00	male	green	good
1620	2011	Lillgrund	L3	64,30	male	green	good
1621	2011	Lillgrund	L3	63,70	male	green	good
1622	2011	Lillgrund	L3	68,50	male	green	good
1623	2011	Lillgrund	L3	70,90	male	green	good
1624	2011	Lillgrund	L3	62,00	male	green	good
1625	2011	Lillgrund	L3	54,80	male	green	good
1626	2011	Lillgrund	L3	65,80	male	green	good
1627	2011	Lillgrund	L3	62,70	male	green	good
1628	2011	Lillgrund	L3	60,20	male	green	good
1629	2011	Lillgrund	L3	56,60	male	green	good
1630	2011	Lillgrund	L3	62,30	male	green	good
1631	2011	Lillgrund	L3	57,50	male	green	good
1632	2011	Lillgrund	L3	66,80	male	green	reduced
1633	2011	Lillgrund	L3	58,67	male	green	good
1634	2011	Lillgrund	L3	62,50	male	green	good
1635	2011	Lillgrund	L3	66,10	male	green	good
1636	2011	Lillgrund	L3	57,70	male	green	good
1637	2011	Lillgrund	L3	55,90	male	green	good
1638	2011	Lillgrund	L3	54,90	male	green	good
1639	2011	Lillgrund	L3	61,60	male	green	good
1640	2011	Lillgrund	L3	59,70	male	red	good
1641	2011	Lillgrund	L3	52,50	male	green	good
1642	2011	Lillgrund	L3	65,80	male	green	good
1643	2011	Lillgrund	L3	64,50	male	green	good
1644	2011	Lillgrund	L3	53,90	male	green	reduced
1645	2011	Lillgrund	L3	61,50	male	green	good
1646	2011	Lillgrund	L3	64,60	male	green	good
1647	2011	Lillgrund	L3	59,80	male	green	good
1648	2011	Lillgrund	L3	69,00	male	green	good

1649	2011	Lillgrund	L3	52,40	male	green	good
1650	2011	Lillgrund	L3	56,70	male	green	good
1651	2011	Lillgrund	L3	63,30	male	green	good
1652	2011	Lillgrund	L3	61,30	male	green	good
1653	2011	Lillgrund	L3	56,00	male	green	good
1654	2011	Lillgrund	L2	60,60	male	green	reduced
1655	2011	Lillgrund	L2	73,30	male	green	good
1656	2011	Lillgrund	L2	58,70	male	green	good
1657	2011	Lillgrund	L2	47,10	male	green	good
1658	2011	Lillgrund	L2	65,10	male	green	good
1659	2011	Lillgrund	L2	55,10	male	green	good
1660	2011	Lillgrund	L2	47,40	male	green	good
1661	2011	Lillgrund	L2	64,90	male	green	reduced
1662	2011	Lillgrund	L2	49,40	male	green	good
1663	2011	Lillgrund	L2	54,40	male	green	good
1664	2011	Lillgrund	L2	47,10	male	green	good
1665	2011	Lillgrund	L2	50,80	male	green	good
1666	2011	Lillgrund	L2	50,00	male	green	good
1667	2011	Lillgrund	L2	49,30	male	green	good
1668	2011	Lillgrund	L2	46,30	male	green	good
1669	2011	Lillgrund	L2	52,40	male	green	good
1670	2011	Lillgrund	L4	67,50	male	green	good
1671	2011	Lillgrund	L4	68,90	male	green	good
1672	2011	Lillgrund	L4	64,00	male	green	good
1673	2011	Lillgrund	L4	66,90	male	green	good
1674	2011	Lillgrund	L4	60,80	male	green	good
1675	2011	Lillgrund	L4	62,20	male	red	good
1676	2011	Lillgrund	L4	53,50	male	green	good
1677	2011	Lillgrund	L4	59,70	male	green	good
1678	2011	Lillgrund	L4	61,50	male	green	good
1679	2011	Lillgrund	L4	64,70	male	green	good
1680	2011	Lillgrund	L4	54,60	male	green	good
1681	2011	Lillgrund	L4	53,50	male	green	reduced
1682	2011	Lillgrund	L4	52,90	male	green	good
1683	2011	Lillgrund	L4	65,90	male	green	good
1684	2011	Lillgrund	L4	71,30	male	green	good
1685	2011	Lillgrund	L4	57,00	male	green	good
1686	2011	Lillgrund	L4	59,90	male	green	good
1687	2011	Lillgrund	L4	53,90	male	green	good
1688	2011	Lillgrund	L4	58,60	male	green	good
1689	2011	Lillgrund	L4	63,90	male	green	good
1690	2011	Lillgrund	L4	55,70	male	green	good
1691	2011	Lillgrund	L4	61,30	male	green	good
1692	2011	Lillgrund	L4	62,40	male	green	good
1693	2011	Lillgrund	L4	61,70	male	green	good
1694	2011	Lillgrund	L4	59,50	male	green	good
1695	2011	Lillgrund	L4	58,80	male	green	reduced
1696	2011	Lillgrund	L4	59,50	male	green	good
1697	2011	Lillgrund	L4	64,20	male	green	good
1698	2011	Lillgrund	L4	55,70	male	green	good
1699	2011	Lillgrund	L4	59,90	male	green	good
1700	2011	Lillgrund	L4	60,20	male	green	good
1701	2011	Lillgrund	L4	58,20	male	green	good
1702	2011	Lillgrund	L4	48,50	male	green	good
1703	2011	Lillgrund	L4	53,20	male	green	good
1704	2011	Lillgrund	L4	57,30	male	green	good
1705	2011	Lillgrund	L4	51,70	male	green	good
1706	2011	Lillgrund	L4	56,50	male	green	reduced
1707	2011	Lillgrund	L4	55,90	female	green	good

1708	2011	Lillgrund	L4	50,60	male	green	good
1709	2011	Lillgrund	L4	41,60	male	green	good
1710	2011	Lillgrund	L1	53,80	male	green	good
1711	2011	Lillgrund	L1	67,30	male	green	good
1712	2011	Lillgrund	L1	70,50	male	green	good
1713	2011	Lillgrund	L1	59,20	male	green	good
1714	2011	Lillgrund	L1	58,80	male	green	good
1715	2011	Lillgrund	L1	67,50	male	green	good
1716	2011	Lillgrund	L1	64,60	male	green	good
1717	2011	Lillgrund	L1	68,10	male	green	good
1718	2011	Lillgrund	L1	62,80	male	green	good
1719	2011	Lillgrund	L1	68,10	male	green	good
1720	2011	Lillgrund	L1	63,90	male	green	good
1721	2011	Lillgrund	L1	61,00	male	green	good
1722	2011	Lillgrund	L1	65,20	male	green	good
1723	2011	Lillgrund	L1	60,40	male	green	reduced
1724	2011	Lillgrund	L1	63,70	male	green	good
1725	2011	Lillgrund	L1	61,80	male	green	good
1726	2011	Lillgrund	L1	62,40	male	green	good
1727	2011	Lillgrund	L1	53,60	male	green	good
1728	2011	Lillgrund	L1	70,80	male	green	good
1729	2011	Lillgrund	L1	58,90	male	green	good
1730	2011	Lillgrund	L1	59,60	male	green	good
1731	2011	Lillgrund	L1	65,30	male	green	good
1732	2011	Lillgrund	L1	51,00	male	green	good
1733	2011	Lillgrund	L1	63,60	male	green	good
1734	2011	Lillgrund	L1	63,60	male	green	reduced
1735	2011	Lillgrund	L1	63,70	male	green	good
1736	2011	Lillgrund	L1	57,30	male	green	good
1737	2011	Lillgrund	L1	54,00	male	green	good
1738	2011	Lillgrund	L1	63,60	male	green	good
1739	2011	Lillgrund	L1	56,50	male	green	reduced
1740	2011	Lillgrund	L1	52,60	male	green	reduced
1741	2011	Lillgrund	L1	51,40	male	green	good
1742	2011	Lillgrund	L1	56,60	male	green	good
1743	2011	Lillgrund	L1	51,40	male	green	good
1744	2011	Lillgrund	L1	48,50	male	green	good
1745	2011	Lillgrund	L1	50,50	male	green	reduced
1746	2011	Lillgrund	L1	60,20	male	green	reduced
1747	2011	Bredgrund	B10	58,30	male	red	good
1748	2011	Bredgrund	B10	67,20	male	green	good
1749	2011	Bredgrund	B10	60,70	male	green	good
1750	2011	Bredgrund	B10	55,20	male	green	good
1751	2011	Bredgrund	B10	65,10	male	green	good
1752	2011	Bredgrund	B10	51,00	male	green	good
1753	2011	Bredgrund	B10	67,50	male	green	good
1754	2011	Bredgrund	B10	62,80	male	green	reduced
1755	2011	Bredgrund	B10	60,20	male	green	reduced
1756	2011	Bredgrund	B10	66,50	male	green	good
1757	2011	Bredgrund	B10	67,50	male	green	reduced
1758	2011	Bredgrund	B10	63,50	male	green	good
1759	2011	Bredgrund	B10	64,90	male	green	good
1760	2011	Bredgrund	B10	55,70	male	green	good
1761	2011	Bredgrund	B10	57,40	male	green	good
1762	2011	Bredgrund	B10	59,60	male	green	reduced
1763	2011	Bredgrund	B10	59,00	male	green	good
1764	2011	Bredgrund	B10	56,70	male	green	good
1765	2011	Bredgrund	B10	49,00	male	green	good
1766	2011	Bredgrund	B10	49,50	male	green	reduced

1767	2011	Bredgrund	B10	48,60	male	green	good
1768	2011	Bredgrund	B9	69,40	male	green	good
1769	2011	Bredgrund	B9	70,10	male	green	good
1770	2011	Bredgrund	B9	52,50	male	red	good
1771	2011	Bredgrund	B9	43,10	female	green	good
1772	2011	Bredgrund	B9	61,10	male	green	good
1773	2011	Bredgrund	B9	60,40	male	green	good
1774	2011	Bredgrund	B9	50,20	male	green	good
1775	2011	Bredgrund	B8	59,10	male	red	good
1776	2011	Bredgrund	B8	57,70	male	green	reduced
1777	2011	Bredgrund	B8	59,90	male	red	reduced
1778	2011	Bredgrund	B8	51,50	male	green	good
1779	2011	Bredgrund	B8	67,70	male	green	good
1780	2011	Bredgrund	B8	63,70	male	green	good
1781	2011	Bredgrund	B8	61,10	male	green	reduced
1782	2011	Bredgrund	B8	56,90	male	green	good
1783	2011	Bredgrund	B8	61,60	male	green	good
1784	2011	Bredgrund	B8	55,90	male	green	good
1785	2011	Bredgrund	B8	53,00	male	green	good
1786	2011	Bredgrund	B8	57,50	male	green	reduced
1787	2011	Bredgrund	B8	61,70	male	green	good
1788	2011	Bredgrund	B8	51,40	male	green	good
1789	2011	Bredgrund	B8	59,10	male	green	good
1790	2011	Bredgrund	B8	60,80	male	green	good
1791	2011	Bredgrund	B8	55,20	male	green	good
1792	2011	Bredgrund	B7	71,80	male	green	good
1793	2011	Bredgrund	B7	60,90	male	green	good
1794	2011	Bredgrund	B7	58,50	male	green	good
1795	2011	Bredgrund	B7	60,20	male	green	good
1796	2011	Bredgrund	B7	61,40	male	green	good
1797	2011	Bredgrund	B7	63,90	male	green	good
1798	2011	Bredgrund	B7	58,70	male	green	good
1799	2011	Bredgrund	B7	53,40	male	green	good
1800	2011	Bredgrund	B7	62,60	male	green	good
1801	2011	Bredgrund	B7	55,50	male	green	good
1802	2011	Bredgrund	B7	59,50	male	green	good
1803	2011	Bredgrund	B7	54,20	male	green	good
1804	2011	Bredgrund	B7	65,30	male	red	good
1805	2011	Bredgrund	B7	58,10	male	green	good
1806	2011	Bredgrund	B7	55,50	male	green	good
1807	2011	Bredgrund	B7	54,40	male	green	reduced
1808	2011	Bredgrund	B7	58,20	male	green	good
1809	2011	Bredgrund	B7	57,30	male	green	good
1810	2011	Bredgrund	B7	47,70	female	red	good
1811	2011	Bredgrund	B7	54,20	male	green	good
1812	2011	Bredgrund	B7	57,10	male	green	good
1813	2011	Bredgrund	B7	59,40	male	green	good
1814	2011	Bredgrund	B7	60,50	male	green	good
1815	2011	Bredgrund	B7	51,40	female	red	good
1816	2011	Bredgrund	B7	54,70	male	red	reduced
1817	2011	Bredgrund	B7	53,80	male	red	good
1818	2011	Bredgrund	B7	48,70	male	green	good
1819	2011	Bredgrund	B7	53,00	male	green	good
1820	2011	Bredgrund	B7	65,70	male	green	good
1821	2011	Bredgrund	B7	60,20	male	green	good
1822	2011	Bredgrund	B7	59,70	male	green	good
1823	2011	Bredgrund	B7	63,30	male	green	good
1824	2011	Bredgrund	B2	60,80	male	green	good
1825	2011	Bredgrund	B2	58,30	male	green	good

1826	2011	Bredgrund	B2	53,10	male	green	good
1827	2011	Bredgrund	B2	57,70	male	green	good
1828	2011	Bredgrund	B5	49,90	male	green	good
1829	2011	Bredgrund	B5	58,90	male	green	good
1830	2011	Bredgrund	B5	58,40	male	red	good
1831	2011	Bredgrund	B5	55,90	male	green	good
1832	2011	Bredgrund	B5	48,60	male	green	good
1833	2011	Bredgrund	B5	54,50	male	green	good
1834	2011	Bredgrund	B5	55,90	male	red	good
1835	2011	Bredgrund	B5	65,20	male	green	good
1836	2011	Bredgrund	B5	68,60	male	green	good
1837	2011	Bredgrund	B5	61,50	male	green	good
1838	2011	Bredgrund	B5	52,00	male	green	good
1839	2011	Bredgrund	B5	48,80	female	red	good
1840	2011	Bredgrund	B4	63,50	male	green	good
1841	2011	Bredgrund	B4	69,80	male	green	reduced
1842	2011	Bredgrund	B4	56,90	male	green	good
1843	2011	Bredgrund	B4	67,40	male	green	good
1844	2011	Bredgrund	B4	70,00	male	green	good
1845	2011	Bredgrund	B4	55,70	male	green	good
1846	2011	Bredgrund	B4	68,50	male	green	good
1847	2011	Bredgrund	B4	63,10	male	green	good
1848	2011	Bredgrund	B4	63,20	male	green	good
1849	2011	Bredgrund	B4	61,20	male	green	good
1850	2011	Bredgrund	B4	51,10	male	red	reduced
1851	2011	Bredgrund	B4	56,20	male	green	good
1852	2011	Bredgrund	B4	53,60	male	green	good
1853	2011	Bredgrund	B4	53,90	male	green	good
1854	2011	Bredgrund	B4	55,20	male	green	good
1855	2011	Bredgrund	B4	51,80	male	green	reduced
1856	2011	Lillgrund	L1	60,60	male	green	good
1857	2011	Lillgrund	L1	63,90	male	green	good
1858	2011	Lillgrund	L1	52,70	male	green	good
1859	2011	Lillgrund	L1	48,70	male	green	good
1860	2011	Lillgrund	L1	50,00	male	green	good
1861	2011	Lillgrund	L1	52,80	male	green	good
1862	2011	Lillgrund	L1	66,40	male	green	good
1863	2011	Lillgrund	L1	51,80	male	green	good
1864	2011	Lillgrund	L1	60,00	male	green	good
1865	2011	Lillgrund	L1	53,50	male	green	good
1866	2011	Lillgrund	L1	59,60	male	green	good
1867	2011	Lillgrund	L1	51,20	male	green	good
1868	2011	Lillgrund	L1	56,50	male	green	good
1869	2011	Lillgrund	L1	50,00	male	green	good
1870	2011	Lillgrund	L1	48,70	male	green	good
1871	2011	Lillgrund	L1	57,20	male	green	good
1872	2011	Lillgrund	L1	50,90	male	green	good
1873	2011	Lillgrund	L1	57,22	male	green	good
1874	2011	Lillgrund	L1	48,60	male	green	good
1875	2011	Lillgrund	L1	46,36	male	green	good
1876	2011	Lillgrund	L1	45,90	male	green	good
1877	2011	Lillgrund	L1	49,78	male	green	good
1878	2011	Lillgrund	L7	66,60	male	green	good
1879	2011	Lillgrund	L7	65,50	male	green	good
1880	2011	Lillgrund	L7	57,90	male	green	good
1881	2011	Lillgrund	L7	58,10	male	green	good
1882	2011	Lillgrund	L7	67,50	male	green	good
1883	2011	Lillgrund	L7	61,05	male	green	good
1884	2011	Lillgrund	L7	61,70	male	green	good

1885	2011	Lillgrund	L7	68,70	male	green	good
1886	2011	Lillgrund	L7	59,00	male	green	good
1887	2011	Lillgrund	L7	72,27	male	green	good
1888	2011	Lillgrund	L7	60,98	male	green	good
1889	2011	Lillgrund	L7	57,59	male	green	good
1890	2011	Lillgrund	L7	66,70	male	green	good
1891	2011	Lillgrund	L7	49,40	male	green	good
1892	2011	Lillgrund	L7	68,16	male	green	good
1893	2011	Lillgrund	L7	49,50	male	green	good
1894	2011	Lillgrund	L7	52,50	male	green	good
1895	2011	Lillgrund	L7	61,10	male	green	good
1896	2011	Lillgrund	L7	59,96	male	green	good
1897	2011	Lillgrund	L7	57,65	male	green	good
1898	2011	Lillgrund	L7	35,10	male	green	good
1899	2011	Lillgrund	L7	64,80	male	green	good
1900	2011	Lillgrund	L7	54,50	male	green	good
1901	2011	Lillgrund	L7	55,90	male	green	good
1902	2011	Lillgrund	L7	53,10	male	green	good
1903	2011	Lillgrund	L7	65,50	male	green	good
1904	2011	Lillgrund	L7	65,80	male	green	good
1905	2011	Lillgrund	L7	46,30	male	green	good
1906	2011	Lillgrund	L7	57,00	male	green	good
1907	2011	Lillgrund	L7	64,60	male	green	good
1908	2011	Lillgrund	L7	56,00	male	green	good
1909	2011	Lillgrund	L7	55,60	male	green	good
1910	2011	Lillgrund	L7	53,80	male	red	good
1911	2011	Lillgrund	L7	46,30	female	red	good
1912	2011	Lillgrund	L7	52,80	male	green	reduced
1913	2011	Lillgrund	L7	54,60	male	green	good
1914	2011	Lillgrund	L7	66,70	male	green	good
1915	2011	Lillgrund	L7	60,70	male	green	good
1916	2011	Lillgrund	L7	48,90	male	green	good
1917	2011	Lillgrund	L7	61,30	male	green	good
1918	2011	Lillgrund	L7	61,50	male	green	good
1919	2011	Lillgrund	L7	54,90	male	green	good
1920	2011	Lillgrund	L7	48,50	female	green	good
1921	2011	Lillgrund	L7	65,60	male	green	good
1922	2011	Lillgrund	L7	60,90	male	green	good
1923	2011	Lillgrund	L7	60,20	male	green	good
1924	2011	Lillgrund	L7	62,80	male	green	good
1925	2011	Lillgrund	L7	57,50	male	green	reduced
1926	2011	Lillgrund	L7	64,40	male	green	good
1927	2011	Lillgrund	L7	66,50	male	green	good
1928	2011	Lillgrund	L10	55,80	male	green	good
1929	2011	Lillgrund	L10	67,70	male	green	good
1930	2011	Lillgrund	L10	69,80	male	green	good
1931	2011	Lillgrund	L10	70,40	male	green	good
1932	2011	Lillgrund	L10	48,60	male	green	good
1933	2011	Lillgrund	L10	49,90	male	green	good
1934	2011	Lillgrund	L10	47,60	male	green	good
1935	2011	Lillgrund	L10	51,60	male	green	good
1936	2011	Lillgrund	L10	62,50	male	green	good
1937	2011	Lillgrund	L10	52,50	male	green	good
1938	2011	Lillgrund	L10	55,00	male	green	good
1939	2011	Lillgrund	L10	44,10	male	green	good
1940	2011	Lillgrund	L10	64,00	male	green	good
1941	2011	Lillgrund	L10	49,40	male	green	good
1942	2011	Lillgrund	L10	49,20	male	green	good
1943	2011	Lillgrund	L10	58,30	male	green	good

1944	2011	Lillgrund	L10	51,80	male	green	good
1945	2011	Lillgrund	L10	63,30	male	green	good
1946	2011	Lillgrund	L10	68,00	male	green	good
1947	2011	Lillgrund	L10	63,00	male	green	good
1948	2011	Lillgrund	L10	64,00	male	green	good
1949	2011	Lillgrund	L10	54,40	male	green	good
1950	2011	Lillgrund	L10	59,60	male	green	good
1951	2011	Lillgrund	L10	55,90	male	green	good
1952	2011	Lillgrund	L10	66,90	male	green	reduced
1953	2011	Lillgrund	L10	71,50	male	green	good
1954	2011	Lillgrund	L10	65,60	female	green	good
1955	2011	Lillgrund	L10	57,90	male	green	good
1956	2011	Lillgrund	L10	48,60	male	green	good
1957	2011	Lillgrund	L10	61,70	male	green	good
1958	2011	Lillgrund	L10	43,50	male	green	good
1959	2011	Lillgrund	L10	53,50	male	green	good
1960	2011	Lillgrund	L10	59,10	male	green	good
1961	2011	Lillgrund	L10	63,00	male	green	good
1962	2011	Lillgrund	L10	49,40	male	green	reduced
1963	2011	Lillgrund	L10	43,70	male	green	good
1964	2011	Lillgrund	L8	78,80	male	green	good
1965	2011	Lillgrund	L8	64,30	male	green	reduced
1966	2011	Lillgrund	L8	58,30	male	green	good
1967	2011	Lillgrund	L8	66,90	male	green	good
1968	2011	Lillgrund	L8	65,50	male	green	good
1969	2011	Lillgrund	L8	68,80	male	green	good
1970	2011	Lillgrund	L8	68,00	male	red	good
1971	2011	Lillgrund	L8	61,90	male	green	good
1972	2011	Lillgrund	L8	61,40	male	green	good
1973	2011	Lillgrund	L8	65,80	male	green	good
1974	2011	Lillgrund	L8	64,50	male	green	good
1975	2011	Lillgrund	L8	61,30	male	green	reduced
1976	2011	Lillgrund	L8	61,40	male	green	reduced
1977	2011	Lillgrund	L8	61,70	male	green	good
1978	2011	Lillgrund	L8	49,90	male	green	good
1979	2011	Lillgrund	L8	59,60	male	green	good
1980	2011	Lillgrund	L8	62,60	male	green	good
1981	2011	Lillgrund	L8	64,70	male	green	good
1982	2011	Lillgrund	L8	61,60	male	green	good
1983	2011	Lillgrund	L8	67,50	male	green	good
1984	2011	Lillgrund	L8	46,00	female	green	good
1985	2011	Lillgrund	L8	60,10	male	green	good
1986	2011	Lillgrund	L8	48,60	male	green	good
1987	2011	Lillgrund	L8	55,60	male	green	good
1988	2011	Lillgrund	L6	61,10	male	green	good
1989	2011	Lillgrund	L6	63,50	male	green	reduced
1990	2011	Lillgrund	L6	61,90	male	green	reduced
1991	2011	Lillgrund	L6	55,50	male	green	reduced
1992	2011	Lillgrund	L6	57,30	male	green	good
1993	2011	Lillgrund	L6	71,20	male	green	good
1994	2011	Lillgrund	L6	66,80	male	green	good
1995	2011	Lillgrund	L6	53,60	male	green	good
1996	2011	Lillgrund	L6	59,40	male	green	good
1997	2011	Lillgrund	L6	43,00	male	green	good
1998	2011	Lillgrund	L6	67,90	male	green	good
1999	2011	Lillgrund	L6	69,60	male	green	good
2000	2011	Lillgrund	L6	61,70	male	green	good
2001	2011	Lillgrund	L6	60,90	male	green	good
2002	2011	Lillgrund	L6	65,00	male	green	good

2003	2011	Lillgrund	L6	69,20	male	green	good
2004	2011	Lillgrund	L6	57,90	male	red	good
2005	2011	Lillgrund	L6	64,50	male	green	good
2006	2011	Lillgrund	L6	57,70	male	green	good
2007	2011	Lillgrund	L6	62,00	male	green	good
2008	2011	Lillgrund	L6	48,00	male	green	good
2009	2011	Lillgrund	L6	59,00	male	green	good
2010	2011	Lillgrund	L6	52,50	male	green	good
2011	2011	Lillgrund	L6	58,70	male	green	good
2012	2011	Lillgrund	L6	49,20	male	green	good
2013	2011	Lillgrund	L6	50,90	male	green	good
2014	2011	Lillgrund	L6	49,10	male	green	good
2015	2011	Lillgrund	L6	50,90	male	green	good
2016	2011	Lillgrund	L6	59,50	male	green	good
2017	2011	Lillgrund	L6	70,80	male	green	good
2018	2011	Lillgrund	L6	52,30	male	green	reduced
2019	2011	Lillgrund	L6	64,60	male	green	reduced
2020	2011	Lillgrund	L6	63,20	male	green	good
2021	2011	Lillgrund	L6	55,90	male	green	good
2022	2011	Lillgrund	L6	52,20	male	green	good
2023	2011	Lillgrund	L6	65,50	male	green	good
2024	2011	Lillgrund	L6	52,40	male	green	good
2025	2011	Lillgrund	L6	45,70	male	green	good
2026	2011	Lillgrund	L6	53,30	male	green	good
2027	2011	Lillgrund	L6	65,10	male	green	good
2028	2011	Lillgrund	L6	45,60	female	green	good
2029	2011	Lillgrund	L3	67,30	male	green	good
2030	2011	Lillgrund	L3	71,20	male	green	good
2031	2011	Lillgrund	L3	71,70	male	green	good
2032	2011	Lillgrund	L3	59,40	male	green	good
2033	2011	Lillgrund	L3	57,20	male	green	good
2034	2011	Lillgrund	L3	59,60	male	green	good
2035	2011	Lillgrund	L3	65,90	male	green	good
2036	2011	Lillgrund	L3	56,80	male	green	good
2037	2011	Lillgrund	L3	67,30	male	green	good
2038	2011	Lillgrund	L3	51,10	male	red	good
2039	2011	Lillgrund	L3	66,00	male	green	good
2040	2011	Lillgrund	L3	64,80	male	green	reduced
2041	2011	Lillgrund	L3	60,50	male	green	good
2042	2011	Lillgrund	L3	66,40	male	green	good
2043	2011	Lillgrund	L3	73,40	male	green	good
2044	2011	Lillgrund	L3	66,80	male	green	good
2045	2011	Lillgrund	L3	65,60	male	green	good
2046	2011	Lillgrund	L3	63,00	male	green	good
2047	2011	Lillgrund	L3	65,00	male	green	good
2048	2011	Lillgrund	L3	54,10	male	green	good
2049	2011	Lillgrund	L3	64,00	male	green	good
2050	2011	Lillgrund	L3	55,20	male	green	good
2051	2011	Lillgrund	L3	54,70	male	red	good
2052	2011	Lillgrund	L3	54,80	male	green	good
2053	2011	Lillgrund	L3	57,70	male	green	good
2054	2011	Lillgrund	L3	53,20	male	green	good
2055	2011	Lillgrund	L3	57,90	male	green	good
2056	2011	Lillgrund	L3	56,20	male	green	good
2057	2011	Lillgrund	L3	60,30	male	green	good
2058	2011	Lillgrund	L3	50,90	male	green	good
2059	2011	Lillgrund	L3	61,40	male	green	good
2060	2011	Lillgrund	L3	52,80	male	green	good
2061	2011	Lillgrund	L3	61,60	male	green	good

2062	2011	Lillgrund	L3	53,00	male	green	good
2063	2011	Lillgrund	L3	51,70	male	green	good
2064	2011	Lillgrund	L3	46,30	male	green	reduced
2065	2011	Lillgrund	L3	54,70	male	green	good
2066	2011	Lillgrund	L3	51,30	male	green	good
2067	2011	Lillgrund	L3	60,60	male	red	good
2068	2011	Lillgrund	L3	50,20	male	green	good
2069	2011	Lillgrund	L3	59,00	male	green	good
2070	2011	Lillgrund	L3	55,90	male	red	good
2071	2011	Lillgrund	L3	52,40	male	green	good
2072	2011	Lillgrund	L3	49,30	male	green	good
2073	2011	Lillgrund	L3	65,90	male	green	good
2074	2011	Lillgrund	L3	60,70	male	green	good
2075	2011	Lillgrund	L3	46,90	male	green	good
2076	2011	Lillgrund	L3	60,00	male	green	good
2077	2011	Lillgrund	L3	46,70	male	green	good
2078	2011	Lillgrund	L3	47,00	male	green	good
2079	2011	Lillgrund	L9	70,80	male	green	good
2080	2011	Lillgrund	L9	68,50	male	green	reduced
2081	2011	Lillgrund	L9	50,00	male	green	good
2082	2011	Lillgrund	L9	61,30	male	green	good
2083	2011	Lillgrund	L9	71,20	male	green	good
2084	2011	Lillgrund	L9	50,70	male	green	good
2085	2011	Lillgrund	L9	63,00	male	green	good
2086	2011	Lillgrund	L9	55,30	male	green	reduced
2087	2011	Lillgrund	L9	62,90	male	green	good
2088	2011	Lillgrund	L9	69,90	male	green	good
2089	2011	Lillgrund	L9	57,60	male	green	good
2090	2011	Lillgrund	L9	66,90	male	green	good
2091	2011	Lillgrund	L9	55,30	male	green	good
2092	2011	Lillgrund	L9	65,50	male	green	good
2093	2011	Lillgrund	L9	54,50	male	green	good
2094	2011	Lillgrund	L9	60,30	male	green	good
2095	2011	Lillgrund	L9	52,70	male	green	good
2096	2011	Lillgrund	L9	58,80	male	green	good
2097	2011	Lillgrund	L9	68,60	male	red	good
2098	2011	Lillgrund	L9	63,60	male	green	good
2099	2011	Lillgrund	L9	62,90	male	green	reduced
2100	2011	Lillgrund	L9	64,60	male	green	reduced
2101	2011	Lillgrund	L9	60,20	male	green	good
2102	2011	Lillgrund	L9	45,50	male	green	good
2103	2011	Lillgrund	L9	62,60	male	green	good
2104	2011	Lillgrund	L9	62,80	male	green	good
2105	2011	Lillgrund	L9	59,40	male	green	good
2106	2011	Lillgrund	L9	53,00	male	green	good
2107	2011	Lillgrund	L9	53,80	male	green	good
2108	2011	Lillgrund	L9	57,00	male	green	good
2109	2011	Lillgrund	L9	55,50	male	green	good
2110	2011	Lillgrund	L9	50,80	male	green	good
2111	2011	Lillgrund	L9	50,10	male	green	good
2112	2011	Lillgrund	L9	63,20	male	green	good
2113	2011	Lillgrund	L9	53,00	male	green	good
2114	2011	Lillgrund	L9	50,70	male	green	reduced
2115	2011	Lillgrund	L9	47,50	male	green	good
2116	2011	Lillgrund	L5	58,50	male	green	reduced
2117	2011	Lillgrund	L5	55,00	male	green	good
2118	2011	Lillgrund	L5	60,50	male	green	good
2119	2011	Bredgrund	B4	58,90	male	green	good
2120	2011	Bredgrund	B4	50,00	male	green	good

2121	2011	Bredgrund	B4	58,40	male	green	reduced
2122	2011	Bredgrund	B1	63,00	male	green	good
2123	2011	Bredgrund	B1	57,10	male	green	good
2124	2011	Bredgrund	B1	49,30	male	green	good
2125	2011	Bredgrund	B1	57,10	male	green	good
2126	2011	Bredgrund	B1	63,00	male	green	good
2127	2011	Bredgrund	B1	56,20	male	green	good
2128	2011	Bredgrund	B1	60,90	male	green	good
2129	2011	Bredgrund	B1	60,60	male	green	good
2130	2011	Bredgrund	B1	52,70	male	green	reduced
2131	2011	Bredgrund	B1	60,90	male	green	reduced
2132	2011	Bredgrund	B1	59,20	male	green	reduced
2133	2011	Bredgrund	B1	61,70	male	green	good
2134	2011	Bredgrund	B3	63,90	male	green	good
2135	2011	Bredgrund	B3	63,80	male	green	good
2136	2011	Bredgrund	B3	65,12	male	green	good
2137	2011	Bredgrund	B3	65,80	male	green	good
2138	2011	Bredgrund	B3	69,40	male	green	good
2139	2011	Bredgrund	B3	53,30	male	green	good
2140	2011	Bredgrund	B3	59,50	male	green	good
2141	2011	Bredgrund	B3	58,50	male	green	good
2142	2011	Bredgrund	B3	56,60	male	green	good
2143	2011	Bredgrund	B3	55,50	male	green	good
2144	2011	Bredgrund	B3	58,70	male	green	good
2145	2011	Bredgrund	B3	62,60	male	green	good
2146	2011	Bredgrund	B3	49,70	female	green	good
2147	2011	Bredgrund	B3	58,70	male	green	good
2148	2011	Bredgrund	B6	45,30	male	red	good
2149	2011	Bredgrund	B6	68,50	male	green	good
2150	2011	Bredgrund	B6	47,50	male	green	good
2151	2011	Bredgrund	B6	61,30	male	green	good
2152	2011	Bredgrund	B6	63,50	male	green	good
2153	2011	Bredgrund	B6	60,90	male	green	reduced
2154	2011	Bredgrund	B6	59,50	male	green	good
2155	2011	Bredgrund	B6	70,60	male	green	reduced
2156	2011	Bredgrund	B6	64,00	male	green	good
2157	2011	Bredgrund	B6	43,20	male	green	reduced
2158	2011	Bredgrund	B6	64,40	male	green	reduced
2159	2011	Bredgrund	B6	52,00	male	green	good
2160	2011	Bredgrund	B6	58,50	male	green	good
2161	2011	Bredgrund	B6	54,50	male	green	good
2162	2011	Bredgrund	B6	40,00	male	green	good
2163	2011	Bredgrund	B6	47,10	female	green	good
2164	2011	Bredgrund	B6	64,10	male	green	good
2165	2011	Bredgrund	B6	53,70	male	green	good
2166	2011	Bredgrund	B6	59,30	male	green	good
2167	2011	Bredgrund	B6	46,60	female	green	good
2168	2011	Bredgrund	B6	50,90	male	green	good
2169	2011	Bredgrund	B6	51,70	male	green	good
2170	2011	Bredgrund	B6	50,80	male	green	good
2171	2011	Bredgrund	B6	61,40	male	green	good
2172	2011	Bredgrund	B6	66,70	male	green	good
2173	2011	Bredgrund	B6	49,10	male	green	good
2174	2011	Bredgrund	B6	60,90	male	green	good
2175	2011	Bredgrund	B6	55,20	male	green	good
2176	2011	Bredgrund	B6	56,70	male	green	good
2177	2011	Bredgrund	B6	50,70	male	green	good
2178	2012	Sjollen	S1	46,10	male	green	good
2179	2012	Sjollen	S1	51,80	male	red	good

2180	2012	Sjollen	S1	52,10	male	red	good
2181	2012	Sjollen	S1	49,20	male	green	good
2182	2012	Sjollen	S1	55,20	male	red	good
2183	2012	Sjollen	S1	55,90	male	red	good
2184	2012	Sjollen	S1	42,30	male	green	good
2185	2012	Sjollen	S1	44,20	male	green	good
2186	2012	Sjollen	S1	53,60	male	green	good
2187	2012	Sjollen	S1	55,50	male	green	good
2188	2012	Sjollen	S1	58,20	male	green	good
2189	2012	Sjollen	S1	58,20	male	green	good
2190	2012	Sjollen	S1	58,60	male	green	good
2191	2012	Sjollen	S1	58,90	male	green	good
2192	2012	Sjollen	S1	61,00	male	green	good
2193	2012	Sjollen	S1	61,70	male	green	good
2194	2012	Sjollen	S1	61,70	male	green	good
2195	2012	Sjollen	S1	62,50	male	green	good
2196	2012	Sjollen	S1	62,50	male	green	good
2197	2012	Sjollen	S1	62,90	male	green	good
2198	2012	Sjollen	S1	64,00	male	green	good
2199	2012	Sjollen	S1	64,40	male	green	good
2200	2012	Sjollen	S1	65,20	male	green	reduced
2201	2012	Sjollen	S1	67,00	male	green	good
2202	2012	Sjollen	S1	67,80	male	green	good
2203	2012	Sjollen	S1	69,60	male	green	good
2204	2012	Sjollen	S1	73,80	male	green	good
2205	2012	Sjollen	S1	39,80	female	red	reduced
2206	2012	Sjollen	S1	57,90	male	red	good
2207	2012	Sjollen	S1	55,50	male	green	reduced
2208	2012	Sjollen	S1	56,30	male	green	reduced
2209	2012	Sjollen	S1	57,40	male	green	good
2210	2012	Sjollen	S1	57,50	male	green	good
2211	2012	Sjollen	S1	57,80	male	green	reduced
2212	2012	Sjollen	S1	58,20	male	green	good
2213	2012	Sjollen	S1	58,90	male	green	good
2214	2012	Sjollen	S1	60,80	male	green	good
2215	2012	Sjollen	S1	62,10	male	green	good
2216	2012	Sjollen	S1	63,60	male	green	good
2217	2012	Sjollen	S1	63,70	male	green	good
2218	2012	Sjollen	S1	55,40	male	red	good
2219	2012	Sjollen	S1	55,90	male	red	good
2220	2012	Sjollen	S1	58,40	male	red	reduced
2221	2012	Sjollen	S1	59,10	male	red	reduced
2222	2012	Sjollen	S1	59,60	male	red	good
2223	2012	Sjollen	S1	54,00	male	red	good
2224	2012	Sjollen	S10	50,60	male	green	good
2225	2012	Sjollen	S10	55,90	male	green	good
2226	2012	Sjollen	S10	56,70	male	green	good
2227	2012	Sjollen	S10	57,90	male	green	good
2228	2012	Sjollen	S10	61,00	male	green	reduced
2229	2012	Sjollen	S10	44,50	male	red	good
2230	2012	Sjollen	S10	45,50	male	red	good
2231	2012	Sjollen	S10	52,80	male	red	good
2232	2012	Sjollen	S10	53,20	male	red	good
2233	2012	Sjollen	S10	53,90	male	red	good
2234	2012	Sjollen	S10	56,90	male	red	reduced
2235	2012	Sjollen	S10	57,40	male	red	good
2236	2012	Sjollen	S10	57,90	male	red	good
2237	2012	Sjollen	S10	58,70	male	red	good
2238	2012	Sjollen	S10	66,10	male	green	good

2239	2012	Sjollen	S10	66,20	male	green	good
2240	2012	Sjollen	S10	50,10	male	red	good
2241	2012	Sjollen	S10	51,90	male	red	good
2242	2012	Sjollen	S10	53,30	male	red	reduced
2243	2012	Sjollen	S10	54,10	male	red	reduced
2244	2012	Sjollen	S10	54,40	male	red	reduced
2245	2012	Sjollen	S10	55,00	male	red	good
2246	2012	Sjollen	S10	56,50	male	red	good
2247	2012	Sjollen	S10	56,50	male	red	good
2248	2012	Sjollen	S10	57,00	male	red	reduced
2249	2012	Sjollen	S10	57,30	male	red	good
2250	2012	Sjollen	S10	58,50	male	red	good
2251	2012	Sjollen	S10	60,30	male	red	good
2252	2012	Sjollen	S10	61,70	male	red	good
2253	2012	Sjollen	S10	61,70	male	red	good
2254	2012	Sjollen	S10	62,30	male	red	reduced
2255	2012	Sjollen	S10	57,10	male	green	good
2256	2012	Sjollen	S10	66,70	male	green	reduced
2257	2012	Sjollen	S10	68,80	male	green	good
2258	2012	Sjollen	S10	53,00	male	red	reduced
2259	2012	Sjollen	S10	60,40	male	red	reduced
2260	2012	Sjollen	S10	61,20	male	red	good
2261	2012	Sjollen	S10	40,10	female	red	good
2262	2012	Sjollen	S10	54,20	male	red	good
2263	2012	Sjollen	S10	56,10	male	red	reduced
2264	2012	Sjollen	S10	57,70	male	red	good
2265	2012	Sjollen	S10	58,20	male	red	good
2266	2012	Sjollen	S10	45,00	male	green	good
2267	2012	Sjollen	S10	50,80	male	green	reduced
2268	2012	Sjollen	S10	53,10	male	green	good
2269	2012	Sjollen	S10	53,50	male	green	good
2270	2012	Sjollen	S10	58,40	male	green	reduced
2271	2012	Sjollen	S10	58,50	male	green	good
2272	2012	Sjollen	S10	60,60	male	green	good
2273	2012	Sjollen	S10	61,30	male	green	good
2274	2012	Sjollen	S10	63,80	male	green	good
2275	2012	Sjollen	S10	68,40	male	green	good
2276	2012	Sjollen	S10	71,60	male	green	good
2277	2012	Sjollen	S10	48,50	female	red	good
2278	2012	Sjollen	S10	53,30	male	red	reduced
2279	2012	Sjollen	S10	55,80	male	red	good
2280	2012	Sjollen	S10	58,90	male	red	good
2281	2012	Sjollen	S10	59,20	male	red	good
2282	2012	Sjollen	S10	63,40	male	red	good
2283	2012	Sjollen	S10	65,60	male	red	good
2284	2012	Sjollen	S10	66,30	male	red	good
2285	2012	Sjollen	S10	35,50	male	green	good
2286	2012	Sjollen	S10	52,20	male	green	good
2287	2012	Sjollen	S10	58,20	male	green	reduced
2288	2012	Sjollen	S10	59,90	male	green	good
2289	2012	Sjollen	S10	67,80	male	green	good
2290	2012	Sjollen	S10	45,90	male	red	good
2291	2012	Sjollen	S10	54,20	male	red	good
2292	2012	Sjollen	S10	55,50	male	red	good
2293	2012	Sjollen	S10	56,80	male	red	good
2294	2012	Sjollen	S10	57,10	male	red	reduced
2295	2012	Sjollen	S10	57,20	male	red	reduced
2296	2012	Sjollen	S10	58,60	male	red	good
2297	2012	Sjollen	S10	60,20	male	red	good

2298	2012	Sjollen	S10	60,80	male	red	good
2299	2012	Sjollen	S10	61,70	male	red	good
2300	2012	Sjollen	S10	63,40	male	red	good
2301	2012	Sjollen	S10	68,20	male	red	good
2302	2012	Sjollen	S2	60,50	male	green	reduced
2303	2012	Sjollen	S2	63,20	male	green	good
2304	2012	Sjollen	S2	44,10	male	red	good
2305	2012	Sjollen	S2	48,90	female	red	good
2306	2012	Sjollen	S2	49,50	male	red	good
2307	2012	Sjollen	S2	55,30	male	red	good
2308	2012	Sjollen	S2	56,50	male	red	good
2309	2012	Sjollen	S2	56,70	male	red	good
2310	2012	Sjollen	S2	56,80	male	red	good
2311	2012	Sjollen	S2	57,30	male	red	good
2312	2012	Sjollen	S2	57,80	male	red	reduced
2313	2012	Sjollen	S2	58,30	male	red	good
2314	2012	Sjollen	S2	68,30	male	red	good
2315	2012	Sjollen	S2	42,50	male	green	good
2316	2012	Sjollen	S2	45,70	male	green	good
2317	2012	Sjollen	S2	52,40	male	red	good
2318	2012	Sjollen	S2	53,40	male	red	reduced
2319	2012	Sjollen	S2	54,70	male	red	good
2320	2012	Sjollen	S2	58,40	male	red	good
2321	2012	Sjollen	S2	62,60	male	green	good
2322	2012	Sjollen	S2	46,40	male	red	good
2323	2012	Sjollen	S2	49,80	male	red	good
2324	2012	Sjollen	S2	63,80	male	green	good
2325	2012	Sjollen	S2	64,70	male	red	good
2326	2012	Sjollen	S2	42,70	male	green	good
2327	2012	Sjollen	S2	45,70	male	green	reduced
2328	2012	Sjollen	S2	47,90	male	green	good
2329	2012	Sjollen	S2	52,10	male	green	good
2330	2012	Sjollen	S2	52,90	male	green	good
2331	2012	Sjollen	S2	53,00	male	green	good
2332	2012	Sjollen	S2	54,30	male	green	good
2333	2012	Sjollen	S2	56,40	male	green	good
2334	2012	Sjollen	S2	56,40	male	green	good
2335	2012	Sjollen	S2	56,40	male	green	good
2336	2012	Sjollen	S2	56,80	male	green	good
2337	2012	Sjollen	S2	58,60	male	green	good
2338	2012	Sjollen	S2	59,00	male	green	reduced
2339	2012	Sjollen	S2	59,50	male	green	good
2340	2012	Sjollen	S2	60,00	male	green	good
2341	2012	Sjollen	S2	60,50	male	green	good
2342	2012	Sjollen	S2	60,70	male	green	good
2343	2012	Sjollen	S2	60,90	male	green	reduced
2344	2012	Sjollen	S2	61,50	male	green	good
2345	2012	Sjollen	S2	61,70	male	green	good
2346	2012	Sjollen	S2	62,80	male	green	reduced
2347	2012	Sjollen	S2	67,30	male	green	good
2348	2012	Sjollen	S2	67,70	male	green	good
2349	2012	Sjollen	S2	68,20	male	green	good
2350	2012	Sjollen	S2	71,10	male	green	reduced
2351	2012	Sjollen	S2	40,10	female	red	good
2352	2012	Sjollen	S2	52,90	male	red	good
2353	2012	Sjollen	S2	53,70	male	red	good
2354	2012	Sjollen	S2	53,90	male	red	good
2355	2012	Sjollen	S2	55,20	male	red	reduced
2356	2012	Sjollen	S2	55,80	male	red	good

2357	2012	Sjollen	S2	58,40	male	red	reduced
2358	2012	Sjollen	S2	58,70	male	red	good
2359	2012	Sjollen	S2	59,20	male	red	good
2360	2012	Sjollen	S2	60,00	male	red	good
2361	2012	Sjollen	S2	60,50	male	red	reduced
2362	2012	Sjollen	S2	63,40	male	red	good
2363	2012	Sjollen	S2	64,90	male	red	good
2364	2012	Sjollen	S2	65,70	male	red	good
2365	2012	Sjollen	S2	67,10	male	red	good
2366	2012	Sjollen	S2	67,40	male	red	good
2367	2012	Sjollen	S2	42,60	female	green	good
2368	2012	Sjollen	S2	43,70	male	green	good
2369	2012	Sjollen	S2	45,00	male	green	good
2370	2012	Sjollen	S2	48,30	male	green	good
2371	2012	Sjollen	S2	53,20	male	green	good
2372	2012	Sjollen	S2	54,20	male	green	reduced
2373	2012	Sjollen	S2	54,50	male	green	good
2374	2012	Sjollen	S2	54,90	male	green	good
2375	2012	Sjollen	S2	55,20	male	green	good
2376	2012	Sjollen	S2	55,80	male	green	reduced
2377	2012	Sjollen	S2	56,80	male	green	good
2378	2012	Sjollen	S2	57,20	male	green	good
2379	2012	Sjollen	S2	59,70	male	green	good
2380	2012	Sjollen	S2	60,00	male	green	good
2381	2012	Sjollen	S2	60,10	male	green	good
2382	2012	Sjollen	S2	60,30	male	green	good
2383	2012	Sjollen	S2	62,50	male	green	good
2384	2012	Sjollen	S2	63,10	male	green	good
2385	2012	Sjollen	S2	63,50	male	green	good
2386	2012	Sjollen	S2	65,20	male	green	good
2387	2012	Sjollen	S2	28,90	male	red	good
2388	2012	Sjollen	S2	36,10	female	red	reduced
2389	2012	Sjollen	S2	48,10	male	red	good
2390	2012	Sjollen	S2	51,40	female	red	good
2391	2012	Sjollen	S2	53,50	male	red	good
2392	2012	Sjollen	S2	58,00	male	red	good
2393	2012	Sjollen	S2	58,50	male	red	good
2394	2012	Sjollen	S2	58,50	male	red	good
2395	2012	Sjollen	S2	59,00	male	red	good
2396	2012	Sjollen	S2	59,80	male	red	good
2397	2012	Sjollen	S2	60,90	male	red	reduced
2398	2012	Sjollen	S2	61,20	male	red	good
2399	2012	Sjollen	S2	68,70	male	red	good
2400	2012	Sjollen	S3	43,90	female	red	reduced
2401	2012	Sjollen	S3	51,70	male	red	good
2402	2012	Sjollen	S3	53,40	male	red	good
2403	2012	Sjollen	S3	53,70	male	red	good
2404	2012	Sjollen	S3	54,40	male	red	good
2405	2012	Sjollen	S3	59,10	male	red	good
2406	2012	Sjollen	S3	59,10	male	red	good
2407	2012	Sjollen	S3	59,50	male	red	reduced
2408	2012	Sjollen	S3	60,50	male	red	good
2409	2012	Sjollen	S3	62,10	male	red	good
2410	2012	Sjollen	S3	62,10	male	red	good
2411	2012	Sjollen	S3	62,10	male	red	good
2412	2012	Sjollen	S3	62,70	male	red	good
2413	2012	Sjollen	S3	64,50	male	red	good
2414	2012	Sjollen	S3	40,40	male	green	good
2415	2012	Sjollen	S3	48,10	male	green	good

2416	2012	Sjollen	S3	50,70	male	green	good
2417	2012	Sjollen	S3	52,50	male	green	good
2418	2012	Sjollen	S3	57,10	male	green	reduced
2419	2012	Sjollen	S3	60,50	male	green	good
2420	2012	Sjollen	S3	66,70	male	green	good
2421	2012	Sjollen	S3	51,70	male	red	reduced
2422	2012	Sjollen	S3	54,40	male	red	good
2423	2012	Sjollen	S3	55,30	male	red	good
2424	2012	Sjollen	S3	56,20	male	red	reduced
2425	2012	Sjollen	S3	57,30	male	red	good
2426	2012	Sjollen	S3	57,70	male	red	good
2427	2012	Sjollen	S3	57,90	male	red	good
2428	2012	Sjollen	S3	59,90	male	red	good
2429	2012	Sjollen	S3	60,20	male	green	good
2430	2012	Sjollen	S3	69,50	male	green	reduced
2431	2012	Sjollen	S3	39,90	female	red	good
2432	2012	Sjollen	S3	49,70	male	red	good
2433	2012	Sjollen	S3	52,40	male	red	good
2434	2012	Sjollen	S3	55,10	male	red	reduced
2435	2012	Sjollen	S3	56,20	male	red	reduced
2436	2012	Sjollen	S3	57,40	male	red	good
2437	2012	Sjollen	S3	58,30	male	red	good
2438	2012	Sjollen	S3	55,80	male	green	good
2439	2012	Sjollen	S3	45,10	female	red	good
2440	2012	Sjollen	S3	54,30	male	red	good
2441	2012	Sjollen	S3	60,50	male	red	good
2442	2012	Sjollen	S3	73,70	male	red	good
2443	2012	Sjollen	S3	46,20	male	green	good
2444	2012	Sjollen	S3	48,00	male	green	good
2445	2012	Sjollen	S3	52,00	male	green	reduced
2446	2012	Sjollen	S3	56,50	male	green	good
2447	2012	Sjollen	S3	58,30	male	green	good
2448	2012	Sjollen	S3	58,60	male	green	reduced
2449	2012	Sjollen	S3	58,70	male	green	good
2450	2012	Sjollen	S3	59,00	male	green	good
2451	2012	Sjollen	S3	62,30	male	green	good
2452	2012	Sjollen	S3	62,40	male	green	good
2453	2012	Sjollen	S3	66,00	male	green	good
2454	2012	Sjollen	S3	66,30	male	green	good
2455	2012	Sjollen	S3	66,70	male	green	reduced
2456	2012	Sjollen	S3	67,10	male	green	good
2457	2012	Sjollen	S3	38,40	female	red	good
2458	2012	Sjollen	S3	41,60	female	red	good
2459	2012	Sjollen	S3	42,60	female	red	good
2460	2012	Sjollen	S3	45,70	male	red	good
2461	2012	Sjollen	S3	58,40	male	red	good
2462	2012	Sjollen	S3	60,50	male	red	good
2463	2012	Sjollen	S3	65,90	male	red	good
2464	2012	Sjollen	S3	69,50	male	red	good
2465	2012	Sjollen	S3	38,70	male	green	good
2466	2012	Sjollen	S3	43,60	male	green	good
2467	2012	Sjollen	S3	45,30	male	green	reduced
2468	2012	Sjollen	S3	49,00	male	green	good
2469	2012	Sjollen	S3	53,80	male	green	good
2470	2012	Sjollen	S3	55,70	male	green	reduced
2471	2012	Sjollen	S3	56,70	male	green	good
2472	2012	Sjollen	S3	58,20	male	green	good
2473	2012	Sjollen	S3	58,90	male	green	good
2474	2012	Sjollen	S3	59,90	male	green	good

2475	2012	Sjollen	S3	60,40	male	green	good
2476	2012	Sjollen	S3	61,00	male	green	good
2477	2012	Sjollen	S3	61,10	male	green	good
2478	2012	Sjollen	S3	61,60	male	green	good
2479	2012	Sjollen	S3	63,10	male	green	good
2480	2012	Sjollen	S3	63,50	male	green	good
2481	2012	Sjollen	S3	64,50	male	green	reduced
2482	2012	Sjollen	S3	67,10	male	green	good
2483	2012	Sjollen	S3	68,00	male	green	good
2484	2012	Sjollen	S3	59,80	male	red	good
2485	2012	Sjollen	S3	61,40	male	red	good
2486	2012	Sjollen	S3	62,80	male	red	reduced
2487	2012	Sjollen	S3	64,20	male	red	good
2488	2012	Sjollen	S3	66,40	male	red	good
2489	2012	Sjollen	S4	49,10	male	red	reduced
2490	2012	Sjollen	S4	54,50	male	red	good
2491	2012	Sjollen	S4	58,30	male	red	good
2492	2012	Sjollen	S4	58,80	male	red	good
2493	2012	Sjollen	S4	53,30	male	red	reduced
2494	2012	Sjollen	S4	57,50	male	red	good
2495	2012	Sjollen	S4	57,70	male	red	good
2496	2012	Sjollen	S4	61,70	male	green	reduced
2497	2012	Sjollen	S4	58,40	male	red	good
2498	2012	Sjollen	S4	41,80	male	green	good
2499	2012	Sjollen	S4	42,00	female	green	good
2500	2012	Sjollen	S4	47,30	male	green	good
2501	2012	Sjollen	S4	55,40	male	green	good
2502	2012	Sjollen	S4	56,80	male	green	good
2503	2012	Sjollen	S4	62,10	male	green	reduced
2504	2012	Sjollen	S4	64,60	male	green	good
2505	2012	Sjollen	S4	65,20	male	green	reduced
2506	2012	Sjollen	S4	65,90	male	green	reduced
2507	2012	Sjollen	S4	70,20	male	green	good
2508	2012	Sjollen	S4	48,80	male	red	reduced
2509	2012	Sjollen	S4	59,90	male	red	good
2510	2012	Sjollen	S4	43,80	male	green	good
2511	2012	Sjollen	S4	43,80	male	green	good
2512	2012	Sjollen	S4	45,20	male	green	good
2513	2012	Sjollen	S4	49,20	male	green	good
2514	2012	Sjollen	S4	53,70	male	green	good
2515	2012	Sjollen	S4	57,20	male	green	good
2516	2012	Sjollen	S4	59,70	male	green	good
2517	2012	Sjollen	S4	63,40	male	green	good
2518	2012	Sjollen	S4	55,30	male	red	good
2519	2012	Sjollen	S4	57,60	male	red	good
2520	2012	Sjollen	S4	60,60	male	red	good
2521	2012	Sjollen	S5	46,00	male	green	good
2522	2012	Sjollen	S5	35,60	male	red	good
2523	2012	Sjollen	S5	46,40	male	red	good
2524	2012	Sjollen	S5	52,80	male	red	good
2525	2012	Sjollen	S5	54,10	male	red	good
2526	2012	Sjollen	S5	55,20	male	red	good
2527	2012	Sjollen	S5	55,60	male	red	reduced
2528	2012	Sjollen	S5	59,00	male	red	reduced
2529	2012	Sjollen	S5	59,60	male	red	good
2530	2012	Sjollen	S5	60,00	male	red	good
2531	2012	Sjollen	S5	62,40	male	red	good
2532	2012	Sjollen	S5	54,90	male	green	good
2533	2012	Sjollen	S5	59,20	male	green	reduced

2534	2012	Sjollen	S5	66,70	male	green	good
2535	2012	Sjollen	S5	69,40	male	green	good
2536	2012	Sjollen	S5	52,60	male	red	good
2537	2012	Sjollen	S5	52,70	male	red	good
2538	2012	Sjollen	S5	53,50	male	red	good
2539	2012	Sjollen	S5	58,70	male	red	good
2540	2012	Sjollen	S5	46,80	male	green	good
2541	2012	Sjollen	S5	56,30	male	green	good
2542	2012	Sjollen	S5	58,00	male	green	good
2543	2012	Sjollen	S5	63,00	male	green	reduced
2544	2012	Sjollen	S5	65,50	male	green	good
2545	2012	Sjollen	S5	68,00	male	green	reduced
2546	2012	Sjollen	S5	69,50	male	green	good
2547	2012	Sjollen	S5	52,00	male	red	good
2548	2012	Sjollen	S5	40,10	female	red	good
2549	2012	Sjollen	S5	49,90	male	red	good
2550	2012	Sjollen	S5	63,50	male	red	good
2551	2012	Sjollen	S5	43,30	male	green	good
2552	2012	Sjollen	S5	44,90	male	green	good
2553	2012	Sjollen	S5	50,10	male	green	good
2554	2012	Sjollen	S5	53,70	male	green	good
2555	2012	Sjollen	S5	54,80	male	green	good
2556	2012	Sjollen	S5	56,00	male	green	good
2557	2012	Sjollen	S5	56,10	male	green	good
2558	2012	Sjollen	S5	56,70	male	green	good
2559	2012	Sjollen	S5	57,00	male	green	good
2560	2012	Sjollen	S5	58,90	male	green	good
2561	2012	Sjollen	S5	59,00	male	green	reduced
2562	2012	Sjollen	S5	59,10	male	green	good
2563	2012	Sjollen	S5	59,90	male	green	reduced
2564	2012	Sjollen	S5	60,00	male	green	good
2565	2012	Sjollen	S5	60,30	male	green	good
2566	2012	Sjollen	S5	62,90	male	green	good
2567	2012	Sjollen	S5	66,40	male	green	good
2568	2012	Sjollen	S5	67,00	male	green	good
2569	2012	Sjollen	S5	67,10	male	green	good
2570	2012	Sjollen	S5	67,80	male	green	reduced
2571	2012	Sjollen	S5	54,80	male	red	good
2572	2012	Sjollen	S5	55,00	male	red	good
2573	2012	Sjollen	S5	55,40	male	red	good
2574	2012	Sjollen	S5	55,60	male	red	good
2575	2012	Sjollen	S5	57,60	male	red	reduced
2576	2012	Sjollen	S5	58,70	male	red	good
2577	2012	Sjollen	S5	59,30	male	red	good
2578	2012	Sjollen	S5	59,90	male	red	reduced
2579	2012	Sjollen	S5	59,90	male	red	good
2580	2012	Sjollen	S5	60,80	male	red	reduced
2581	2012	Sjollen	S5	62,60	male	red	good
2582	2012	Sjollen	S5	64,40	male	red	reduced
2583	2012	Sjollen	S5	53,40	male	green	reduced
2584	2012	Sjollen	S5	54,60	male	green	good
2585	2012	Sjollen	S5	54,90	male	green	good
2586	2012	Sjollen	S5	56,30	male	green	good
2587	2012	Sjollen	S5	57,10	male	green	good
2588	2012	Sjollen	S5	57,40	male	green	good
2589	2012	Sjollen	S5	59,10	male	green	reduced
2590	2012	Sjollen	S5	59,80	male	green	good
2591	2012	Sjollen	S5	60,00	male	green	good
2592	2012	Sjollen	S5	61,20	male	green	reduced

2593	2012	Sjollen	S5	62,70	male	green	good
2594	2012	Sjollen	S5	63,40	male	green	good
2595	2012	Sjollen	S5	66,50	male	green	good
2596	2012	Sjollen	S5	67,10	male	green	reduced
2597	2012	Sjollen	S5	67,70	male	green	good
2598	2012	Sjollen	S5	44,10	female	red	reduced
2599	2012	Sjollen	S5	47,10	female	red	good
2600	2012	Sjollen	S5	47,60	female	red	good
2601	2012	Sjollen	S5	50,30	female	red	good
2602	2012	Sjollen	S5	53,60	male	red	reduced
2603	2012	Sjollen	S5	55,80	male	red	good
2604	2012	Sjollen	S5	57,00	male	red	good
2605	2012	Sjollen	S5	57,60	male	red	good
2606	2012	Sjollen	S5	58,80	male	red	good
2607	2012	Sjollen	S5	59,40	male	red	good
2608	2012	Sjollen	S5	59,80	male	red	good
2609	2012	Sjollen	S5	60,40	male	red	good
2610	2012	Sjollen	S5	60,50	male	red	good
2611	2012	Sjollen	S5	61,00	male	red	good
2612	2012	Sjollen	S5	61,70	male	red	good
2613	2012	Sjollen	S5	62,00	male	red	good
2614	2012	Sjollen	S5	62,50	male	red	good
2615	2012	Sjollen	S5	64,00	male	red	good
2616	2012	Sjollen	S5	65,50	male	red	good
2617	2012	Sjollen	S5	65,80	male	red	good
2618	2012	Sjollen	S5	67,10	male	red	good
2619	2012	Sjollen	S6	46,90	male	green	good
2620	2012	Sjollen	S6	50,70	male	green	good
2621	2012	Sjollen	S6	52,00	male	green	good
2622	2012	Sjollen	S6	57,60	male	green	good
2623	2012	Sjollen	S6	59,10	male	green	good
2624	2012	Sjollen	S6	59,40	male	green	good
2625	2012	Sjollen	S6	63,50	male	green	good
2626	2012	Sjollen	S6	65,70	male	green	good
2627	2012	Sjollen	S6	48,70	male	red	good
2628	2012	Sjollen	S6	49,70	male	red	good
2629	2012	Sjollen	S6	50,20	male	red	good
2630	2012	Sjollen	S6	51,50	male	red	good
2631	2012	Sjollen	S6	51,90	male	red	good
2632	2012	Sjollen	S6	52,60	male	red	good
2633	2012	Sjollen	S6	53,60	male	red	good
2634	2012	Sjollen	S6	53,80	male	red	good
2635	2012	Sjollen	S6	54,40	male	red	good
2636	2012	Sjollen	S6	55,20	male	red	good
2637	2012	Sjollen	S6	55,70	male	red	good
2638	2012	Sjollen	S6	56,10	male	red	reduced
2639	2012	Sjollen	S6	56,70	male	red	good
2640	2012	Sjollen	S6	57,30	male	red	good
2641	2012	Sjollen	S6	58,00	male	red	good
2642	2012	Sjollen	S6	58,10	male	red	good
2643	2012	Sjollen	S6	59,60	male	red	good
2644	2012	Sjollen	S6	60,00	male	red	reduced
2645	2012	Sjollen	S6	60,60	male	red	good
2646	2012	Sjollen	S6	61,40	male	green	good
2647	2012	Sjollen	S6	45,30	female	red	good
2648	2012	Sjollen	S6	47,00	male	red	good
2649	2012	Sjollen	S6	55,30	male	red	good
2650	2012	Sjollen	S6	57,30	male	red	reduced
2651	2012	Sjollen	S6	58,00	male	red	good

2652	2012	Sjollen	S6	59,30	male	red	good
2653	2012	Sjollen	S6	55,90	male	green	good
2654	2012	Sjollen	S6	58,90	male	green	reduced
2655	2012	Sjollen	S6	59,00	male	green	reduced
2656	2012	Sjollen	S6	64,30	male	green	good
2657	2012	Sjollen	S6	64,40	male	green	reduced
2658	2012	Sjollen	S6	69,80	male	green	reduced
2659	2012	Sjollen	S6	54,60	male	red	good
2660	2012	Sjollen	S6	58,90	male	red	reduced
2661	2012	Sjollen	S6	61,20	male	red	good
2662	2012	Sjollen	S6	68,00	male	red	good
2663	2012	Sjollen	S6	69,20	male	red	good
2664	2012	Sjollen	S6	50,80	male	green	good
2665	2012	Sjollen	S6	54,90	male	red	reduced
2666	2012	Sjollen	S6	58,30	male	red	reduced
2667	2012	Sjollen	S6	55,00	male	green	good
2668	2012	Sjollen	S6	57,40	male	green	good
2669	2012	Sjollen	S6	57,50	male	green	reduced
2670	2012	Sjollen	S6	58,40	male	green	good
2671	2012	Sjollen	S6	58,80	male	green	good
2672	2012	Sjollen	S6	58,80	male	green	good
2673	2012	Sjollen	S6	58,90	male	green	good
2674	2012	Sjollen	S6	61,10	male	green	good
2675	2012	Sjollen	S6	62,20	male	green	good
2676	2012	Sjollen	S6	62,20	male	green	good
2677	2012	Sjollen	S6	62,70	male	green	good
2678	2012	Sjollen	S6	63,80	male	green	good
2679	2012	Sjollen	S6	64,10	male	green	good
2680	2012	Sjollen	S6	73,50	male	green	reduced
2681	2012	Sjollen	S6	52,50	male	red	good
2682	2012	Sjollen	S6	54,00	male	red	good
2683	2012	Sjollen	S6	59,20	male	red	good
2684	2012	Sjollen	S6	62,50	male	red	good
2685	2012	Sjollen	S6	65,00	male	red	good
2686	2012	Sjollen	S6	52,80	male	green	good
2687	2012	Sjollen	S6	55,10	male	green	good
2688	2012	Sjollen	S6	56,00	male	green	reduced
2689	2012	Sjollen	S6	59,00	male	green	good
2690	2012	Sjollen	S6	64,50	male	green	good
2691	2012	Sjollen	S6	65,10	male	green	good
2692	2012	Sjollen	S6	42,60	female	red	good
2693	2012	Sjollen	S6	51,70	female	red	good
2694	2012	Sjollen	S6	54,80	male	red	reduced
2695	2012	Sjollen	S6	58,80	male	red	good
2696	2012	Sjollen	S6	60,20	male	red	good
2697	2012	Sjollen	S6	64,40	male	red	good
2698	2012	Sjollen	S6	67,60	male	red	good
2699	2012	Sjollen	S7	54,90	male	red	reduced
2700	2012	Sjollen	S7	59,10	male	red	good
2701	2012	Sjollen	S7	62,50	male	red	good
2702	2012	Sjollen	S7	63,40	male	green	good
2703	2012	Sjollen	S7	51,10	male	red	good
2704	2012	Sjollen	S7	57,80	male	red	good
2705	2012	Sjollen	S7	48,60	male	red	good
2706	2012	Sjollen	S7	55,70	male	red	good
2707	2012	Sjollen	S7	60,20	male	red	good
2708	2012	Sjollen	S7	64,70	male	red	reduced
2709	2012	Sjollen	S7	64,70	male	red	good
2710	2012	Sjollen	S7	55,40	male	red	reduced

2711	2012	Sjollen	S7	55,40	male	red	good
2712	2012	Sjollen	S7	42,50	male	green	good
2713	2012	Sjollen	S7	55,90	male	green	good
2714	2012	Sjollen	S7	65,70	male	green	good
2715	2012	Sjollen	S7	70,90	male	green	reduced
2716	2012	Sjollen	S7	45,00	female	red	reduced
2717	2012	Sjollen	S7	61,50	male	red	good
2718	2012	Sjollen	S7	65,50	male	red	good
2719	2012	Sjollen	S7	57,50	male	green	good
2720	2012	Sjollen	S7	61,40	male	green	good
2721	2012	Sjollen	S8	55,60	male	red	reduced
2722	2012	Sjollen	S8	59,10	male	red	reduced
2723	2012	Sjollen	S8	47,80	male	green	good
2724	2012	Sjollen	S8	57,30	male	green	reduced
2725	2012	Sjollen	S8	50,00	male	red	good
2726	2012	Sjollen	S8	50,40	male	red	good
2727	2012	Sjollen	S8	51,00	male	red	good
2728	2012	Sjollen	S8	55,30	male	red	reduced
2729	2012	Sjollen	S8	56,50	male	red	good
2730	2012	Sjollen	S8	34,10	male	green	good
2731	2012	Sjollen	S8	55,00	male	green	good
2732	2012	Sjollen	S8	55,70	male	green	good
2733	2012	Sjollen	S8	63,20	male	green	good
2734	2012	Sjollen	S8	46,00	male	red	good
2735	2012	Sjollen	S8	55,00	male	red	good
2736	2012	Sjollen	S8	55,90	male	red	reduced
2737	2012	Sjollen	S8	59,60	male	red	reduced
2738	2012	Sjollen	S8	61,40	male	red	good
2739	2012	Sjollen	S8	51,50	male	green	good
2740	2012	Sjollen	S8	63,80	male	red	good
2741	2012	Sjollen	S8	50,30	male	green	reduced
2742	2012	Sjollen	S8	50,80	male	green	reduced
2743	2012	Sjollen	S8	57,80	male	green	good
2744	2012	Sjollen	S8	58,90	male	green	good
2745	2012	Sjollen	S8	59,10	male	green	good
2746	2012	Sjollen	S8	60,50	male	green	good
2747	2012	Sjollen	S8	61,30	male	green	good
2748	2012	Sjollen	S8	61,60	male	green	good
2749	2012	Sjollen	S8	62,80	male	green	good
2750	2012	Sjollen	S8	62,90	male	green	good
2751	2012	Sjollen	S8	63,00	male	green	good
2752	2012	Sjollen	S8	65,60	male	green	reduced
2753	2012	Sjollen	S8	66,90	male	green	good
2754	2012	Sjollen	S8	64,90	male	red	good
2755	2012	Sjollen	S8	40,70	male	green	good
2756	2012	Sjollen	S8	47,30	male	green	reduced
2757	2012	Sjollen	S8	49,50	male	green	good
2758	2012	Sjollen	S8	55,60	male	green	good
2759	2012	Sjollen	S8	56,10	male	green	good
2760	2012	Sjollen	S8	58,10	male	green	good
2761	2012	Sjollen	S8	58,20	male	green	good
2762	2012	Sjollen	S8	58,60	male	green	good
2763	2012	Sjollen	S8	60,00	male	green	reduced
2764	2012	Sjollen	S8	60,00	male	green	good
2765	2012	Sjollen	S8	60,10	male	green	good
2766	2012	Sjollen	S8	67,70	male	green	good
2767	2012	Sjollen	S8	68,70	male	green	good
2768	2012	Sjollen	S8	49,70	male	red	good
2769	2012	Sjollen	S8	53,10	male	red	good

2770	2012	Sjollen	S8	53,80	male	red	good
2771	2012	Sjollen	S8	55,70	male	red	good
2772	2012	Sjollen	S8	55,90	male	red	good
2773	2012	Sjollen	S8	58,00	male	red	good
2774	2012	Sjollen	S8	58,50	male	red	good
2775	2012	Sjollen	S8	68,80	male	red	good
2776	2012	Sjollen	S9	57,10	male	red	good
2777	2012	Sjollen	S9	58,90	male	red	good
2778	2012	Sjollen	S9	60,40	male	red	good
2779	2012	Sjollen	S9	62,40	male	red	good
2780	2012	Sjollen	S9	49,60	male	red	good
2781	2012	Sjollen	S9	61,80	male	red	reduced
2782	2012	Sjollen	S9	40,20	male	green	good
2783	2012	Sjollen	S9	47,50	male	green	good
2784	2012	Sjollen	S9	44,10	male	red	good
2785	2012	Sjollen	S9	44,40	female	red	good
2786	2012	Sjollen	S9	51,60	male	red	good
2787	2012	Sjollen	S9	52,30	male	red	reduced
2788	2012	Sjollen	S9	57,40	male	red	good
2789	2012	Sjollen	S9	57,50	male	red	good
2790	2012	Sjollen	S9	62,30	male	red	good
2791	2012	Sjollen	S9	62,10	male	red	good
2792	2012	Sjollen	S9	43,40	female	green	good
2793	2012	Sjollen	S9	43,70	male	green	good
2794	2012	Sjollen	S9	45,80	male	green	good
2795	2012	Sjollen	S9	57,40	male	green	reduced
2796	2012	Sjollen	S9	42,50	female	red	good
2797	2012	Sjollen	S9	58,90	male	red	reduced
2798	2012	Sjollen	S9	42,30	male	green	good
2799	2012	Sjollen	S9	50,60	male	green	good
2800	2012	Sjollen	S9	52,10	male	green	good
2801	2012	Sjollen	S9	54,10	male	red	good
2802	2012	Sjollen	S9	56,30	male	red	good
2803	2012	Sjollen	S9	57,80	male	red	good
2804	2012	Sjollen	S9	66,30	male	red	reduced
2805	2012	Lillgrund	L1	61,50	male	green	good
2806	2012	Lillgrund	L1	60,10	male	red	good
2807	2012	Lillgrund	L1	61,50	male	red	good
2808	2012	Lillgrund	L1	60,10	male	red	reduced
2809	2012	Lillgrund	L1	46,00	female	red	good
2810	2012	Lillgrund	L1	56,40	male	red	good
2811	2012	Lillgrund	L1	58,20	male	green	good
2812	2012	Lillgrund	L1	60,10	male	green	good
2813	2012	Lillgrund	L1	64,90	male	green	good
2814	2012	Lillgrund	L1	66,50	male	green	good
2815	2012	Lillgrund	L1	68,70	male	green	good
2816	2012	Lillgrund	L1	68,70	male	green	good
2817	2012	Lillgrund	L1	70,30	male	green	good
2818	2012	Lillgrund	L1	54,40	male	red	reduced
2819	2012	Lillgrund	L1	47,20	female	red	good
2820	2012	Lillgrund	L1	54,20	male	red	good
2821	2012	Lillgrund	L1	57,40	male	red	good
2822	2012	Lillgrund	L1	57,50	female	red	good
2823	2012	Lillgrund	L1	62,50	male	red	good
2824	2012	Lillgrund	L1	63,80	male	red	good
2825	2012	Lillgrund	L1	64,30	male	red	good
2826	2012	Lillgrund	L1	67,00	male	red	good
2827	2012	Lillgrund	L1	59,40	male	green	reduced
2828	2012	Lillgrund	L1	63,50	male	green	reduced

2829	2012	Lillgrund	L1	54,70	male	green	good
2830	2012	Lillgrund	L1	67,60	male	green	good
2831	2012	Lillgrund	L1	67,80	male	green	good
2832	2012	Lillgrund	L1	69,20	male	green	good
2833	2012	Lillgrund	L1	71,80	male	green	good
2834	2012	Lillgrund	L1	71,80	male	green	good
2835	2012	Lillgrund	L1	59,20	male	red	reduced
2836	2012	Lillgrund	L1	59,70	male	red	reduced
2837	2012	Lillgrund	L1	62,20	male	red	reduced
2838	2012	Lillgrund	L1	66,10	male	red	reduced
2839	2012	Lillgrund	L1	41,60	female	red	good
2840	2012	Lillgrund	L1	45,70	female	red	good
2841	2012	Lillgrund	L1	46,40	female	red	good
2842	2012	Lillgrund	L1	47,20	female	red	good
2843	2012	Lillgrund	L1	48,20	female	red	good
2844	2012	Lillgrund	L1	58,00	male	red	good
2845	2012	Lillgrund	L1	58,50	male	red	good
2846	2012	Lillgrund	L1	58,60	male	red	good
2847	2012	Lillgrund	L1	61,40	male	red	good
2848	2012	Lillgrund	L1	61,90	male	red	good
2849	2012	Lillgrund	L1	62,40	male	red	good
2850	2012	Lillgrund	L1	65,80	male	red	good
2851	2012	Lillgrund	L1	67,00	male	red	good
2852	2012	Lillgrund	L1	64,80	male	green	reduced
2853	2012	Lillgrund	L1	58,70	male	green	good
2854	2012	Lillgrund	L1	59,10	male	green	good
2855	2012	Lillgrund	L1	62,60	male	green	good
2856	2012	Lillgrund	L1	63,40	male	green	good
2857	2012	Lillgrund	L1	64,60	male	green	good
2858	2012	Lillgrund	L1	64,90	male	green	good
2859	2012	Lillgrund	L1	65,50	male	green	good
2860	2012	Lillgrund	L1	54,30	female	red	good
2861	2012	Lillgrund	L1	57,50	male	red	good
2862	2012	Lillgrund	L1	59,90	male	red	good
2863	2012	Lillgrund	L1	60,10	male	red	good
2864	2012	Lillgrund	L1	61,80	male	red	good
2865	2012	Lillgrund	L1	56,40	male	green	reduced
2866	2012	Lillgrund	L1	63,30	male	green	reduced
2867	2012	Lillgrund	L1	67,10	male	green	reduced
2868	2012	Lillgrund	L1	57,50	male	green	good
2869	2012	Lillgrund	L1	59,20	male	green	good
2870	2012	Lillgrund	L1	60,60	male	green	good
2871	2012	Lillgrund	L1	61,80	male	green	good
2872	2012	Lillgrund	L1	62,40	male	green	good
2873	2012	Lillgrund	L1	63,20	male	green	good
2874	2012	Lillgrund	L1	63,80	male	green	good
2875	2012	Lillgrund	L1	64,10	male	green	good
2876	2012	Lillgrund	L1	64,50	male	green	good
2877	2012	Lillgrund	L1	64,60	male	green	good
2878	2012	Lillgrund	L1	65,60	male	green	good
2879	2012	Lillgrund	L1	65,80	male	green	good
2880	2012	Lillgrund	L1	65,90	male	green	good
2881	2012	Lillgrund	L1	66,20	male	green	good
2882	2012	Lillgrund	L1	66,30	male	green	good
2883	2012	Lillgrund	L1	68,50	male	green	good
2884	2012	Lillgrund	L1	70,60	male	green	good
2885	2012	Lillgrund	L1	70,70	male	green	good
2886	2012	Lillgrund	L1	73,00	male	green	good
2887	2012	Lillgrund	L1	62,80	male	red	reduced

2888	2012	Lillgrund	L1	45,60	female	red	good
2889	2012	Lillgrund	L1	46,50	female	red	good
2890	2012	Lillgrund	L1	47,70	male	red	good
2891	2012	Lillgrund	L1	56,00	male	red	good
2892	2012	Lillgrund	L1	56,10	male	red	good
2893	2012	Lillgrund	L1	56,80	male	red	good
2894	2012	Lillgrund	L1	58,00	male	red	good
2895	2012	Lillgrund	L1	58,70	male	red	good
2896	2012	Lillgrund	L1	59,70	male	red	good
2897	2012	Lillgrund	L1	60,30	male	red	good
2898	2012	Lillgrund	L1	61,00	male	red	good
2899	2012	Lillgrund	L1	62,70	male	red	good
2900	2012	Lillgrund	L1	62,90	male	red	good
2901	2012	Lillgrund	L1	63,40	male	red	good
2902	2012	Lillgrund	L1	63,40	male	red	good
2903	2012	Lillgrund	L1	64,40	male	red	good
2904	2012	Lillgrund	L1	69,00	male	red	good
2905	2012	Lillgrund	L1	69,80	male	red	good
2906	2012	Lillgrund	L10	58,90	male	red	reduced
2907	2012	Lillgrund	L10	61,70	male	red	reduced
2908	2012	Lillgrund	L10	47,50	male	red	good
2909	2012	Lillgrund	L10	48,60	male	red	good
2910	2012	Lillgrund	L10	50,00	male	red	good
2911	2012	Lillgrund	L10	54,30	male	red	good
2912	2012	Lillgrund	L10	55,00	male	red	good
2913	2012	Lillgrund	L10	56,30	male	red	good
2914	2012	Lillgrund	L10	56,30	male	red	good
2915	2012	Lillgrund	L10	57,50	male	red	good
2916	2012	Lillgrund	L10	58,20	male	red	good
2917	2012	Lillgrund	L10	58,40	male	red	good
2918	2012	Lillgrund	L10	59,30	male	red	good
2919	2012	Lillgrund	L10	59,80	male	red	good
2920	2012	Lillgrund	L10	59,90	male	red	good
2921	2012	Lillgrund	L10	60,50	male	red	good
2922	2012	Lillgrund	L10	60,90	male	red	good
2923	2012	Lillgrund	L10	61,00	male	red	good
2924	2012	Lillgrund	L10	61,70	male	red	good
2925	2012	Lillgrund	L10	62,00	male	red	good
2926	2012	Lillgrund	L10	62,30	male	red	good
2927	2012	Lillgrund	L10	65,40	male	red	good
2928	2012	Lillgrund	L10	66,60	male	red	good
2929	2012	Lillgrund	L10	67,30	male	red	good
2930	2012	Lillgrund	L10	52,20	male	green	good
2931	2012	Lillgrund	L10	58,20	male	green	good
2932	2012	Lillgrund	L10	57,50	male	red	reduced
2933	2012	Lillgrund	L10	44,40	male	red	good
2934	2012	Lillgrund	L10	47,90	male	red	good
2935	2012	Lillgrund	L10	52,80	male	red	good
2936	2012	Lillgrund	L10	54,00	male	red	good
2937	2012	Lillgrund	L10	54,10	male	red	good
2938	2012	Lillgrund	L10	57,90	male	red	good
2939	2012	Lillgrund	L10	58,90	male	red	good
2940	2012	Lillgrund	L10	67,20	male	green	reduced
2941	2012	Lillgrund	L10	58,50	male	green	good
2942	2012	Lillgrund	L10	59,70	male	green	good
2943	2012	Lillgrund	L10	59,80	male	green	good
2944	2012	Lillgrund	L10	61,30	male	green	good
2945	2012	Lillgrund	L10	62,40	male	green	good
2946	2012	Lillgrund	L10	48,30	female	red	good

2947	2012	Lillgrund	L10	51,70	female	red	good
2948	2012	Lillgrund	L10	52,10	male	red	good
2949	2012	Lillgrund	L10	53,10	male	red	good
2950	2012	Lillgrund	L10	57,80	male	red	good
2951	2012	Lillgrund	L10	59,00	male	red	good
2952	2012	Lillgrund	L10	59,40	male	red	good
2953	2012	Lillgrund	L10	61,40	male	red	good
2954	2012	Lillgrund	L10	61,70	male	red	good
2955	2012	Lillgrund	L10	64,10	male	red	good
2956	2012	Lillgrund	L10	64,20	male	red	good
2957	2012	Lillgrund	L10	56,00	male	green	good
2958	2012	Lillgrund	L10	56,60	male	green	good
2959	2012	Lillgrund	L10	61,90	male	green	good
2960	2012	Lillgrund	L10	67,20	male	green	good
2961	2012	Lillgrund	L10	62,50	male	red	reduced
2962	2012	Lillgrund	L10	63,00	male	red	reduced
2963	2012	Lillgrund	L10	39,90	male	red	good
2964	2012	Lillgrund	L10	43,10	female	red	good
2965	2012	Lillgrund	L10	45,80	female	red	good
2966	2012	Lillgrund	L10	50,40	female	red	good
2967	2012	Lillgrund	L10	57,30	male	red	good
2968	2012	Lillgrund	L10	58,50	male	red	good
2969	2012	Lillgrund	L10	60,00	male	red	good
2970	2012	Lillgrund	L10	60,10	male	red	good
2971	2012	Lillgrund	L10	60,40	male	red	good
2972	2012	Lillgrund	L10	61,00	male	red	good
2973	2012	Lillgrund	L10	62,50	male	red	good
2974	2012	Lillgrund	L10	63,40	male	red	good
2975	2012	Lillgrund	L10	67,00	male	red	good
2976	2012	Lillgrund	L10	67,50	male	red	good
2977	2012	Lillgrund	l10	65,30	male	green	reduced
2978	2012	Lillgrund	l10	58,60	male	green	good
2979	2012	Lillgrund	l10	59,00	male	green	good
2980	2012	Lillgrund	L10	62,40	male	green	good
2981	2012	Lillgrund	l10	63,90	male	green	good
2982	2012	Lillgrund	l10	66,20	male	green	good
2983	2012	Lillgrund	l10	69,00	male	green	good
2984	2012	Lillgrund	l10	69,20	male	green	good
2985	2012	Lillgrund	l10	69,40	male	green	good
2986	2012	Lillgrund	l10	59,70	male	red	reduced
2987	2012	Lillgrund	l10	60,10	male	red	reduced
2988	2012	Lillgrund	l10	63,90	male	red	reduced
2989	2012	Lillgrund	l10	60,20	male	red	good
2990	2012	Lillgrund	l10	64,00	male	red	good
2991	2012	Lillgrund	L10	59,90	male	green	reduced
2992	2012	Lillgrund	L10	55,70	male	green	good
2993	2012	Lillgrund	L10	60,20	male	green	good
2994	2012	Lillgrund	L10	63,60	male	green	good
2995	2012	Lillgrund	L10	64,70	male	green	good
2996	2012	Lillgrund	L10	65,20	male	green	good
2997	2012	Lillgrund	L10	55,30	male	red	reduced
2998	2012	Lillgrund	L10	60,10	male	red	reduced
2999	2012	Lillgrund	L10	63,50	male	red	reduced
3000	2012	Lillgrund	L10	49,20	female	red	good
3001	2012	Lillgrund	L10	53,80	male	red	good
3002	2012	Lillgrund	L10	56,80	male	red	good
3003	2012	Lillgrund	L10	57,60	male	red	good
3004	2012	Lillgrund	L10	58,20	male	red	good
3005	2012	Lillgrund	L10	59,00	male	red	good

3006	2012	Lillgrund	L10	59,20	male	red	good
3007	2012	Lillgrund	L10	59,90	male	red	good
3008	2012	Lillgrund	L10	60,80	male	red	good
3009	2012	Lillgrund	L10	60,80	male	red	good
3010	2012	Lillgrund	L10	61,60	male	red	good
3011	2012	Lillgrund	L10	62,40	male	red	good
3012	2012	Lillgrund	L10	65,50	male	red	good
3013	2012	Lillgrund	L10	66,40	male	red	good
3014	2012	Lillgrund	L10	68,00	male	red	good
3015	2012	Lillgrund	L2	61,10	male	red	reduced
3016	2012	Lillgrund	L2	63,40	male	red	reduced
3017	2012	Lillgrund	L2	67,50	male	red	reduced
3018	2012	Lillgrund	L2	56,50	male	red	good
3019	2012	Lillgrund	L2	57,90	male	red	good
3020	2012	Lillgrund	L2	56,60	male	green	good
3021	2012	Lillgrund	L2	45,40	female	red	reduced
3022	2012	Lillgrund	L2	59,10	male	red	reduced
3023	2012	Lillgrund	L2	43,00	female	red	good
3024	2012	Lillgrund	L2	50,80	male	red	good
3025	2012	Lillgrund	L2	56,00	male	red	good
3026	2012	Lillgrund	L2	58,90	male	red	good
3027	2012	Lillgrund	L2	60,70	male	red	good
3028	2012	Lillgrund	L2	60,90	male	red	good
3029	2012	Lillgrund	L2	61,40	male	red	good
3030	2012	Lillgrund	L2	62,00	male	red	good
3031	2012	Lillgrund	L2	63,00	male	red	good
3032	2012	Lillgrund	L2	67,50	male	red	good
3033	2012	Lillgrund	L2	58,20	male	green	reduced
3034	2012	Lillgrund	L2	73,10	male	green	reduced
3035	2012	Lillgrund	L2	49,60	male	green	good
3036	2012	Lillgrund	L2	60,10	male	green	good
3037	2012	Lillgrund	L2	60,80	male	green	good
3038	2012	Lillgrund	L2	65,60	male	green	good
3039	2012	Lillgrund	L2	67,20	male	green	good
3040	2012	Lillgrund	L2	67,30	male	green	good
3041	2012	Lillgrund	L2	67,60	male	green	good
3042	2012	Lillgrund	L2	68,50	male	green	good
3043	2012	Lillgrund	L2	52,50	male	red	reduced
3044	2012	Lillgrund	L2	58,90	male	red	reduced
3045	2012	Lillgrund	L2	43,20	female	red	good
3046	2012	Lillgrund	L2	44,20	female	red	good
3047	2012	Lillgrund	L2	45,60	female	red	good
3048	2012	Lillgrund	L2	47,90	female	red	good
3049	2012	Lillgrund	L2	62,00	male	red	good
3050	2012	Lillgrund	L2	63,40	male	red	good
3051	2012	Lillgrund	L2	63,60	male	green	reduced
3052	2012	Lillgrund	L2	49,40	female	green	good
3053	2012	Lillgrund	L2	56,80	male	green	good
3054	2012	Lillgrund	L2	59,80	male	green	good
3055	2012	Lillgrund	L2	65,40	male	green	good
3056	2012	Lillgrund	L2	66,70	male	green	good
3057	2012	Lillgrund	L2	66,90	male	green	good
3058	2012	Lillgrund	L2	69,10	male	green	good
3059	2012	Lillgrund	L2	54,90	male	red	reduced
3060	2012	Lillgrund	L2	40,30	female	red	good
3061	2012	Lillgrund	L2	47,30	female	red	good
3062	2012	Lillgrund	L2	48,50	female	red	good
3063	2012	Lillgrund	L2	51,30	male	red	good
3064	2012	Lillgrund	L2	59,70	male	red	good

3065	2012	Lillgrund	L2	61,90	male	red	good
3066	2012	Lillgrund	L2	62,50	male	red	good
3067	2012	Lillgrund	L2	61,30	male	green	reduced
3068	2012	Lillgrund	L2	66,70	male	green	reduced
3069	2012	Lillgrund	L2	68,20	male	green	reduced
3070	2012	Lillgrund	L2	54,40	male	green	good
3071	2012	Lillgrund	L2	59,00	male	green	good
3072	2012	Lillgrund	L2	61,30	male	green	good
3073	2012	Lillgrund	L2	62,70	male	green	good
3074	2012	Lillgrund	L2	62,80	male	green	good
3075	2012	Lillgrund	L2	63,30	male	green	good
3076	2012	Lillgrund	L2	67,30	male	green	good
3077	2012	Lillgrund	L2	69,30	male	green	good
3078	2012	Lillgrund	L2	69,80	male	green	good
3079	2012	Lillgrund	L2	70,00	male	green	good
3080	2012	Lillgrund	L2	70,00	male	green	good
3081	2012	Lillgrund	L2	70,60	male	green	good
3082	2012	Lillgrund	L2	74,20	male	green	good
3083	2012	Lillgrund	L2	57,40	male	red	good
3084	2012	Lillgrund	L2	60,00	male	red	good
3085	2012	Lillgrund	L2	62,80	male	red	good
3086	2012	Lillgrund	L2	63,50	male	red	good
3087	2012	Lillgrund	L2	63,70	male	red	good
3088	2012	Lillgrund	L2	64,20	male	red	good
3089	2012	Lillgrund	L2	65,80	male	red	good
3090	2012	Lillgrund	L2	67,00	male	red	good
3091	2012	Lillgrund	L2	59,20	male	green	reduced
3092	2012	Lillgrund	L2	61,50	male	green	reduced
3093	2012	Lillgrund	L2	63,80	male	green	reduced
3094	2012	Lillgrund	L2	55,90	male	green	good
3095	2012	Lillgrund	L2	56,30	male	green	good
3096	2012	Lillgrund	L2	58,00	male	green	good
3097	2012	Lillgrund	L2	58,00	male	green	good
3098	2012	Lillgrund	L2	58,30	male	green	good
3099	2012	Lillgrund	L2	58,70	male	green	good
3100	2012	Lillgrund	L2	62,40	male	green	good
3101	2012	Lillgrund	L2	65,40	male	green	good
3102	2012	Lillgrund	L2	66,10	male	green	good
3103	2012	Lillgrund	L2	66,30	male	green	good
3104	2012	Lillgrund	L2	67,50	male	green	good
3105	2012	Lillgrund	L2	68,20	male	green	good
3106	2012	Lillgrund	L2	68,20	male	green	good
3107	2012	Lillgrund	L2	68,20	male	green	good
3108	2012	Lillgrund	L2	68,40	male	green	good
3109	2012	Lillgrund	L2	69,50	male	green	good
3110	2012	Lillgrund	L2	70,40	male	green	good
3111	2012	Lillgrund	L2	70,50	male	green	good
3112	2012	Lillgrund	L2	72,50	male	green	good
3113	2012	Lillgrund	L2	54,40	male	red	reduced
3114	2012	Lillgrund	L2	54,50	male	red	reduced
3115	2012	Lillgrund	L2	63,10	male	red	reduced
3116	2012	Lillgrund	L2	63,20	male	red	reduced
3117	2012	Lillgrund	L2	65,70	male	red	reduced
3118	2012	Lillgrund	L2	53,10	male	red	good
3119	2012	Lillgrund	L2	54,40	male	red	good
3120	2012	Lillgrund	L2	56,60	male	red	good
3121	2012	Lillgrund	L2	56,70	male	red	good
3122	2012	Lillgrund	L2	57,00	male	red	good
3123	2012	Lillgrund	L2	57,30	male	red	good

3124	2012	Lillgrund	L2	57,40	male	red	good
3125	2012	Lillgrund	L2	58,30	male	red	good
3126	2012	Lillgrund	L2	60,30	male	red	good
3127	2012	Lillgrund	L2	60,50	male	red	good
3128	2012	Lillgrund	L2	61,80	male	red	good
3129	2012	Lillgrund	L2	62,30	male	red	good
3130	2012	Lillgrund	L2	62,40	male	red	good
3131	2012	Lillgrund	L2	65,50	male	red	good
3132	2012	Lillgrund	L2	66,30	male	red	good
3133	2012	Lillgrund	L2	69,10	male	red	good
3134	2012	Lillgrund	L3	60,90	male	green	reduced
3135	2012	Lillgrund	L3	55,70	male	red	reduced
3136	2012	Lillgrund	L3	59,20	male	red	reduced
3137	2012	Lillgrund	L3	57,00	male	red	good
3138	2012	Lillgrund	L3	57,10	male	green	good
3139	2012	Lillgrund	L3	46,20	female	red	reduced
3140	2012	Lillgrund	L3	51,10	male	red	reduced
3141	2012	Lillgrund	L3	60,40	male	red	good
3142	2012	Lillgrund	L3	67,70	male	red	good
3143	2012	Lillgrund	L3	66,10	male	green	reduced
3144	2012	Lillgrund	L3	57,90	male	green	good
3145	2012	Lillgrund	L3	68,60	male	green	good
3146	2012	Lillgrund	L3	56,80	male	red	reduced
3147	2012	Lillgrund	L3	59,00	male	red	reduced
3148	2012	Lillgrund	L3	59,30	male	red	reduced
3149	2012	Lillgrund	L3	45,70	female	red	good
3150	2012	Lillgrund	L3	49,50	male	red	good
3151	2012	Lillgrund	L3	50,70	female	red	good
3152	2012	Lillgrund	L3	58,10	male	red	good
3153	2012	Lillgrund	L3	58,80	male	red	good
3154	2012	Lillgrund	L3	59,20	male	red	good
3155	2012	Lillgrund	L3	60,60	male	red	good
3156	2012	Lillgrund	L3	64,30	male	red	good
3157	2012	Lillgrund	L3	64,30	male	red	good
3158	2012	Lillgrund	L3	65,90	male	red	good
3159	2012	Lillgrund	L3	67,40	male	red	good
3160	2012	Lillgrund	L3	60,30	male	green	good
3161	2012	Lillgrund	L3	64,80	male	green	good
3162	2012	Lillgrund	L3	67,20	male	green	good
3163	2012	Lillgrund	L3	68,70	male	green	good
3164	2012	Lillgrund	L3	66,10	male	red	reduced
3165	2012	Lillgrund	L3	41,40	female	red	good
3166	2012	Lillgrund	L3	50,00	female	red	good
3167	2012	Lillgrund	L3	52,40	female	red	good
3168	2012	Lillgrund	L3	55,90	male	red	good
3169	2012	Lillgrund	L3	57,80	male	red	good
3170	2012	Lillgrund	L3	59,00	male	red	good
3171	2012	Lillgrund	L3	59,20	male	red	good
3172	2012	Lillgrund	L3	59,40	male	red	good
3173	2012	Lillgrund	L3	60,60	male	red	good
3174	2012	Lillgrund	L3	63,50	male	red	good
3175	2012	Lillgrund	L3	63,60	male	red	good
3176	2012	Lillgrund	L3	58,80	male	green	good
3177	2012	Lillgrund	L3	64,40	male	green	good
3178	2012	Lillgrund	L3	66,60	male	green	good
3179	2012	Lillgrund	L3	68,00	male	green	good
3180	2012	Lillgrund	L3	70,90	male	green	good
3181	2012	Lillgrund	L3	57,90	male	red	reduced
3182	2012	Lillgrund	L3	58,30	male	red	reduced

3183	2012	Lillgrund	L3	48,20	male	red	good
3184	2012	Lillgrund	L3	62,70	male	green	reduced
3185	2012	Lillgrund	L3	64,70	male	green	reduced
3186	2012	Lillgrund	L3	67,80	male	green	reduced
3187	2012	Lillgrund	L3	54,20	male	green	good
3188	2012	Lillgrund	L3	56,00	male	green	good
3189	2012	Lillgrund	L3	56,80	male	green	good
3190	2012	Lillgrund	L3	59,20	male	green	good
3191	2012	Lillgrund	L3	60,50	male	green	good
3192	2012	Lillgrund	L3	60,60	male	green	good
3193	2012	Lillgrund	L3	62,00	male	green	good
3194	2012	Lillgrund	L3	66,90	male	green	good
3195	2012	Lillgrund	L3	70,10	male	green	good
3196	2012	Lillgrund	L3	57,60	male	red	good
3197	2012	Lillgrund	L3	59,60	male	red	good
3198	2012	Lillgrund	L3	62,30	male	red	good
3199	2012	Lillgrund	L3	63,80	male	red	good
3200	2012	Lillgrund	L3	66,30	male	red	good
3201	2012	Lillgrund	L3	69,10	male	red	good
3202	2012	Lillgrund	L4	64,70	male	green	good
3203	2012	Lillgrund	L4	49,60	male	red	good
3204	2012	Lillgrund	L4	57,30	male	red	good
3205	2012	Lillgrund	L4	57,60	male	red	good
3206	2012	Lillgrund	L4	60,90	male	red	good
3207	2012	Lillgrund	L4	46,50	female	red	good
3208	2012	Lillgrund	L4	54,80	male	red	good
3209	2012	Lillgrund	L4	60,60	male	red	good
3210	2012	Lillgrund	L4	62,10	male	red	good
3211	2012	Lillgrund	L4	62,60	male	green	good
3212	2012	Lillgrund	L4	63,30	male	green	good
3213	2012	Lillgrund	L4	64,90	male	green	good
3214	2012	Lillgrund	L4	66,50	male	green	good
3215	2012	Lillgrund	L4	68,70	male	green	good
3216	2012	Lillgrund	L4	70,60	male	green	good
3217	2012	Lillgrund	L4	52,30	female	red	reduced
3218	2012	Lillgrund	L4	54,50	male	red	reduced
3219	2012	Lillgrund	L4	41,40	female	red	good
3220	2012	Lillgrund	L4	46,20	female	red	good
3221	2012	Lillgrund	L4	50,20	male	red	good
3222	2012	Lillgrund	L4	55,60	male	red	good
3223	2012	Lillgrund	L4	56,40	male	red	good
3224	2012	Lillgrund	L4	57,20	male	red	good
3225	2012	Lillgrund	L4	61,50	male	red	good
3226	2012	Lillgrund	L4	62,60	male	red	good
3227	2012	Lillgrund	L4	62,80	male	red	good
3228	2012	Lillgrund	L4	63,20	male	red	good
3229	2012	Lillgrund	L4	64,20	male	red	good
3230	2012	Lillgrund	L4	64,50	male	red	good
3231	2012	Lillgrund	L4	63,20	male	green	reduced
3232	2012	Lillgrund	L4	68,80	male	green	reduced
3233	2012	Lillgrund	L4	57,20	male	green	good
3234	2012	Lillgrund	L4	57,20	male	green	good
3235	2012	Lillgrund	L4	58,30	male	green	good
3236	2012	Lillgrund	L4	62,70	male	green	good
3237	2012	Lillgrund	L4	62,90	male	green	good
3238	2012	Lillgrund	L4	68,00	male	green	good
3239	2012	Lillgrund	L4	68,70	male	green	good
3240	2012	Lillgrund	L4	68,80	male	green	good
3241	2012	Lillgrund	L4	45,30	female	red	good

3242	2012	Lillgrund	L4	48,40	female	red	good
3243	2012	Lillgrund	L4	51,90	female	red	good
3244	2012	Lillgrund	L4	55,10	male	red	good
3245	2012	Lillgrund	L4	61,50	male	red	good
3246	2012	Lillgrund	L4	62,50	male	red	good
3247	2012	Lillgrund	L4	62,70	male	red	good
3248	2012	Lillgrund	L4	66,00	male	red	good
3249	2012	Lillgrund	L4	68,10	male	green	reduced
3250	2012	Lillgrund	L4	57,00	male	green	good
3251	2012	Lillgrund	L4	63,40	male	green	good
3252	2012	Lillgrund	L4	65,60	male	green	good
3253	2012	Lillgrund	L4	67,70	male	green	good
3254	2012	Lillgrund	L4	71,00	male	green	good
3255	2012	Lillgrund	L4	71,20	male	green	good
3256	2012	Lillgrund	L4	71,80	male	green	good
3257	2012	Lillgrund	L4	75,50	male	green	good
3258	2012	Lillgrund	L4	75,90	male	green	good
3259	2012	Lillgrund	L4	45,20	male	red	reduced
3260	2012	Lillgrund	L4	57,10	male	red	reduced
3261	2012	Lillgrund	L4	57,90	male	red	reduced
3262	2012	Lillgrund	L4	59,60	male	red	reduced
3263	2012	Lillgrund	L4	62,30	male	red	reduced
3264	2012	Lillgrund	L4	55,70	male	red	good
3265	2012	Lillgrund	L4	57,30	male	red	good
3266	2012	Lillgrund	L4	57,40	male	red	good
3267	2012	Lillgrund	L4	60,90	male	red	good
3268	2012	Lillgrund	L4	73,10	male	red	good
3269	2012	Lillgrund	L4	64,20	male	green	reduced
3270	2012	Lillgrund	L4	68,10	male	green	reduced
3271	2012	Lillgrund	L4	47,30	male	green	good
3272	2012	Lillgrund	L4	52,00	male	green	good
3273	2012	Lillgrund	L4	52,20	male	green	good
3274	2012	Lillgrund	L4	54,10	male	green	good
3275	2012	Lillgrund	L4	55,90	male	green	good
3276	2012	Lillgrund	L4	56,10	male	green	good
3277	2012	Lillgrund	L4	57,30	male	green	good
3278	2012	Lillgrund	L4	60,90	male	green	good
3279	2012	Lillgrund	L4	60,90	male	green	good
3280	2012	Lillgrund	L4	61,90	male	green	good
3281	2012	Lillgrund	L4	62,90	male	green	good
3282	2012	Lillgrund	L4	64,80	male	green	good
3283	2012	Lillgrund	L4	65,00	male	green	good
3284	2012	Lillgrund	L4	65,20	male	green	good
3285	2012	Lillgrund	L4	65,30	male	green	good
3286	2012	Lillgrund	L4	65,50	male	green	good
3287	2012	Lillgrund	L4	65,60	male	green	good
3288	2012	Lillgrund	L4	66,50	male	green	good
3289	2012	Lillgrund	L4	69,20	male	green	good
3290	2012	Lillgrund	L4	69,80	male	green	good
3291	2012	Lillgrund	L4	75,70	male	green	good
3292	2012	Lillgrund	L4	42,20	female	red	reduced
3293	2012	Lillgrund	L4	55,20	male	red	reduced
3294	2012	Lillgrund	L4	56,70	male	red	reduced
3295	2012	Lillgrund	L4	56,90	male	red	reduced
3296	2012	Lillgrund	L4	58,20	male	red	reduced
3297	2012	Lillgrund	L4	59,40	male	red	reduced
3298	2012	Lillgrund	L4	60,10	male	red	reduced
3299	2012	Lillgrund	L4	60,10	male	red	reduced
3300	2012	Lillgrund	L4	67,40	male	red	reduced

3301	2012	Lillgrund	L4	50,40	female	red	good
3302	2012	Lillgrund	L4	55,30	male	red	good
3303	2012	Lillgrund	L4	55,60	male	red	good
3304	2012	Lillgrund	L4	56,80	male	red	good
3305	2012	Lillgrund	L4	57,30	male	red	good
3306	2012	Lillgrund	L4	60,60	male	red	good
3307	2012	Lillgrund	L4	62,70	male	red	good
3308	2012	Lillgrund	L4	63,10	male	red	good
3309	2012	Lillgrund	L4	63,40	male	red	good
3310	2012	Lillgrund	L4	63,80	male	red	good
3311	2012	Lillgrund	L4	64,30	male	red	good
3312	2012	Lillgrund	L4	66,20	male	red	good
3313	2012	Lillgrund	L4	66,60	male	red	good
3314	2012	Lillgrund	L4	67,40	male	red	good
3315	2012	Lillgrund	L4	69,10	male	red	good
3316	2012	Lillgrund	L4	69,70	male	red	good
3317	2012	Lillgrund	L5	59,00	male	red	reduced
3318	2012	Lillgrund	L5	47,90	male	red	good
3319	2012	Lillgrund	L5	54,90	male	red	good
3320	2012	Lillgrund	L5	55,00	male	red	good
3321	2012	Lillgrund	L5	34,00	female	green	reduced
3322	2012	Lillgrund	L5	50,40	male	green	good
3323	2012	Lillgrund	L5	55,00	male	green	good
3324	2012	Lillgrund	L5	55,70	male	green	good
3325	2012	Lillgrund	L5	61,10	male	green	good
3326	2012	Lillgrund	L5	61,70	male	green	good
3327	2012	Lillgrund	L5	66,90	male	green	good
3328	2012	Lillgrund	L5	46,80	male	red	reduced
3329	2012	Lillgrund	L5	52,30	male	red	good
3330	2012	Lillgrund	L5	53,90	male	red	good
3331	2012	Lillgrund	L5	53,90	male	red	good
3332	2012	Lillgrund	L5	54,90	male	red	good
3333	2012	Lillgrund	L5	55,00	male	red	good
3334	2012	Lillgrund	L5	57,00	male	red	good
3335	2012	Lillgrund	L5	57,80	male	red	good
3336	2012	Lillgrund	L5	60,10	male	green	reduced
3337	2012	Lillgrund	L5	71,30	male	green	reduced
3338	2012	Lillgrund	L5	56,10	male	green	good
3339	2012	Lillgrund	L5	58,00	male	green	good
3340	2012	Lillgrund	L5	58,30	male	green	good
3341	2012	Lillgrund	L5	59,00	male	green	good
3342	2012	Lillgrund	L5	59,40	male	green	good
3343	2012	Lillgrund	L5	59,40	male	green	good
3344	2012	Lillgrund	L5	59,50	male	green	good
3345	2012	Lillgrund	L5	61,20	male	green	good
3346	2012	Lillgrund	L5	61,20	male	green	good
3347	2012	Lillgrund	L5	65,10	male	green	good
3348	2012	Lillgrund	L5	65,20	male	green	good
3349	2012	Lillgrund	L5	67,80	male	green	good
3350	2012	Lillgrund	L5	68,40	male	green	good
3351	2012	Lillgrund	L5	68,70	male	green	good
3352	2012	Lillgrund	L5	69,90	male	green	good
3353	2012	Lillgrund	L5	70,50	male	green	good
3354	2012	Lillgrund	L5	51,10	male	red	reduced
3355	2012	Lillgrund	L5	57,90	male	red	reduced
3356	2012	Lillgrund	L5	58,80	male	red	reduced
3357	2012	Lillgrund	L5	58,80	male	red	reduced
3358	2012	Lillgrund	L5	59,70	male	red	reduced
3359	2012	Lillgrund	L5	63,50	male	red	reduced

3360	2012	Lillgrund	L5	46,00	female	red	good
3361	2012	Lillgrund	L5	48,30	female	red	good
3362	2012	Lillgrund	L5	51,10	male	red	good
3363	2012	Lillgrund	L5	54,90	male	red	good
3364	2012	Lillgrund	L5	55,00	male	red	good
3365	2012	Lillgrund	L5	55,60	male	red	good
3366	2012	Lillgrund	L5	58,50	male	red	good
3367	2012	Lillgrund	L5	59,20	male	red	good
3368	2012	Lillgrund	L5	60,70	male	red	good
3369	2012	Lillgrund	L5	63,00	male	red	good
3370	2012	Lillgrund	L5	63,50	male	red	good
3371	2012	Lillgrund	L5	66,20	male	red	good
3372	2012	Lillgrund	L5	67,60	male	red	good
3373	2012	Lillgrund	L5	59,60	male	green	good
3374	2012	Lillgrund	L5	42,40	female	red	good
3375	2012	Lillgrund	L5	52,70	male	red	good
3376	2012	Lillgrund	L5	60,30	male	red	good
3377	2012	Lillgrund	L5	61,20	male	red	good
3378	2012	Lillgrund	L5	61,40	male	red	good
3379	2012	Lillgrund	L5	63,80	male	red	good
3380	2012	Lillgrund	L5	64,60	male	red	good
3381	2012	Lillgrund	L5	65,10	male	red	good
3382	2012	Lillgrund	L5	59,60	male	green	good
3383	2012	Lillgrund	L5	43,20	female	red	good
3384	2012	Lillgrund	L5	53,40	male	green	reduced
3385	2012	Lillgrund	L5	60,10	male	green	reduced
3386	2012	Lillgrund	L5	59,30	male	green	good
3387	2012	Lillgrund	L5	61,10	male	green	good
3388	2012	Lillgrund	L5	68,90	male	green	good
3389	2012	Lillgrund	L5	54,70	male	red	reduced
3390	2012	Lillgrund	L5	60,20	male	red	reduced
3391	2012	Lillgrund	L5	52,70	male	red	good
3392	2012	Lillgrund	L5	54,90	male	red	good
3393	2012	Lillgrund	L5	58,70	male	red	good
3394	2012	Lillgrund	L5	61,10	male	red	good
3395	2012	Lillgrund	L5	63,30	male	red	good
3396	2012	Lillgrund	L5	63,80	male	red	good
3397	2012	Lillgrund	L5	66,00	male	red	good
3398	2012	Lillgrund	L5	66,50	male	red	good
3399	2012	Lillgrund	L5	70,90	male	red	good
3400	2012	Lillgrund	L6	59,20	male	red	reduced
3401	2012	Lillgrund	L6	59,20	male	red	reduced
3402	2012	Lillgrund	L6	50,10	male	red	good
3403	2012	Lillgrund	L6	55,50	male	red	good
3404	2012	Lillgrund	L6	58,20	male	red	good
3405	2012	Lillgrund	L6	60,80	male	red	good
3406	2012	Lillgrund	L6	61,50	male	red	good
3407	2012	Lillgrund	L6	63,10	male	red	good
3408	2012	Lillgrund	L6	65,20	male	red	good
3409	2012	Lillgrund	L6	48,00	male	green	reduced
3410	2012	Lillgrund	L6	50,30	male	green	reduced
3411	2012	Lillgrund	L6	52,20	male	green	reduced
3412	2012	Lillgrund	L6	51,90	male	red	reduced
3413	2012	Lillgrund	L6	53,30	male	red	reduced
3414	2012	Lillgrund	L6	55,30	male	red	reduced
3415	2012	Lillgrund	L6	43,00	female	red	good
3416	2012	Lillgrund	L6	51,60	female	red	good
3417	2012	Lillgrund	L6	53,90	male	red	good
3418	2012	Lillgrund	L6	54,00	male	red	good

3419	2012	Lillgrund	L6	55,00	male	red	good
3420	2012	Lillgrund	L6	55,90	male	red	good
3421	2012	Lillgrund	L6	59,00	male	red	good
3422	2012	Lillgrund	L6	67,40	male	green	reduced
3423	2012	Lillgrund	L6	65,20	male	green	good
3424	2012	Lillgrund	L6	68,20	male	green	good
3425	2012	Lillgrund	L6	53,80	male	red	reduced
3426	2012	Lillgrund	L6	57,60	male	red	reduced
3427	2012	Lillgrund	L6	58,70	male	red	reduced
3428	2012	Lillgrund	L6	62,10	male	red	reduced
3429	2012	Lillgrund	L6	62,20	male	red	reduced
3430	2012	Lillgrund	L6	65,90	male	red	reduced
3431	2012	Lillgrund	L6	43,40	female	red	good
3432	2012	Lillgrund	L6	47,70	male	red	good
3433	2012	Lillgrund	L6	49,00	female	red	good
3434	2012	Lillgrund	L6	49,20	female	red	good
3435	2012	Lillgrund	L6	53,40	male	red	good
3436	2012	Lillgrund	L6	57,70	male	red	good
3437	2012	Lillgrund	L6	57,80	male	red	good
3438	2012	Lillgrund	L6	57,90	male	red	good
3439	2012	Lillgrund	L6	59,00	male	red	good
3440	2012	Lillgrund	L6	59,60	male	red	good
3441	2012	Lillgrund	L6	60,80	male	red	good
3442	2012	Lillgrund	L6	61,30	male	red	good
3443	2012	Lillgrund	L6	62,00	male	red	good
3444	2012	Lillgrund	L6	62,30	male	red	good
3445	2012	Lillgrund	L6	63,60	male	red	good
3446	2012	Lillgrund	L6	63,80	male	red	good
3447	2012	Lillgrund	L6	64,50	male	red	good
3448	2012	Lillgrund	L6	64,80	male	red	good
3449	2012	Lillgrund	L6	70,00	male	red	good
3450	2012	Lillgrund	L6	57,60	male	red	good
3451	2012	Lillgrund	L6	64,40	male	green	reduced
3452	2012	Lillgrund	L6	58,50	male	green	good
3453	2012	Lillgrund	L6	60,70	male	green	good
3454	2012	Lillgrund	L6	64,90	male	green	good
3455	2012	Lillgrund	L6	64,90	male	green	good
3456	2012	Lillgrund	L6	47,30	female	red	reduced
3457	2012	Lillgrund	L6	61,40	male	red	reduced
3458	2012	Lillgrund	L6	67,20	male	red	reduced
3459	2012	Lillgrund	L6	51,70	male	red	good
3460	2012	Lillgrund	L6	53,30	female	red	good
3461	2012	Lillgrund	L6	55,80	male	red	good
3462	2012	Lillgrund	L6	59,00	male	red	good
3463	2012	Lillgrund	L6	60,90	male	red	good
3464	2012	Lillgrund	L6	61,40	male	red	good
3465	2012	Lillgrund	L6	61,50	male	red	good
3466	2012	Lillgrund	L6	61,70	male	red	good
3467	2012	Lillgrund	L6	61,70	male	red	good
3468	2012	Lillgrund	L6	62,50	male	red	good
3469	2012	Lillgrund	L6	62,70	male	red	good
3470	2012	Lillgrund	L6	63,80	male	red	good
3471	2012	Lillgrund	L6	64,30	male	red	good
3472	2012	Lillgrund	L6	65,70	male	red	good
3473	2012	Lillgrund	L6	62,80	male	green	reduced
3474	2012	Lillgrund	L6	62,20	male	green	good
3475	2012	Lillgrund	L6	62,90	male	green	good
3476	2012	Lillgrund	L6	63,50	male	green	good
3477	2012	Lillgrund	L6	64,00	male	green	good

3478	2012	Lillgrund	L6	64,50	male	green	good
3479	2012	Lillgrund	L6	64,60	male	green	good
3480	2012	Lillgrund	L6	66,40	male	green	good
3481	2012	Lillgrund	L6	67,30	male	green	good
3482	2012	Lillgrund	L6	67,30	male	green	good
3483	2012	Lillgrund	L6	68,90	male	green	good
3484	2012	Lillgrund	L6	69,90	male	green	good
3485	2012	Lillgrund	L6	70,00	male	green	good
3486	2012	Lillgrund	L6	71,20	male	green	good
3487	2012	Lillgrund	L6	51,30	female	red	good
3488	2012	Lillgrund	L6	59,00	male	red	good
3489	2012	Lillgrund	L6	62,20	male	red	good
3490	2012	Lillgrund	L6	63,70	male	red	good
3491	2012	Lillgrund	L6	65,50	male	red	good
3492	2012	Lillgrund	L6	55,50	male	green	reduced
3493	2012	Lillgrund	L6	66,70	male	green	reduced
3494	2012	Lillgrund	L6	68,00	male	green	reduced
3495	2012	Lillgrund	L6	53,60	male	green	good
3496	2012	Lillgrund	L6	54,80	male	green	good
3497	2012	Lillgrund	L6	58,20	male	green	good
3498	2012	Lillgrund	L6	59,50	male	green	good
3499	2012	Lillgrund	L6	60,10	male	green	good
3500	2012	Lillgrund	L6	60,20	male	green	good
3501	2012	Lillgrund	L6	61,70	male	green	good
3502	2012	Lillgrund	L6	65,50	male	green	good
3503	2012	Lillgrund	L6	65,60	male	green	good
3504	2012	Lillgrund	L6	66,60	male	green	good
3505	2012	Lillgrund	L6	67,50	male	green	good
3506	2012	Lillgrund	L6	70,40	male	green	good
3507	2012	Lillgrund	L6	73,10	male	green	good
3508	2012	Lillgrund	L6	61,20	male	red	reduced
3509	2012	Lillgrund	L6	62,30	male	red	reduced
3510	2012	Lillgrund	L6	62,50	male	red	reduced
3511	2012	Lillgrund	L6	64,20	male	red	reduced
3512	2012	Lillgrund	L6	43,00	female	red	good
3513	2012	Lillgrund	L6	52,20	male	red	good
3514	2012	Lillgrund	L6	54,10	male	red	good
3515	2012	Lillgrund	L6	55,30	male	red	good
3516	2012	Lillgrund	L6	59,20	male	red	good
3517	2012	Lillgrund	L6	60,40	male	red	good
3518	2012	Lillgrund	L6	61,30	male	red	good
3519	2012	Lillgrund	L6	61,70	male	red	good
3520	2012	Lillgrund	L6	61,90	male	red	good
3521	2012	Lillgrund	L6	62,10	male	red	good
3522	2012	Lillgrund	L6	63,20	male	red	good
3523	2012	Lillgrund	L6	63,90	male	red	good
3524	2012	Lillgrund	L6	64,60	male	red	good
3525	2012	Lillgrund	L6	64,80	male	red	good
3526	2012	Lillgrund	L7	58,20	male	green	reduced
3527	2012	Lillgrund	L7	48,70	female	red	reduced
3528	2012	Lillgrund	L7	50,80	male	red	good
3529	2012	Lillgrund	L7	52,90	male	red	good
3530	2012	Lillgrund	L7	53,70	male	red	good
3531	2012	Lillgrund	L7	61,70	male	red	good
3532	2012	Lillgrund	L7	62,00	male	red	good
3533	2012	Lillgrund	L7	62,60	male	red	good
3534	2012	Lillgrund	L7	78,10	male	red	good
3535	2012	Lillgrund	L7	60,10	male	green	good
3536	2012	Lillgrund	L7	56,90	male	red	reduced

3537	2012	Lillgrund	L7	40,80	female	red	good
3538	2012	Lillgrund	L7	47,50	male	red	good
3539	2012	Lillgrund	L7	49,90	male	red	good
3540	2012	Lillgrund	L7	55,90	male	red	good
3541	2012	Lillgrund	L7	57,20	male	red	good
3542	2012	Lillgrund	L7	57,40	male	red	good
3543	2012	Lillgrund	L7	57,50	male	red	good
3544	2012	Lillgrund	L7	57,90	male	red	good
3545	2012	Lillgrund	L7	54,80	male	green	good
3546	2012	Lillgrund	L7	57,50	male	green	good
3547	2012	Lillgrund	L7	61,00	male	green	good
3548	2012	Lillgrund	L7	61,40	male	green	good
3549	2012	Lillgrund	L7	62,80	male	green	good
3550	2012	Lillgrund	L7	65,90	male	green	good
3551	2012	Lillgrund	L7	74,10	male	green	good
3552	2012	Lillgrund	L7	79,10	male	green	good
3553	2012	Lillgrund	L7	64,80	male	red	reduced
3554	2012	Lillgrund	L7	51,60	female	red	good
3555	2012	Lillgrund	L7	58,90	male	red	good
3556	2012	Lillgrund	L7	61,70	male	red	good
3557	2012	Lillgrund	L7	63,20	male	red	good
3558	2012	Lillgrund	L7	63,30	male	red	good
3559	2012	Lillgrund	L7	68,30	male	red	good
3560	2012	Lillgrund	L7	55,30	male	green	reduced
3561	2012	Lillgrund	L7	72,50	male	green	good
3562	2012	Lillgrund	L7	50,20	female	red	reduced
3563	2012	Lillgrund	L7	59,00	male	red	reduced
3564	2012	Lillgrund	L7	61,80	male	red	reduced
3565	2012	Lillgrund	L7	61,90	male	red	reduced
3566	2012	Lillgrund	L7	67,30	male	red	reduced
3567	2012	Lillgrund	L7	45,10	female	red	good
3568	2012	Lillgrund	L7	57,10	male	red	good
3569	2012	Lillgrund	L7	57,10	male	red	good
3570	2012	Lillgrund	L7	57,90	male	red	good
3571	2012	Lillgrund	L7	60,30	male	red	good
3572	2012	Lillgrund	L7	60,50	male	red	good
3573	2012	Lillgrund	L7	63,30	male	red	good
3574	2012	Lillgrund	L7	64,00	male	red	good
3575	2012	Lillgrund	L7	66,10	male	red	good
3576	2012	Lillgrund	L7	68,50	male	red	good
3577	2012	Lillgrund	L7	54,60	male	green	good
3578	2012	Lillgrund	L7	57,00	male	green	good
3579	2012	Lillgrund	L7	60,70	male	green	good
3580	2012	Lillgrund	L7	62,10	male	green	good
3581	2012	Lillgrund	L7	65,30	male	green	good
3582	2012	Lillgrund	L7	65,90	male	green	good
3583	2012	Lillgrund	L7	67,90	male	green	good
3584	2012	Lillgrund	L7	69,80	male	green	good
3585	2012	Lillgrund	L7	75,70	male	green	good
3586	2012	Lillgrund	L7	61,70	male	red	reduced
3587	2012	Lillgrund	L7	67,10	male	red	reduced
3588	2012	Lillgrund	L7	42,40	female	red	good
3589	2012	Lillgrund	L7	43,40	female	red	good
3590	2012	Lillgrund	L7	46,80	female	red	good
3591	2012	Lillgrund	L7	58,10	male	red	good
3592	2012	Lillgrund	L7	58,80	male	red	good
3593	2012	Lillgrund	L7	60,00	male	red	good
3594	2012	Lillgrund	L7	62,00	male	red	good
3595	2012	Lillgrund	L7	66,40	male	green	reduced

3596	2012	Lillgrund	L7	55,40	male	green	good
3597	2012	Lillgrund	L7	55,80	male	green	good
3598	2012	Lillgrund	L7	58,10	male	green	good
3599	2012	Lillgrund	L7	61,20	male	green	good
3600	2012	Lillgrund	L7	61,50	male	green	good
3601	2012	Lillgrund	L7	64,20	male	green	good
3602	2012	Lillgrund	L7	64,60	male	green	good
3603	2012	Lillgrund	L7	64,80	male	green	good
3604	2012	Lillgrund	L7	65,30	male	green	good
3605	2012	Lillgrund	L7	68,20	male	green	good
3606	2012	Lillgrund	L7	69,10	male	green	good
3607	2012	Lillgrund	L7	63,60	male	red	reduced
3608	2012	Lillgrund	L7	66,10	male	red	reduced
3609	2012	Lillgrund	L7	55,20	male	red	good
3610	2012	Lillgrund	L7	59,00	male	red	good
3611	2012	Lillgrund	L7	60,10	male	red	good
3612	2012	Lillgrund	L7	60,40	male	red	good
3613	2012	Lillgrund	L7	62,40	male	red	good
3614	2012	Lillgrund	L7	63,00	male	red	good
3615	2012	Lillgrund	L7	63,10	male	red	good
3616	2012	Lillgrund	L7	67,50	male	red	good
3617	2012	Lillgrund	L8	61,10	male	red	reduced
3618	2012	Lillgrund	L8	66,20	male	red	reduced
3619	2012	Lillgrund	L8	51,40	male	red	good
3620	2012	Lillgrund	L8	56,10	male	red	good
3621	2012	Lillgrund	L8	57,20	male	red	good
3622	2012	Lillgrund	L8	58,70	male	red	good
3623	2012	Lillgrund	L8	63,90	male	red	good
3624	2012	Lillgrund	L8	63,90	male	red	good
3625	2012	Lillgrund	L8	62,80	male	green	good
3626	2012	Lillgrund	L8	61,50	male	red	reduced
3627	2012	Lillgrund	L8	54,20	male	red	good
3628	2012	Lillgrund	L8	55,20	male	red	good
3629	2012	Lillgrund	L8	55,70	male	red	good
3630	2012	Lillgrund	L8	56,60	male	red	good
3631	2012	Lillgrund	L8	62,10	male	green	reduced
3632	2012	Lillgrund	L8	68,50	male	green	reduced
3633	2012	Lillgrund	L8	57,50	male	green	good
3634	2012	Lillgrund	L8	60,50	male	green	good
3635	2012	Lillgrund	L8	62,40	male	green	good
3636	2012	Lillgrund	L8	64,10	male	green	good
3637	2012	Lillgrund	L8	66,70	male	green	good
3638	2012	Lillgrund	L8	68,30	male	green	good
3639	2012	Lillgrund	L8	69,20	male	green	good
3640	2012	Lillgrund	L8	69,30	male	green	good
3641	2012	Lillgrund	L8	66,70	male	red	reduced
3642	2012	Lillgrund	L8	65,60	male	red	good
3643	2012	Lillgrund	L8	68,10	male	red	good
3644	2012	Lillgrund	L8	58,20	male	green	good
3645	2012	Lillgrund	L8	60,10	male	green	good
3646	2012	Lillgrund	L8	60,70	male	green	good
3647	2012	Lillgrund	L8	62,90	male	green	good
3648	2012	Lillgrund	L8	63,80	male	green	good
3649	2012	Lillgrund	L8	66,60	male	green	good
3650	2012	Lillgrund	L8	70,90	male	green	good
3651	2012	Lillgrund	L8	72,50	male	green	good
3652	2012	Lillgrund	L8	50,60	female	red	reduced
3653	2012	Lillgrund	L8	60,50	male	red	reduced
3654	2012	Lillgrund	L8	60,30	male	red	good

3655	2012	Lillgrund	L8	63,20	male	red	good
3656	2012	Lillgrund	L8	64,20	male	red	good
3657	2012	Lillgrund	L8	67,80	male	red	good
3658	2012	Lillgrund	L8	70,30	male	red	good
3659	2012	Lillgrund	L8	51,70	male	green	reduced
3660	2012	Lillgrund	L8	62,20	male	green	reduced
3661	2012	Lillgrund	L8	71,30	male	green	reduced
3662	2012	Lillgrund	L8	58,80	male	green	good
3663	2012	Lillgrund	L8	60,20	male	green	good
3664	2012	Lillgrund	L8	60,60	male	green	good
3665	2012	Lillgrund	L8	61,30	male	green	good
3666	2012	Lillgrund	L8	65,50	male	green	good
3667	2012	Lillgrund	L8	65,70	male	green	good
3668	2012	Lillgrund	L8	67,90	male	green	good
3669	2012	Lillgrund	L8	69,30	male	green	good
3670	2012	Lillgrund	L8	69,40	male	green	good
3671	2012	Lillgrund	L8	70,00	male	green	good
3672	2012	Lillgrund	L8	70,80	male	green	good
3673	2012	Lillgrund	L8	57,60	male	red	reduced
3674	2012	Lillgrund	L8	60,70	male	red	reduced
3675	2012	Lillgrund	L8	64,10	male	red	reduced
3676	2012	Lillgrund	L8	56,20	male	red	good
3677	2012	Lillgrund	L8	57,00	male	red	good
3678	2012	Lillgrund	L8	57,80	male	red	good
3679	2012	Lillgrund	L8	58,10	male	red	good
3680	2012	Lillgrund	L8	60,10	male	red	good
3681	2012	Lillgrund	L8	60,30	male	red	good
3682	2012	Lillgrund	L8	61,40	male	red	good
3683	2012	Lillgrund	L8	62,20	male	red	good
3684	2012	Lillgrund	L8	63,20	male	red	good
3685	2012	Lillgrund	L8	63,60	male	red	good
3686	2012	Lillgrund	L8	63,80	male	red	good
3687	2012	Lillgrund	L8	64,90	male	red	good
3688	2012	Lillgrund	L8	66,70	male	red	good
3689	2012	Lillgrund	L8	66,80	male	red	good
3690	2012	Lillgrund	L8	60,60	male	green	reduced
3691	2012	Lillgrund	L8	61,40	male	green	reduced
3692	2012	Lillgrund	L8	51,90	male	green	good
3693	2012	Lillgrund	L8	52,60	male	green	good
3694	2012	Lillgrund	L8	52,70	male	green	good
3695	2012	Lillgrund	L8	54,50	male	green	good
3696	2012	Lillgrund	L8	57,40	male	green	good
3697	2012	Lillgrund	L8	58,30	male	green	good
3698	2012	Lillgrund	L8	59,80	male	green	good
3699	2012	Lillgrund	L8	62,00	male	green	good
3700	2012	Lillgrund	L8	62,40	male	green	good
3701	2012	Lillgrund	L8	63,70	male	green	good
3702	2012	Lillgrund	L8	63,80	male	green	good
3703	2012	Lillgrund	L8	63,90	male	green	good
3704	2012	Lillgrund	L8	64,50	male	green	good
3705	2012	Lillgrund	L8	65,20	male	green	good
3706	2012	Lillgrund	L8	65,40	male	green	good
3707	2012	Lillgrund	L8	67,90	male	green	good
3708	2012	Lillgrund	L8	68,00	male	green	good
3709	2012	Lillgrund	L8	69,10	male	green	good
3710	2012	Lillgrund	L8	72,90	male	green	good
3711	2012	Lillgrund	L8	73,30	male	green	good
3712	2012	Lillgrund	L8	75,20	male	green	good
3713	2012	Lillgrund	L8	59,40	male	red	reduced

3714	2012	Lillgrund	L8	59,90	male	red	reduced
3715	2012	Lillgrund	L8	60,20	male	red	reduced
3716	2012	Lillgrund	L8	64,10	male	red	reduced
3717	2012	Lillgrund	L8	49,80	female	red	good
3718	2012	Lillgrund	L8	55,60	male	red	good
3719	2012	Lillgrund	L8	58,10	male	red	good
3720	2012	Lillgrund	L8	58,10	male	red	good
3721	2012	Lillgrund	L8	58,50	male	red	good
3722	2012	Lillgrund	L8	59,00	male	red	good
3723	2012	Lillgrund	L8	59,10	male	red	good
3724	2012	Lillgrund	L8	59,30	male	red	good
3725	2012	Lillgrund	L8	59,40	male	red	good
3726	2012	Lillgrund	L8	59,50	male	red	good
3727	2012	Lillgrund	L8	61,70	male	red	good
3728	2012	Lillgrund	L8	63,90	male	red	good
3729	2012	Lillgrund	L8	64,40	male	red	good
3730	2012	Lillgrund	L8	65,20	male	red	good
3731	2012	Lillgrund	L8	65,50	male	red	good
3732	2012	Lillgrund	L8	67,30	male	red	good
3733	2012	Lillgrund	L9	60,90	male	green	good
3734	2012	Lillgrund	L9	56,30	male	red	good
3735	2012	Lillgrund	L9	57,50	male	red	good
3736	2012	Lillgrund	L9	57,70	male	red	good
3737	2012	Lillgrund	L9	58,70	male	red	good
3738	2012	Lillgrund	L9	59,50	male	red	good
3739	2012	Lillgrund	L9	62,10	male	red	good
3740	2012	Lillgrund	L9	58,60	male	green	good
3741	2012	Lillgrund	L9	67,80	male	green	good
3742	2012	Lillgrund	L9	46,90	male	red	good
3743	2012	Lillgrund	L9	49,10	male	red	good
3744	2012	Lillgrund	L9	52,80	male	red	good
3745	2012	Lillgrund	L9	53,80	male	red	good
3746	2012	Lillgrund	L9	54,10	male	red	good
3747	2012	Lillgrund	L9	54,80	male	red	good
3748	2012	Lillgrund	L9	55,00	male	red	good
3749	2012	Lillgrund	L9	58,00	male	red	good
3750	2012	Lillgrund	L9	59,00	male	red	good
3751	2012	Lillgrund	L9	68,10	male	green	good
3752	2012	Lillgrund	L9	68,40	male	green	good
3753	2012	Lillgrund	L9	70,30	male	green	good
3754	2012	Lillgrund	L9	72,80	male	green	good
3755	2012	Lillgrund	L9	58,00	male	red	reduced
3756	2012	Lillgrund	L9	43,30	female	red	good
3757	2012	Lillgrund	L9	52,30	male	red	good
3758	2012	Lillgrund	L9	52,70	male	red	good
3759	2012	Lillgrund	L9	59,00	male	red	good
3760	2012	Lillgrund	L9	59,30	male	red	good
3761	2012	Lillgrund	L9	60,10	male	red	good
3762	2012	Lillgrund	L9	61,60	male	red	good
3763	2012	Lillgrund	L9	62,50	male	red	good
3764	2012	Lillgrund	L9	62,80	male	red	good
3765	2012	Lillgrund	L9	64,00	male	red	good
3766	2012	Lillgrund	L9	64,10	male	red	good
3767	2012	Lillgrund	L9	79,30	male	red	good
3768	2012	Lillgrund	L9	58,00	male	green	good
3769	2012	Lillgrund	L9	65,70	male	green	good
3770	2012	Lillgrund	L9	65,90	male	green	good
3771	2012	Lillgrund	L9	57,40	male	red	reduced
3772	2012	Lillgrund	L9	60,40	male	red	reduced

3773	2012	Lillgrund	L9	61,70	male	red	reduced
3774	2012	Lillgrund	L9	53,30	male	red	good
3775	2012	Lillgrund	L9	54,90	female	red	good
3776	2012	Lillgrund	L9	55,70	male	red	good
3777	2012	Lillgrund	L9	56,40	male	red	good
3778	2012	Lillgrund	L9	56,90	female	red	good
3779	2012	Lillgrund	L9	57,60	male	red	good
3780	2012	Lillgrund	L9	57,80	male	red	good
3781	2012	Lillgrund	L9	58,80	male	red	good
3782	2012	Lillgrund	L9	59,80	male	red	good
3783	2012	Lillgrund	L9	62,20	male	red	good
3784	2012	Lillgrund	L9	61,00	male	green	reduced
3785	2012	Lillgrund	L9	53,40	male	green	good
3786	2012	Lillgrund	L9	69,80	male	green	good
3787	2012	Lillgrund	L9	57,30	male	red	reduced
3788	2012	Lillgrund	L9	56,40	male	red	good
3789	2012	Lillgrund	L9	58,30	male	red	good
3790	2012	Lillgrund	L9	58,70	male	red	good
3791	2012	Lillgrund	L9	59,70	male	red	good
3792	2012	Lillgrund	L9	69,60	male	green	reduced
3793	2012	Lillgrund	L9	55,60	male	green	good
3794	2012	Lillgrund	L9	62,80	male	green	good
3795	2012	Lillgrund	L9	63,10	male	green	good
3796	2012	Lillgrund	L9	63,20	male	green	good
3797	2012	Lillgrund	L9	64,70	male	green	good
3798	2012	Lillgrund	L9	66,90	male	green	good
3799	2012	Lillgrund	L9	67,20	male	green	good
3800	2012	Lillgrund	L9	69,40	male	green	good
3801	2012	Lillgrund	L9	48,40	female	red	reduced
3802	2012	Lillgrund	L9	59,10	male	red	good
3803	2012	Lillgrund	L9	61,60	male	red	good
3804	2012	Lillgrund	L9	62,80	male	red	good
3805	2012	Lillgrund	L9	65,60	male	red	good
3806	2012	Lillgrund	L9	66,50	male	red	good
3807	2012	Lillgrund	L9	69,10	male	red	good
3808	2012	Lillgrund	L9	69,50	male	red	good
3809	2012	Bredgrund	B1	58,30	male	green	good
3810	2012	Bredgrund	B1	58,60	male	green	good
3811	2012	Bredgrund	B1	62,50	male	red	reduced
3812	2012	Bredgrund	B1	55,90	male	red	good
3813	2012	Bredgrund	B1	60,40	male	red	good
3814	2012	Bredgrund	B1	65,10	male	green	good
3815	2012	Bredgrund	B1	62,00	male	red	reduced
3816	2012	Bredgrund	B1	65,50	male	red	good
3817	2012	Bredgrund	B1	63,60	male	green	reduced
3818	2012	Bredgrund	B1	59,50	male	green	good
3819	2012	Bredgrund	B1	61,20	male	green	good
3820	2012	Bredgrund	B1	55,50	male	red	reduced
3821	2012	Bredgrund	B1	42,10	female	red	good
3822	2012	Bredgrund	B1	56,30	male	red	good
3823	2012	Bredgrund	B1	66,50	male	green	reduced
3824	2012	Bredgrund	B1	67,10	male	green	good
3825	2012	Bredgrund	B1	56,20	male	red	reduced
3826	2012	Bredgrund	B1	60,60	male	red	good
3827	2012	Bredgrund	B1	62,90	male	red	good
3828	2012	Bredgrund	B1	63,20	male	red	good
3829	2012	Bredgrund	B1	66,50	male	red	good
3830	2012	Bredgrund	B1	66,90	male	green	good
3831	2012	Bredgrund	B1	71,50	male	green	good

3832	2012	Bredgrund	B1	57,50	male	red	reduced
3833	2012	Bredgrund	B1	58,40	male	red	good
3834	2012	Bredgrund	B1	59,10	male	red	good
3835	2012	Bredgrund	B1	60,40	male	red	good
3836	2012	Bredgrund	B1	60,80	male	red	good
3837	2012	Bredgrund	B1	62,40	male	red	good
3838	2012	Bredgrund	B1	64,00	male	red	good
3839	2012	Bredgrund	B10	62,90	male	red	good
3840	2012	Bredgrund	B10	68,90	male	green	good
3841	2012	Bredgrund	B10	58,60	male	red	reduced
3842	2012	Bredgrund	B10	48,70	female	red	good
3843	2012	Bredgrund	B10	54,50	male	red	good
3844	2012	Bredgrund	B10	65,10	male	red	reduced
3845	2012	Bredgrund	B10	49,60	female	red	good
3846	2012	Bredgrund	B10	58,40	male	red	good
3847	2012	Bredgrund	B10	60,10	male	red	good
3848	2012	Bredgrund	B10	61,00	male	red	good
3849	2012	Bredgrund	B10	59,10	male	green	good
3850	2012	Bredgrund	B10	66,20	male	green	good
3851	2012	Bredgrund	B10	70,00	male	green	good
3852	2012	Bredgrund	B10	59,10	male	green	good
3853	2012	Bredgrund	B10	62,60	male	green	good
3854	2012	Bredgrund	B10	63,50	male	red	reduced
3855	2012	Bredgrund	B10	63,90	male	red	reduced
3856	2012	Bredgrund	B10	71,80	male	red	reduced
3857	2012	Bredgrund	B10	58,50	male	red	good
3858	2012	Bredgrund	B10	59,20	male	red	good
3859	2012	Bredgrund	B10	59,50	male	red	good
3860	2012	Bredgrund	B2	49,40	male	red	reduced
3861	2012	Bredgrund	B2	58,60	male	red	reduced
3862	2012	Bredgrund	B2	73,70	male	red	good
3863	2012	Bredgrund	B2	56,70	male	red	good
3864	2012	Bredgrund	B2	59,50	male	red	good
3865	2012	Bredgrund	B2	62,80	male	red	good
3866	2012	Bredgrund	B2	73,30	male	red	good
3867	2012	Bredgrund	B2	57,10	male	red	reduced
3868	2012	Bredgrund	B2	44,30	female	red	good
3869	2012	Bredgrund	B2	55,00	male	red	good
3870	2012	Bredgrund	B2	65,00	male	green	reduced
3871	2012	Bredgrund	B2	56,00	male	green	good
3872	2012	Bredgrund	B2	63,00	male	green	good
3873	2012	Bredgrund	B2	68,90	male	green	good
3874	2012	Bredgrund	B2	70,00	male	green	good
3875	2012	Bredgrund	B2	41,30	female	red	good
3876	2012	Bredgrund	B2	67,60	male	red	good
3877	2012	Bredgrund	B2	55,40	male	green	good
3878	2012	Bredgrund	B2	62,80	male	green	good
3879	2012	Bredgrund	B2	60,80	male	red	reduced
3880	2012	Bredgrund	B2	61,60	male	red	good
3881	2012	Bredgrund	B2	64,70	male	red	good
3882	2012	Bredgrund	B2	57,90	male	green	good
3883	2012	Bredgrund	B2	60,20	male	green	good
3884	2012	Bredgrund	B2	64,80	male	green	good
3885	2012	Bredgrund	B2	65,20	male	green	good
3886	2012	Bredgrund	B2	65,50	male	green	good
3887	2012	Bredgrund	B2	67,00	male	green	good
3888	2012	Bredgrund	B2	57,20	male	red	reduced
3889	2012	Bredgrund	B2	57,40	male	red	reduced
3890	2012	Bredgrund	B2	57,70	male	red	reduced

3891	2012	Bredgrund	B2	60,90	male	red	reduced
3892	2012	Bredgrund	B2	67,10	male	red	reduced
3893	2012	Bredgrund	B2	54,20	male	red	good
3894	2012	Bredgrund	B2	54,40	male	red	good
3895	2012	Bredgrund	B2	57,10	male	red	good
3896	2012	Bredgrund	B2	57,40	male	red	good
3897	2012	Bredgrund	B2	58,00	male	red	good
3898	2012	Bredgrund	B2	58,40	male	red	good
3899	2012	Bredgrund	B2	60,70	male	red	good
3900	2012	Bredgrund	B3	43,50	male	green	reduced
3901	2012	Bredgrund	B3	57,30	male	green	good
3902	2012	Bredgrund	B3	58,70	male	green	good
3903	2012	Bredgrund	B3	59,00	male	green	good
3904	2012	Bredgrund	B3	66,00	male	green	good
3905	2012	Bredgrund	B3	39,20	female	red	good
3906	2012	Bredgrund	B3	49,90	female	red	good
3907	2012	Bredgrund	B3	53,30	male	red	good
3908	2012	Bredgrund	B3	59,90	male	red	good
3909	2012	Bredgrund	B3	62,00	male	red	good
3910	2012	Bredgrund	B3	63,20	male	red	good
3911	2012	Bredgrund	B3	65,40	male	red	good
3912	2012	Bredgrund	B3	66,00	male	red	good
3913	2012	Bredgrund	B3	67,50	male	green	good
3914	2012	Bredgrund	B3	42,50	female	red	good
3915	2012	Bredgrund	B3	51,20	female	red	good
3916	2012	Bredgrund	B3	58,60	male	red	good
3917	2012	Bredgrund	B3	62,10	male	red	good
3918	2012	Bredgrund	B3	64,00	male	red	good
3919	2012	Bredgrund	B3	63,70	male	green	good
3920	2012	Bredgrund	B3	66,70	male	green	reduced
3921	2012	Bredgrund	B3	69,80	male	green	reduced
3922	2012	Bredgrund	B3	64,90	male	green	good
3923	2012	Bredgrund	B3	70,40	male	green	good
3924	2012	Bredgrund	B3	66,00	male	red	reduced
3925	2012	Bredgrund	B3	52,50	male	red	good
3926	2012	Bredgrund	B3	57,30	male	red	good
3927	2012	Bredgrund	B3	60,40	male	red	good
3928	2012	Bredgrund	B3	60,60	male	red	good
3929	2012	Bredgrund	B3	61,50	male	red	good
3930	2012	Bredgrund	B3	62,00	male	red	good
3931	2012	Bredgrund	B3	62,30	male	red	good
3932	2012	Bredgrund	B3	64,40	male	red	good
3933	2012	Bredgrund	B3	65,00	male	red	good
3934	2012	Bredgrund	B4	51,70	male	red	reduced
3935	2012	Bredgrund	B4	54,00	male	red	good
3936	2012	Bredgrund	B4	61,40	male	red	good
3937	2012	Bredgrund	B4	63,70	male	red	good
3938	2012	Bredgrund	B4	63,90	male	red	good
3939	2012	Bredgrund	B4	64,20	male	red	good
3940	2012	Bredgrund	B4	57,90	male	green	good
3941	2012	Bredgrund	B4	50,80	male	red	reduced
3942	2012	Bredgrund	B4	61,90	male	red	good
3943	2012	Bredgrund	B4	54,30	male	red	reduced
3944	2012	Bredgrund	B4	50,40	male	red	good
3945	2012	Bredgrund	B4	52,00	male	red	good
3946	2012	Bredgrund	B4	60,10	male	red	good
3947	2012	Bredgrund	B4	56,30	male	red	good
3948	2012	Bredgrund	B4	56,40	male	red	good
3949	2012	Bredgrund	B4	57,40	male	red	good

3950	2012	Bredgrund	B4	61,50	male	red	good
3951	2012	Bredgrund	B4	64,50	male	green	good
3952	2012	Bredgrund	B4	68,90	male	green	good
3953	2012	Bredgrund	B4	66,40	male	red	good
3954	2012	Bredgrund	B4	60,30	male	green	good
3955	2012	Bredgrund	B4	62,20	male	green	good
3956	2012	Bredgrund	B4	63,70	male	green	good
3957	2012	Bredgrund	B4	64,20	male	red	reduced
3958	2012	Bredgrund	B4	57,00	male	red	good
3959	2012	Bredgrund	B4	59,20	male	red	good
3960	2012	Bredgrund	B4	60,80	male	red	good
3961	2012	Bredgrund	B4	61,90	male	red	good
3962	2012	Bredgrund	B4	63,60	male	red	good
3963	2012	Bredgrund	B4	65,10	male	red	good
3964	2012	Bredgrund	B5	59,70	male	red	good
3965	2012	Bredgrund	B5	40,60	female	red	good
3966	2012	Bredgrund	B5	61,90	male	red	good
3967	2012	Bredgrund	B5	56,30	male	red	reduced
3968	2012	Bredgrund	B5	64,70	male	green	good
3969	2012	Bredgrund	B5	67,80	male	green	good
3970	2012	Bredgrund	B5	59,70	male	red	reduced
3971	2012	Bredgrund	B5	61,20	male	red	good
3972	2012	Bredgrund	B5	63,60	male	red	good
3973	2012	Bredgrund	B5	69,70	male	red	good
3974	2012	Bredgrund	B5	56,50	male	red	good
3975	2012	Bredgrund	B5	61,20	male	red	good
3976	2012	Bredgrund	B5	61,60	male	red	good
3977	2012	Bredgrund	B5	65,40	male	red	good
3978	2012	Bredgrund	B5	68,00	male	red	good
3979	2012	Bredgrund	B5	51,30	male	green	good
3980	2012	Bredgrund	B5	65,60	male	green	good
3981	2012	Bredgrund	B5	68,90	male	green	good
3982	2012	Bredgrund	B5	74,50	male	green	good
3983	2012	Bredgrund	B5	56,80	male	red	reduced
3984	2012	Bredgrund	B5	60,00	male	red	reduced
3985	2012	Bredgrund	B5	62,80	male	red	reduced
3986	2012	Bredgrund	B5	64,30	male	red	reduced
3987	2012	Bredgrund	B5	67,20	male	red	reduced
3988	2012	Bredgrund	B5	45,80	female	red	good
3989	2012	Bredgrund	B5	51,60	male	red	good
3990	2012	Bredgrund	B5	60,00	male	red	good
3991	2012	Bredgrund	B5	60,70	male	red	good
3992	2012	Bredgrund	B5	63,40	male	red	good
3993	2012	Bredgrund	B5	65,20	male	red	good
3994	2012	Bredgrund	B5	69,80	male	red	good
3995	2012	Bredgrund	B6	51,90	male	green	good
3996	2012	Bredgrund	B6	57,80	male	red	good
3997	2012	Bredgrund	B6	65,90	male	red	reduced
3998	2012	Bredgrund	B6	58,70	male	green	good
3999	2012	Bredgrund	B6	65,10	male	green	good
4000	2012	Bredgrund	B6	65,10	male	green	good
4001	2012	Bredgrund	B6	69,30	male	green	good
4002	2012	Bredgrund	B6	56,50	male	red	reduced
4003	2012	Bredgrund	B6	52,40	female	red	good
4004	2012	Bredgrund	B6	63,70	male	red	good
4005	2012	Bredgrund	B6	65,10	male	red	good
4006	2012	Bredgrund	B6	65,20	male	green	good
4007	2012	Bredgrund	B6	66,60	male	green	good
4008	2012	Bredgrund	B6	64,80	male	red	reduced

4009	2012	Bredgrund	B6	59,10	male	red	good
4010	2012	Bredgrund	B6	66,10	male	red	good
4011	2012	Bredgrund	B6	66,40	male	red	good
4012	2012	Bredgrund	B6	59,80	male	green	good
4013	2012	Bredgrund	B6	62,00	male	green	good
4014	2012	Bredgrund	B6	63,50	male	green	good
4015	2012	Bredgrund	B6	64,30	male	green	good
4016	2012	Bredgrund	B6	66,10	male	green	good
4017	2012	Bredgrund	B6	67,50	male	green	good
4018	2012	Bredgrund	B6	68,60	male	green	good
4019	2012	Bredgrund	B6	72,90	male	green	good
4020	2012	Bredgrund	B6	55,80	male	red	reduced
4021	2012	Bredgrund	B6	57,80	male	red	reduced
4022	2012	Bredgrund	B6	62,20	male	red	reduced
4023	2012	Bredgrund	B6	63,20	male	red	reduced
4024	2012	Bredgrund	B6	63,20	male	red	reduced
4025	2012	Bredgrund	B6	56,50	male	red	good
4026	2012	Bredgrund	B6	57,60	male	red	good
4027	2012	Bredgrund	B6	58,60	male	red	good
4028	2012	Bredgrund	B6	58,70	male	red	good
4029	2012	Bredgrund	B6	59,80	male	red	good
4030	2012	Bredgrund	B6	59,80	male	red	good
4031	2012	Bredgrund	B6	60,20	male	red	good
4032	2012	Bredgrund	B6	60,90	male	red	good
4033	2012	Bredgrund	B6	61,30	male	red	good
4034	2012	Bredgrund	B6	62,40	male	red	good
4035	2012	Bredgrund	B6	65,10	male	red	good
4036	2012	Bredgrund	B6	70,40	male	red	good
4037	2012	Bredgrund	B7	43,30	female	red	reduced
4038	2012	Bredgrund	B7	61,30	male	green	good
4039	2012	Bredgrund	B7	58,90	male	red	reduced
4040	2012	Bredgrund	B7	58,70	male	red	good
4041	2012	Bredgrund	B7	64,20	male	green	reduced
4042	2012	Bredgrund	B7	55,60	male	green	good
4043	2012	Bredgrund	B7	60,70	male	red	reduced
4044	2012	Bredgrund	B7	61,50	male	red	reduced
4045	2012	Bredgrund	B7	45,60	female	red	good
4046	2012	Bredgrund	B7	47,80	female	red	good
4047	2012	Bredgrund	B7	54,10	male	red	good
4048	2012	Bredgrund	B7	58,80	male	red	good
4049	2012	Bredgrund	B7	65,90	male	red	good
4050	2012	Bredgrund	B7	58,70	male	green	good
4051	2012	Bredgrund	B7	66,70	male	green	good
4052	2012	Bredgrund	B7	63,70	male	red	reduced
4053	2012	Bredgrund	B7	65,80	male	red	reduced
4054	2012	Bredgrund	B7	52,70	male	red	good
4055	2012	Bredgrund	B7	59,39	male	red	good
4056	2012	Bredgrund	B7	61,70	male	red	good
4057	2012	Bredgrund	B7	61,70	male	red	good
4058	2012	Bredgrund	B7	62,60	male	red	good
4059	2012	Bredgrund	B7	63,00	male	red	good
4060	2012	Bredgrund	B7	64,40	male	red	good
4061	2012	Bredgrund	B7	65,70	male	red	good
4062	2012	Bredgrund	B7	67,70	male	red	good
4063	2012	Bredgrund	B7	61,00	male	green	reduced
4064	2012	Bredgrund	B7	71,70	male	green	reduced
4065	2012	Bredgrund	B7	59,90	male	green	good
4066	2012	Bredgrund	B7	60,60	male	green	good
4067	2012	Bredgrund	B7	62,90	male	green	good

4068	2012	Bredgrund	B7	63,10	male	green	good
4069	2012	Bredgrund	B7	63,50	male	green	good
4070	2012	Bredgrund	B7	65,50	male	green	good
4071	2012	Bredgrund	B7	66,90	male	green	good
4072	2012	Bredgrund	B7	59,60	male	red	reduced
4073	2012	Bredgrund	B7	51,40	female	red	good
4074	2012	Bredgrund	B7	51,60	male	red	good
4075	2012	Bredgrund	B7	55,60	male	red	good
4076	2012	Bredgrund	B7	57,60	male	red	good
4077	2012	Bredgrund	B7	58,10	male	red	good
4078	2012	Bredgrund	B7	58,40	male	red	good
4079	2012	Bredgrund	B7	62,00	male	red	good
4080	2012	Bredgrund	B7	62,30	male	red	good
4081	2012	Bredgrund	B7	62,60	male	red	good
4082	2012	Bredgrund	B7	63,50	male	red	good
4083	2012	Bredgrund	B7	68,40	male	red	good
4084	2012	Bredgrund	B8	53,40	male	red	good
4085	2012	Bredgrund	B8	68,60	male	red	good
4086	2012	Bredgrund	B8	50,20	male	green	good
4087	2012	Bredgrund	B8	54,40	male	red	reduced
4088	2012	Bredgrund	B8	56,30	male	red	reduced
4089	2012	Bredgrund	B8	58,30	male	red	reduced
4090	2012	Bredgrund	B8	55,40	male	red	good
4091	2012	Bredgrund	B8	56,70	male	red	good
4092	2012	Bredgrund	B8	59,00	male	red	good
4093	2012	Bredgrund	B8	67,10	male	green	reduced
4094	2012	Bredgrund	B8	66,10	male	green	good
4095	2012	Bredgrund	B8	66,70	male	green	good
4096	2012	Bredgrund	B8	39,00	female	red	good
4097	2012	Bredgrund	B8	56,80	male	red	good
4098	2012	Bredgrund	B8	58,80	male	green	good
4099	2012	Bredgrund	B8	61,60	male	green	good
4100	2012	Bredgrund	B8	65,20	male	green	good
4101	2012	Bredgrund	B8	65,40	male	green	good
4102	2012	Bredgrund	B8	67,10	male	green	good
4103	2012	Bredgrund	B8	69,40	male	green	good
4104	2012	Bredgrund	B8	71,40	male	green	good
4105	2012	Bredgrund	B8	56,30	male	red	reduced
4106	2012	Bredgrund	B8	56,50	male	red	reduced
4107	2012	Bredgrund	B8	58,10	male	red	reduced
4108	2012	Bredgrund	B8	42,90	female	red	good
4109	2012	Bredgrund	B8	57,90	male	red	good
4110	2012	Bredgrund	B8	58,50	male	red	good
4111	2012	Bredgrund	B8	59,20	male	red	good
4112	2012	Bredgrund	B8	59,80	male	red	good
4113	2012	Bredgrund	B8	60,00	male	red	good
4114	2012	Bredgrund	B8	60,80	male	red	good
4115	2012	Bredgrund	B8	63,90	male	red	good
4116	2012	Bredgrund	B8	64,90	male	red	good
4117	2012	Bredgrund	B8	67,60	male	red	good
4118	2012	Bredgrund	B8	68,20	male	green	reduced
4119	2012	Bredgrund	B8	59,70	male	green	good
4120	2012	Bredgrund	B8	59,90	male	green	good
4121	2012	Bredgrund	B8	61,20	male	green	good
4122	2012	Bredgrund	B8	61,90	male	green	good
4123	2012	Bredgrund	B8	62,40	male	green	good
4124	2012	Bredgrund	B8	64,10	female	red	reduced
4125	2012	Bredgrund	B8	57,20	male	red	reduced
4126	2012	Bredgrund	B8	50,00	male	red	good

4127	2012	Bredgrund	B8	53,70	male	red	good
4128	2012	Bredgrund	B8	55,00	male	red	good
4129	2012	Bredgrund	B8	58,40	male	red	good
4130	2012	Bredgrund	B8	60,00	male	red	good
4131	2012	Bredgrund	B8	60,40	male	red	good
4132	2012	Bredgrund	B8	61,00	male	red	good
4133	2012	Bredgrund	B8	61,10	male	red	good
4134	2012	Bredgrund	B8	62,50	male	red	good
4135	2012	Bredgrund	B8	63,00	male	red	good
4136	2012	Bredgrund	B8	63,30	male	red	good
4137	2012	Bredgrund	B8	66,30	male	red	good
4138	2012	Bredgrund	B8	67,20	male	red	good
4139	2012	Bredgrund	B9	58,80	male	green	good
4140	2012	Bredgrund	B9	63,90	female	red	reduced
4141	2012	Bredgrund	B9	44,00	male	green	good
4142	2012	Bredgrund	B9	60,80	female	red	reduced
4143	2012	Bredgrund	B9	59,20	male	red	good
4144	2012	Bredgrund	B9	63,20	male	green	reduced
4145	2012	Bredgrund	B9	68,40	male	green	good
4146	2012	Bredgrund	B9	64,30	male	red	reduced
4147	2012	Bredgrund	B9	64,30	male	red	good
4148	2012	Bredgrund	B9	62,80	male	green	good
4149	2012	Bredgrund	B9	56,40	male	red	good
4150	2012	Bredgrund	B9	60,20	male	red	good
4151	2012	Bredgrund	B9	58,50	male	green	good
4152	2012	Bredgrund	B9	58,60	male	green	good
4153	2012	Bredgrund	B9	61,20	male	green	good
4154	2012	Bredgrund	B9	65,40	male	green	good
4155	2012	Bredgrund	B9	66,20	male	green	good
4156	2012	Bredgrund	B9	59,70	male	red	reduced
4157	2012	Bredgrund	B9	62,70	male	red	good
4158	2012	Bredgrund	B9	67,70	male	red	good
4159	2012	Bredgrund	B9	60,10	male	green	good
4160	2012	Bredgrund	B9	62,90	male	green	good
4161	2012	Bredgrund	B9	64,70	male	green	good
4162	2012	Bredgrund	B9	70,90	male	green	good
4163	2012	Bredgrund	B9	56,10	male	red	reduced
4164	2012	Bredgrund	B9	59,50	male	red	reduced
4165	2012	Bredgrund	B9	60,10	male	red	reduced
4166	2012	Bredgrund	B9	59,90	male	red	good
4167	2012	Bredgrund	B9	63,50	male	red	good
4168	2012	Bredgrund	B9	63,90	male	red	good
4169	2012	Bredgrund	B9	63,90	male	red	good
4170	2012	Bredgrund	B9	64,50	male	red	good
4171	2012	Bredgrund	B9	66,20	male	red	good
4172	2012	Bredgrund	B9	66,30	male	red	good
